# Trends in Application of SERS Substrates beyond Ag and Au, and Their Role in Bioanalysis

**DOI:** 10.3390/bios12110967

**Published:** 2022-11-03

**Authors:** Alisher Sultangaziyev, Aisha Ilyas, Aigerim Dyussupova, Rostislav Bukasov

**Affiliations:** Department of Chemistry, School of Sciences and Humanities (SSH) Nazarbayev University, Kabanbay Batyr av. 53, Astana 010000, Kazakhstan

**Keywords:** SERS, clinical applications, LOD, clinical sensitivity, detection of biomarkers, immunoassays, silicon, aluminum

## Abstract

This article compares the applications of traditional gold and silver-based SERS substrates and less conventional (Pd/Pt, Cu, Al, Si-based) SERS substrates, focusing on sensing, biosensing, and clinical analysis. In recent decades plethora of new biosensing and clinical SERS applications have fueled the search for more cost-effective, scalable, and stable substrates since traditional gold and silver-based substrates are quite expensive, prone to corrosion, contamination and non-specific binding, particularly by S-containing compounds. Following that, we briefly described our experimental experience with Si and Al-based SERS substrates and systematically analyzed the literature on SERS on substrate materials such as Pd/Pt, Cu, Al, and Si. We tabulated and discussed figures of merit such as enhancement factor (EF) and limit of detection (LOD) from analytical applications of these substrates. The results of the comparison showed that Pd/Pt substrates are not practical due to their high cost; Cu-based substrates are less stable and produce lower signal enhancement. Si and Al-based substrates showed promising results, particularly in combination with gold and silver nanostructures since they could produce comparable EFs and LODs as conventional substrates. In addition, their stability and relatively low cost make them viable alternatives for gold and silver-based substrates. Finally, this review highlighted and compared the clinical performance of non-traditional SERS substrates and traditional gold and silver SERS substrates. We discovered that if we take the average sensitivity, specificity, and accuracy of clinical SERS assays reported in the literature, those parameters, particularly accuracy (93–94%), are similar for SERS bioassays on AgNP@Al, Si-based, Au-based, and Ag-based substrates. We hope that this review will encourage research into SERS biosensing on aluminum, silicon, and some other substrates. These Al and Si based substrates may respond efficiently to the major challenges to the SERS practical application. For instance, they may be not only less expensive, e.g., Al foil, but also in some cases more selective and sometimes more reproducible, when compared to gold-only or silver-only based SERS substrates. Overall, it may result in a greater diversity of applicable SERS substrates, allowing for better optimization and selection of the SERS substrate for a specific sensing/biosensing or clinical application.

## 1. Introduction

Since its discovery in 1976–1977 [1,2], surface-enhanced Raman spectroscopy (SERS) has become a powerful analytical technique, widely used for the detection of various analytes at low concentrations. In comparison to many other analytical methods, SERS is a highly sensitive, fast, humidity-independent analytical method with a high potential for multiplexed detection [3,4]. In addition, SERS advantages over fluorescence, for instance, include good robustness/low photobleaching and capabilities for label-free detection, while SERS can be applied for in situ monitoring, in vivo biosensing, and even single molecule detection [5,6,7,8,9]. Due to these advantages, SERS is widely used in molecular biology [10,11,12,13], biomedicine [14,15,16], and environmental science [17]. Furthermore, SERS is capable of detecting single molecules [18,19,20,21]. However, factors such as pH, the degree of nanoparticle (NP) aggregation, temperature and substrate composition can have a significant impact on the reproducibility and enhancement of SERS applications [22].

The main idea behind SERS is to improve Raman scattering through two different mechanisms: (a) electromagnetic field enhancement and (b) chemical enhancement. The light-induced electric fields on the surface of the metal nanoparticle cause electromagnetic enhancement. These enhanced electric fields are produced when the incident light is in resonance with the oscillations of the metal nanoparticle’s conduction electrons, causing them to oscillate collectively. This optical phenomenon is called localized surface plasmon resonance (LSPR) [23]. Electromagnetic enhancement increases the Raman scattering at least by a factor of 10^4^–10^6^. On the other hand, chemical enhancement arises from the localized electronic resonance of adsorbate or charge transfer resonance from the surface to metal nanoparticles and gives the enhancement of 10^2^–10^4^ [24]. Hence the nature of metal nanoparticles and substrates plays an important part in their plasmonic properties [25], therefore affecting SERS signal reproducibility and enhancement.

In the last 40 years, the main substrates of choice for the SERS and other surface-enhanced spectroscopic techniques were noble metals such as gold and silver, as they possess the ability to provide and sustain intense plasmon resonances in the visible-near infrared region where the wavelength range of interest for Raman measurements is located [26,27]. However, gold and silver film substrates have not only a relatively high cost as a disadvantage, but they may also be susceptible to corrosion, recrystallization, and biodegradation [28,29,30], as well as contamination with mercapto-containing compounds and hydrocarbons, which requires special cleaning/pretreatment [31,32,33]. Thus, the search for more cost-efficient and robust SERS substrates is a topic of high importance, as the number and scope of SERS applications increase each year [34].

One of the possible alternatives for conventional plasmonic metals is aluminum, which is plasmon tunable in a relatively broad UV and visible range and passivated by a thin layer of oxide [35]. There were reports of the application of aluminum as inexpensive, versatile and sensitive substrate for SERS, with excitation in the visible range [35,36,37,38,39,40]. In addition, Al was reported as a substrate for Surface-Enhanced Fluorescence (SEF) spectroscopy, which could compete with and sometimes outperform typical SEF substrates such as gold and silver [41,42]. Moreover, according to several studies the use of less conventional non-noble metal substrates such as copper, palladium, and platinum in combination with gold and silver nanostructures has great potential for SERS applications [43,44,45,46]. In addition to metal substrates, the use of silicon wafers as a SERS substrate is quite widespread and has shown promising results in several studies [47,48,49,50]. It is significantly cheaper than plasmonic metal substrates and has remarkable stability on par with biocompatibility [51]. Therefore, it can be seen that there is a significant number of studies introducing new SERS substrates with a variety of applications. Henceforth, the review combining these studies and comparing them with conventional substrates will be beneficial for the future development of novel SERS substrates.

In this review, we combined numerous studies demonstrating the SERS applications of non-conventional substrates beyond gold and silver. We started with examples of standalone metallic substrates and then explored their combinations with gold and silver nanostructures. Next, we presented applications of a semiconductor material such as silicon and non-noble metal substrate such as aluminum, with and without modification by nanostructures. Next, we delve into the area of new substrates using alloys and other metals. Finally, we compared these novel metal substrates to traditional gold and silver substrates using figures of merit such as enhancement factors (EFs) and limits of detection (LOD). In addition, we described SERS-based clinical diagnostic applications of non-conventional substrates (e.g., silicon and aluminum) and compared them with similar applications of gold and silver substrates in terms of the accuracy, specificity, and sensitivity, when those analytical parameters are reported. Overall, this review demonstrated recent trends in this research area and suggested possible directions for future development of SERS substrates.

## 2. Pt/Pd and Other Noble Metals as SERS Substrates

Platinum and Palladium are noble transition metals, well known for their catalytic and electrochemical properties and wide applicability in the photo- and electrocatalysis [52,53,54]. These metals have been commonly considered non-active substrates due to their interband excitation in the visible range that causes quenching effect of surface plasmon resonance (SPR) [55]. SPR is regarded as the main reason for the enhancement of signals in SERS in comparison to Raman. Platinum or palladium surfaces can be electrochemically roughened to attain an improved SERS performance [56,57]. One of the earliest works applying the roughened platinum to acquire the SERS measurement was carried out by Tian et al., who also contributed to this field significantly. The weak SERS enhancement of 10 to 120 was observed for the roughened platinum surface in the detection of pyrimidine in 1996 [58]. SERS activity of Pt and Pd can be enhanced by so-called borrowing effect, where Au and Ag-based active metal cores are coated with Pt and Pd shells to avoid quenching of SPR [59]. In 1998, Weaver M.J. electrochemically deposited thin films of Pt, Pd, Rh, and Ir onto gold, and “pinhole-free” surfaces were obtained to avoid the spectral and chemical interferences from the substrate. Generally, all the electrochemically modified Pt group metals exhibited the enhancement of signals, and it was identified that the intensity of the SERS signal is inversely proportional to the thickness of the metal film. In addition, the SERS intensities of benzonitrile on Pt film on gold and unmodified gold were comparable to each other. However, Weaver claims that the presence of pinholes on the surface of the transition metal films is highly likely to make error-prone SERS measurements and that substrates should be fabricated carefully to avoid the holes [60].

Since pure platinum and palladium are not SERS active, these transition metals are most likely to be combined with conventional SERS substrates [61,62,63,64] or the alloy of metals. Frequently, the Pt/Pd films or nanoparticles reside on the surface of Si layer and rarely the bare Pt/Pd nanoparticles are used as a substrate for SERS measurements. For the examination of the transition metals, platinum and palladium, 27 research papers were selected. The average EF and LOD of these papers can be found in Table 1, while the individual data about each study can be found in Supplementary Materials. The SERS activities of platinum and palladium were also measured separately, and the results are also available in Table 1.

The geometric average EF of Pt, Pd group of substrates is 1.4 × 10^5^, which is lower than Au/Ag substrates and Si-based substrates by two orders of magnitude. The limit of detection of Pt/Pd-based substrates is also 100 times worse than the previously discussed Au, Ag, and Si groups being around 2.9 × 10^−9^ M. In 2002, Tian et al. recorded SERS signals of pyridine on 6 metals including Pt and Au [56]. Over the course of the study, it was identified that the enhancement of gold was about 500 times better than that of Pt, having an EF of 10^6^ for gold and 2000 for Pt. Other metal nanoparticles of Rh, Fe, Ni, and Co had an enhancement similar to platinum. Another proof of the superiority of gold over platinum as the SERS substrate can be the work published by Kim Kwan, where 4-aminobenzenethiol was sandwiched between silver nanoparticles and Au or Pt surface. Researchers reported that when 4-ABT was measured on a Pt surface, no SERS signals were obtained and only after sandwiching with AgNP, enhancement of 790 was achieved. In case of Au, surface EF was 1.7 × 10^4^ [63]. In a different experiment where the Raman spectroscopy of p-ATP on a Pt film was measured, no signal peaks were observed. Therefore, silver nanocubes were applied to sandwich the analyte and consequently the best gained EF was 4.1 × 10^6^. The authors state that with an increase in thickness of Pt film from 42 nm to 90 nm, the enhancement factor has grown by about three times. In the same paper, the AgNC@p-ATP@glass surface substrate was applied and in comparison to the glass surface, the platinum substrate provided 15 times more enhancement [64].

**Table 1:** The average SERS performance of Si, Pt, Pd, Al, Cu-based substrates and conventional pure Ag, Au substrates.

Substrate	Average EF (Min; Max)		Average LOD, M (Min; Max)		References
	Arithmetic	Geometric	Arithmetic	Geometric	
Si without metals	$1.11\times 10^{7}$ $\left(500;4\times \;10^{7}\right)$	$2.55\times 10^{5}$ $\left(500;\;4\times 10^{7}\right)$	$2.53\times 10^{-5}$ $\left(10^{-12};10^{-4}\right)$	$4.85\times 10^{-9}$ $\left(10^{-12};10^{-4}\right)$	[65,66,67,68,69,70]
Si with metals	$1.59\times 10^{13}$ $\left(33;\;10^{15}\right)$	$1.44\times 10^{7}$ $\left(33;\;10^{15}\right)$	$5.05\times 10^{-8}$ $\left(10^{-18};2\times 10^{-6}\right)$	$1.57\times 10^{-11}$ $\left(10^{-18};2\times 10^{-6}\right)$	[50,55,66,71,72,73,74,75,76,77,78,79,80,81,82,83,84,85,86,87,88,89,90,91,92,93,94,95,96,97,98,99,100,101,102,103,104,105,106,107,108,109,110,111,112,113,114,115,116,117,118,119,120,121,122,123,124,125,126,127,128,129,130,131,132,133,134]
Overall Si	$1.48\times 10^{13}$ $\left(33;\;10^{15}\right)$	$1.10\times 10^{7}$ $\left(33;\;10^{15}\right)$	$1.79\times 10^{-6}$ $\left(10^{-18};10^{-4}\right)$	$2.30\times 10^{-11}$ $\left(10^{-18};10^{-4}\right)$	[50,55,65,66,67,68,69,70,71,72,73,74,75,76,77,78,79,80,81,82,83,84,85,86,87,88,89,90,91,92,93,94,95,96,97,98,99,100,101,102,103,104,105,106,107,108,109,110,111,112,113,114,115,116,117,118,119,120,121,122,123,124,125,126,127,128,129,130,131,132,133,134]
Pt	$3.94\times 10^{8}$ $\left(33;\;4.7\times 10^{9}\right)$	$1.23\times 10^{5}$ $\left(33;\;4.7\times 10^{9}\right)$	$6.67\times 10^{-6}$ $\left(10^{-15};2\times 10^{-5}\right)$	$2.71\times 10^{-10}$ $\left(10^{-15};2\times 10^{-5}\right)$	[55,56,62,63,64,97,101,135,136,137,138,139]
Pd	$1.04\times 10^{8}$ $\left(10^{2};1.9\times 10^{9}\right)$	$1.38\times 10^{5}$ $\left(100;\;1.9\times 10^{9}\right)$	$1.02\times 10^{-6}$ $\left(10^{-11};10^{-5}\right)$	$4.57\times 10^{-9}$ $\left(10^{-11};10^{-5}\right)$	[55,57,61,62,98,99,100,140,141,142,143,144,145,146,147,148,149]
Overall Pt/Pd	$1.97\times 10^{8}$ $\left(33;\;1.9\times 10^{9}\right)$	$1.39\times 10^{5}$ $\left(33;\;1.9\times 10^{9}\right)$	$2.15\times 10^{-6}$ $\left(10^{-11};10^{-5}\right)$	$2.86\times 10^{-9}$ $\left(10^{-11};10^{-5}\right)$	[55,56,57,61,62,63,64,97,98,99,100,101,135,136,137,138,139,140,141,142,143,144,145,146,147,148,149]
Pure Al	3.4 × 10^5^ (5 × 10^3^; 10^6^)	1.27 × 10^5^ (5 × 10^3^; 10^6^)	1.03 × 10^−6^ (10^−7^; 2 × 10^−6^)	5.85 × 10^−7^ (10^−7^; 2 × 10^−6^)	[150,151,152,153,154,155,156]
Al + Au (R6G analyte)	8.1 × 10^6^ (only one EF)		7 × 10^−10^ (10^−10^; 10^−9^)	3.16 × 10^−10^ (10^−10^; 10^−9^)	[157,158,159,160]
Al + Ag (R6G analyte)	5.39 × 10^7^ (10^7^; 9.77 × 10^7^)	3.13 × 10^7^ (10^7^; 9.77 × 10^7^)	2.50 × 10^−7^ (10^−15^; 10^−6^)	1.78 × 10^−11^ (10^−15^; 10^−6^)	[161,162,163,164,165]
Pure Cu	7.36 × 10^6^ (10^4^; 4.70 × 10^7^)	1.25 × 10^6^ (10^4^; 4.70 × 10^7^)	4.46 × 10^−7^ (10^−9^; 10^−6^)	7.2 × 10^−8^ (10^−9^; 10^−6^)	[44,45,166,167,168,169,170]
Cu + Au	2.52 × 10^5^ (1.2 × 10^3^; 5.0 × 10^5^)	7.81 × 10^4^ (1.2 × 10^3^; 5.0 × 10^5^)	3.33 × 10^−4^ (10^−10^; 10^−3^)	10^−7^ (10^−10^; 10^−3^)	[171,172,173,174,175]
Cu + Ag	6.97 × 10^10^ (1.19 × 10^5^; 4.88 × 10^11^)	1.76 × 10^7^ (1.19 × 10^5^; 4.88 × 10^11^)	5.01 × 10^−9^ (10^−21^; 3.30 × 10^−8^)	5.3 × 10^−12^ (10^−21^; 3.30 × 10^−8^)	[169,176,177,178,179,180,181]
Other metals (Zn, Ti, Fe, Co, Mo, Cr, Hf + Au, Ag)	6.85 × 10^7^ (2.70 × 10^7^; 9.40 × 10^7^)	6.14 × 10^7^ (2.70 × 10^7^; 9.40 × 10^7^)	2.96 × 10^−7^ (10^−12^; 2.0 × 10^−6^)	4.43 × 10^−8^ (10^−12^; 1.0)	[182,183,184,185,186,187,188,189,190,191]
Au	$3.91\times 10^{8}$ $\left(2\times 10^{6},\;10^{9}\right)$	$7.54\times 10^{7}$ $\left(2\times 10^{6},\;10^{9}\right)$	$2.17\times 10^{-9}$ ($10^{-13}$,$10^{-7})$	$2.64\times 10^{-11}$ ($10^{-13}$,$10^{-7})$	[50,192,193,194]
Ag	$5.07\times 10^{7}$ ($9\times 10^{5},2\times \;10^{8})$	$1.35\times 10^{7}$ ($9\times 10^{5},2\times \;10^{8})$	$4.07\times 10^{-8}$ $\left(10^{-14};10^{-7}\right)$	$3.36\times 10^{-10}$ $\left(10^{-14};10^{-7}\right)$	[195,196,197,198]
Overall Au/Ag	$6.40\times 10^{8}$ $\left(9\times 10^{5},\;6\times \;10^{9}\right)$	$6.44\times 10^{7}$ ($9\times 10^{5},6\times \;10^{9})$	$9.87\times 10^{-9}$ $\left(10^{-16};10^{-7}\right)$	$1.04\times 10^{-11}$ $\left(10^{-16};10^{-7}\right)$	[199,200]

Abbreviations: EF—enhancement factor; LOD—limit of detection; R6G—rhodamine 6G.

The difference in performance of platinum and palladium substrates was not established based on mean values of EFs and LODs. However, according to Hu et al., Pd films deposited on the Au sphere segment void produce two times more enhancement than the Pt film, with EF 7.8 × 10^3^ and 4.9 × 10^3^, correspondingly [62]. Tran et al. also determined a similar tendency, where PdNP on porous silicon had three times higher EF than its counterpart, more precisely EF was 100 and 33, respectively [55]. By analyzing these two papers it can be assumed that palladium can be a better substrate material than platinum; these two metals when coupled with gold deliver better SERS measurements than silicon.

For example, Shvalya et al. presented a SERS-based detection method with reusable Au&Pd alloy@Cu_2_O/CuO substrate [140]. This feature makes the substrate both cost-efficient and sustainable. According to Shvalya et al., to prepare this SERS substrate, firstly, copper plate was textured by thermal annealing in an oxygen gas atmosphere forming chest-like Cu_2_O/CuO surface. Next, to plasmonically activate the copper surface, an alloy containing 60% Au and 40% Pd by weight was ionically sputtered. After taking SERS measurements of the first analyte, the re-usage of the substrate was attainable by cleaning in the oxygen/argon plasma to degrade the analyte on the substrate surface. The presence of peaks on the SERS spectrum of R6G, and the absence of them after the exposure to plasma cleaning showed that the substrate can be used once again. The substrate was 100% self-recovered in 1 min; enhancement factor was about 500 thousand. Down to one micromolar crystal violet was detected. The comparison of grainy-like (with wide gaps) and chestnut-like textures (with small gaps) revealed that the latter resulted in better enhancement of the signal. When the interparticle distance is short as in chestnut-like textures, optically induced electromagnetic field generated by separated facets interfere and provide a highly intense electromagnetic field. Considering the reusability advantage, this substrate may have the potential for biosensing applications [140].

Ag-Pd alloy nanostructures were integrated inside the microfluidic channels using the femtosecond laser direct writing fabrication technique. Integrating the substrate within the microfluidic channel enabled on-chip detection and in situ monitoring of chemical reactions. Furthermore, the microfluidic system may have facilitated the SERS process, proving the detection down to 1 nM of analyte. In addition, Ag-Pd alloy assisted in the stability against aerobic oxidation [147].

In addition, while discussing pro and cons of SERS substrates, we should consider the cost and availability of noble metals such as platinum and palladium. For instance, 3 nm PtNP cost €318 at Sigma-Aldrich (773875-25 ML) [201], while 500 mg of <25 nm PdNP is €981 (686468-500 MG) [202], and these are the only available sizes on the website. At the same time, AgNPs are available in different sizes ranging from 10 to 100 nm, and their cost is about €141 (for 10 nm, 730777-25 ML) [203], while 3 to 300 nm AuNP can be purchased for €116–€206, and 10 nm AuNP for €128 (741965-25 ML) [204]. Gold and silver are more available and at least two times more cost-effective than Platinum and several times less expensive than Palladium. Overall, since SERS performance parameters (EF and LOD) provided by Pt and Pd-based substrates are lower than that of Au and Ag substrates by about two orders of magnitude, and their cost is higher than gold and especially silver, these substrates do not appear to be a serious alternative to replace the conventional gold and silver-based SERS substrates.

## 3. Copper

Copper-based substrates have promising application prospects in SERS measurements mainly due to the low cost, high SERS activity, and strong chemisorption of most analytes [43]. In terms of surface enhancement, copper nanostructures can compete with silver and gold [44]. For example, pure copper-based SERS substrates on average are capable of producing surface enhancement of ~10^6–7^, while silver and gold produce on average EF 10^8–9^ (See Table 2). Compared with silver and gold, which cannot support surface plasmons in the ultraviolet region, copper meet this criterion and so has a wider range of applications. However, due to its surface oxidation and instability [205], it is not widely used in plasmonic applications as silver and gold. Though there exist various methods to protect copper from surface oxidation. For example, the group of Dr. Van Duyne found that glacial acetic acid is effective for removing the layer of Cu oxides (Cu_2_O and CuO) without modifying the metal surface. They showed that upon the removal of the oxide layer comparatively narrower and more intense LSPR peak can be obtained which is comparable to Au and Ag when λ_max_ > ~650 nm [206].

The research group of Dr. Van Duyne was one of the first groups that used Cu for the fabrication of SERS substrate [207]. Back in the 1980s, Cu electrodes were predominantly used for SERS-based measurements [208,209]. For example, in 1980 the group of Dr. Van Duyne studied the wavelength dependence of surface-enhanced Raman scattering of pyridine on copper electrode surfaces and figured out that it is strongly correlated with the optical properties of copper metal. The enhancement factor (EF) for the pyridine/Cu system was 6 × 10^4^ at a 645 nm excitation wavelength. Years later, Cu island films and alloys attracted significant interest as SERS active substrates [210,211]. For example, Kudelski et al. used amorphous Cu-Zr alloys for in-situ SERS measurement of adsorbed pyridine [211]. They showed that by using the potential dependence of SERS intensity, different copper clusters on the surface of the substrate can be identified. Nowadays, copper structures of different geometry and morphology (e.g., Cu nanowires, films, nanoparticles, copper triangle plates (CTPs), Cu-coated fabric, etc.) are used as SERS-active substrates.

At the moment, Cu-based SERS substrates are fabricated by different techniques such as lithography [212], oxidation-reduction cycles [213], cathodic reduction [214], chemical deposition [215], electrodeposition route [216], evaporation of a SERS-active metal on the surface of a rough substrate [217], chemical etching techniques [218]. Table 2 summarizes the results of analytical studies on copper-based substrates of different morphology from 2016 to 2021. Among these studies, we want to mention the work of Halouzka et al. By coating the carbon fiber microelectrodes with copper nanowires, they achieved EF 10^6^–10^7^ in the detection of trace amounts (10^−10^–10^−12^ M solutions) of cathionine designer drugs such as 4-mephedrone, 4-methylmethcathinone, and buphedrone [166]. Designer drugs are substances that cause similar effects as original drugs, but they are considered legal substances [219]. The list of legal designer drugs includes buphedrone, 1-benzyl-4-methylpiperazine (MBZP), 4′-Methoxy-αpyrrolidinopropiophenone (MOPPP), and para-methoxyphenylpiperazine (MOPP) containing substances. In addition to the detection of designer drugs, copper-coated microelectrodes can be used in the detection of biomolecules, and emerging pollutants, as prospective detectors in microfluidic devices and lab-on-the-chip systems [166]. This is possible mainly because of the 2.5–6 times decrease in the photoluminescence background signal due to quenching by copper nanostructure.

**Table 2:** List of studies of pure Cu-based SERS substrates from 2016 to 2021.

Substrate	Analyte	Analytical Parameters	Ref
Cu nanowire-coated carbon fibers	Designer drugs	EF 10^6^–10^7^	Halouzka et al., 2017 [166]
Cu/Cu_2_O core-shell NPs	CV, MB (532 nm)	EF 10^4^ RSD < 28%	H. Dizajghorbani et al., 2019 [44]
Cu films on the microstructural mantis wing (Cu/MW)	R6G, 4-ATP, CV	LOD 10^−6^ M EF 1.19 × 10^5^ RSD < 28%	Li et al., 2018 [45]
Copper oxide (CuO) nanoparticles	RhB	EF 1.5 × 10^5^ RSD 12%	Behzad Sardari & Meriç Özcan 2017 [220]
Copper triangle plates (CTPs)	RhB	EF 4.5 × 10^6^	Chang Li, Mingqiang Chen 2020 [167]
Cu nanoparticles on reduced graphene oxides (CuNPs/rGO)	R6G	LOD 10^−8^ M EF 2.75 × 10^6^ RSD < 10%	He et al., 2016 [168]
Cu-coated fabric	CV	LOD 10^−8^ M EF 2 × 10^6^	Dai et al., 2021 [169]
The geometric mean for LOD: 4.22 × 10^−8^ M, geometric mean for EF: 1.59 × 10^6^			

Abbreviations: MBZP—1-benzyl-4-methylpiperazine, MOPP—para-methoxyphenylpiperazine, CV –crystal violet, MB—methylene blue, R6G—rhodamine 6G, 4-ATP—4-aminothiophenol, RhB—rhodamine B.

Copper has also promising prospects to be chosen as a material for SERS platforms preparation, which is used in biosensing [221]. It is cost-efficient, physically more stable over time compared to silver [222], and can exhibit high enhancement factors in the range between 10^6–7^ similar to silver (EF 10^7–10^) and gold (EF~10^6^) [221,223]. In addition, copper-based SERS platforms can be used for bacterial detection. Kudelska et al. prepared a novel copper-based platform using a simple high-pressure method through the decomposition of copper hydride. The platform revealed a good enhancement of *S. aureus* bacteria bands, comparable with those observed on silver and gold-based platforms [221].

In another interesting research, Behzad Sardari and Meriç Özcan demonstrated that copper oxide (CuO) nanoparticles formed on copper electrodes by electrolysis can be used as a real-time active substrate for SERS [220]. CuO/Cu electrodes were capable of producing a low relative standard deviation (RSD) close to 12%. Dai et al. fabricated Cu-based SERS chips by chemical reduction method [169]. In the fabrication process, Cu nanoparticles have been deposited onto fabric supports to avoid random aggregation of colloidal copper. In this way, they achieved EF~10^6^ and low LOD 10^−8^ M using crystal violet (CV) as a probe molecule. He et al. achieved prominent SERS results by fabricating hydrophobic ligand-mediated hierarchical Cu nanoparticles on reduced graphene oxides (CuNPs/rGO), which exhibited low LOD 10^−8^ M and high EF~10^6^ for R6G as a probe molecule.

Last, but not least, it is worth mentioning the effective application of copper sulfide nanoparticles in the fabrication of SERS probes, which have received great attention recently, largely due to their great potential in biomedical applications. Currently reported SERS probes based on non-degradable Au or Ag nanostructuresa are not completely eliminated from the imaged tissue. Such long-term persistence raises multiple concerns regarding chronic toxicity due to a chance for SERS probes to aggregate, produce harmful metabolites, and repartition to other major organs (e.g., heart, liver, lungs, kidney [224]. This bottleneck hinders their further in-vivo application. On the contrary, SERS probes consisting of hollow copper sulfide (CuS) nanoparticles (NPs) circumvent this limitation because they are photodegradable. For example, in their recent study, Qui et al. found that SERS probes made of CuS NPs are capable of eradicating residual tumor lesions by hyperthermia and degradation into small particles, facilitating their self-clearance from tumor tissue [225]. They used orthotopic prostate cancer as a model disease. In the examination of elimination of CuS SERS probes from the main organs within 30 days, results have shown that at day 30 only 0.6, 0.4, 0.9, 0.5 and 0.3% ID/g (percentage of the injected dose per gram of the tissue) of Cu retained in heart, liver, spleen, lungs and kidneys, respectively. Moreover, CuS probes showed good targeting ability showing 3.6% ID/g of Cu in the tumor. These results demonstrate that CuS SERS probes were efficiently eliminated from the main organs, further justifying their biosafety. Similar observations have been reported in another comparative study of hollow copper sulfide nanoparticles and hollow gold nanospheres on degradability and toxicity [224]. The results of this study have shown that CuS NPs were effectively eliminated at day 30 and do not show toxicity in blood or histological chemistry analysis. On the contrary, Au NPs were long-term accumulated in the liver, which lead to high levels of serum lactate dehydrogenase, indicating long-term toxicity. Overall, the comparative advantage of CuS NPs SERS probes over Au or Ag-based ones in terms of their degradability opens avenues for applying SERS for a myriad of biomedical applications.

From all previously discussed studies, it can be seen that among noble metals copper has great application perspective as a SERS substrate. Numerous studies for the last several years have shown that due to its lower cost, ability to support surface plasmons in the ultraviolet region, strong chemisorption of most analytes, degradability and biosafety, copper-based SERS substrates can be used to detect various targets including biomolecules, designer drugs, pollutants, etc. However, one of the limiting factors of copper-based substrate is its susceptibility to surface oxidation. Due to the surface oxidation of most copper-based substrates, they are not as widely used in plasmonics as silver and gold.

### Copper with Nanostructured Silver and Gold

Synergistic effects of copper with silver or gold can produce SERS active substrates of better sensitivity, Raman enhancement, and stability [169,171,176]. From Table 1, it can be seen that upon the addition of silver nanostructures to copper-based substrates, EF increases by approximately one factor (from 10^6^ to 10^7^), while LOD decreases by a factor of three (from 10^−9^ M to 10^−12^ M). SERS performance of copper-based substrates with nanostructured silver and gold are listed in corresponding tables in Supplementary Material. In this section, some of the most prominent analytical studies on copper-based substrates with Ag or Au have been discussed.

Dai et al. demonstrated that with introduction of Ag onto the clean Cu surface, Ag-modified Cu chips exhibited a 10^3^ decrease in LOD of crystal violet (CV) from 10^−8^ M to 10^−11^ M as well as an increase in EF from 2.0 × 10^6^ to 7.6 × 10^6^ due to nanoscale plasmonic coupling between Cu and Ag [169]. Moreover, as opposed to 18% of the original Cu chips, Ag-modified Cu chips maintained 80% of the original intensity after 2 months of storing due to the protective shell of Ag. Fodjo et al. showed that for carbamate pesticides (e.g., carbaryl, carbofuran, isoprocarb, and propoxur) SERS substrate based on Ag/b-AgVO_3_ nanobelts deposited on copper foil (Cu@Ag/b-AgVO_3_) can achieve better SERS activity than Ag nanoparticles (NPs) alone. The fabricated substrate showed good repeatability for ten successive measurements of corresponding pesticides (RSD 4.3%) and long-term stability [176]. By measuring the SERS intensity of different microflower (MF) substrates, Sravani et al. demonstrated that with an increasing amount of Cu in the Ag-Cu alloy, the intensity of a probe molecule R6G gradually increases compared with pure Ag MFs and can achieve the highest SERS intensity at 10% Cu. They achieved a 50-fold enhancement in SERS spectra with 10% Ag-Cu alloy microflowers, which supersedes the efficiency of pure Au-Ag MFs [180]. Bimetallic nanostructures with tunable morphology are of current interest in plasmonics, catalysis and SERS [175]. In their study, Siva et al. fabricated gold−copper nanostructures (Au−Cu NSs) of 200–300 nm size range and revealed a high SERS signal for crystal violet with EF as high as 10^6^ and the ability to detect crystal violet (CV) molecules of concentrations as low as 10^−10^ M. In addition, Rao et al. showed that modification of Cu substrate with Ag is highly dependent on the concentration of Ag [178]. The maximum spectral enhancement is produced only for a 12 mM concentration of Ag particles, with higher concentrations leading to a decrease in signal.

In addition to high sensitivity and stability, novel types of copper mixed with noble metals-based SERS substrates have demonstrated potential biosensing applications in the detection of tumor and cancer cells. In a more recent study, Wen et al. fabricated porous CuFeSe_2_/Au heterostructured nanospheres by photoreduction method, which was applied for lung cancer cell detection [226]. The nanospheres were prepared by loading gold shell onto CuFeSe_2_ nanocrystals (NCs) surface under near-infrared (NIR) irradiation (λ > 850 nm). The prepared substrate did not only show high sensitivity (1.0 ppb) and selectivity, but also excellent photocatalytic cleaning performance by photodegrading undesired absorbed biomolecules. This property may be valuable for biomedical and bioanalytical assays.

As it was mentioned in the previous section, surface oxidation remains one of the limiting factors for copper substrates to be widely used in plasmonic applications. However, with the introduction of nanostructured noble metals such as silver and gold, this problem can be eliminated. Owing to the protective and enhancing properties of silver and gold, respectively, copper-based substrates with nanostructured silver and gold seem to be more attractive plasmonic material in SERS measurements compared with pure copper.

## 4. Silicon

Silicon was first proposed as a substrate for surface-enhanced spectroscopy in a patent application in 2002 by Chan et al. [227]. In this patent, the thin layer of the Raman active metal as silver or gold was used for coating the porous silicon layer, instead of the glass or other flat substrate that had been standardly applied. The porous silicon may be formed by the anodic etching in HF. This is carried out because the porous material is more convenient for the nucleation and growth of the metal nanoparticles. Furthermore, with an increase in the porosity of the silicon layer, the intensity of the peaks in Raman spectroscopy also increases. It was recommended by the creators to use silver or gold nanoparticles for coating the porous silicon to further increase the enhancement of the signals. The detection of a single-molecule or picomolar concentration of the adenine was achieved by coating the silicon layer with silver nanoparticles [227]. After the establishment of this method, in the following years silver-plated porous silicon could detect 1 nM of adenine in 2004 [228]; silver-modified silicon nanowires were used for the detection of 1 μM of R6G in 2006 [229]. Later, other metals were applied together with the silicon: platinum nanoaggregates on the silicon wafers could provide the enhancement factor of 50,000 for the detection of R6G, in the study conducted by Nam Hoon Kim in 2005 [138].

In the last 5–6 years numerous research on Si-based materials has been carried out and the variability of the substrates has also vastly increased. In the following review paper, the list of discussed substrates includes porous, mesoporous, pyramidical Si layers, Si nanowires, Si nanorods, SiO_2_ spheres, Si nanoparticles, black Si with Au, Ag, Cu, Pt, Pd, graphene, Al_2_O_3,_ Fe_3_O_4_ and ZnO. Overall, 82 different research works regarding the SERS measurements of Si-based substrates are overviewed, 8 of which are clinical studies. The usage of pure Si as the substrate is quite rare and only 5 analytical studies that belong to this category are presented in this review [65,66,67,68,69,70]. However, coating the Si surface with the nanoparticles of noble metals such as Au, Ag, Pt, and Pd or with other different components is quite common practice and 73 analytical methods discussed in this paper can be proof of this claim. The SERS performance of the substrates in the analytical studies was assessed in accordance with their enhancement factor and the limit of detection, while for the examination of the clinical performance the sensitivity, specificity, and accuracy in the diagnosis were measured. The results of the analytical studies can be found in Table 1, while the clinical research results are present in Table 3. It should be noted that some research works contain data about both the analytical and clinical performance of the substrate and consequently were included in both categories.

### 4.1. Two Groups of Si Comparison

After the comparison of pure silicon substrates with multi-elemental substrates containing the silicon, it was identified that the latter tends to provide better SERS results. Due to the lack of methods in the pure silicon group, the average EF and LOD may not accurately describe SERS performance of pure silicon. The geometric average of EF for the pure Si and multi-component Si substrates are 2.6 × 10^5^ and 1.4 × 10^7^, which implies that the metal-coated Si provides by 2 orders of magnitude greater enhancement than the pure silicon substrates. The highest EF for the metal-coated Si group is 10^15^ for AgNP@PMMA@Si substrate or silver nanoparticles @ polymethyl methacrylate nanocomposites [122], while the best EF for pure Si belongs to SiO_2_ nanowires and is equal to 4 × 10^7^ [67]. A similar trend was observed for limit of detection (LOD), metal-coated Si achieving by 2 orders of magnitude lower LOD (1.6 × 10^−11^ M) than the pure silicon substrates (4.9 × 10^−9^ M). In 2009, Galopin et al. also reported a comparable conclusion, where pure Si nanowires could provide the detection of 10^−4^ M of R6G, while AgNP-coated Si nanowires showed significantly better SERS performance with EF of 2.3 × 10^8^ and LOD of 10^−14^ M for the detection of the same probe [66].

### 4.2. Overall Si and Ag/Au Discussion

After comparing the pure and multi-elemental silicon-based substrates, these groups of substrates were combined into one to determine the effectiveness of Si-based substrates on SERS measurements in comparison to conventional SERS substrates such as Au and Ag. For this purpose, EF and LOD of 8 SERS studies applying Au and Ag separately or together as an alloy were used. The geometric average of EF was measured to be about 6.4 × 10^7^ for Au/Ag substrates, which is slightly higher than the silicon-based substrates that have an average EF of 1.1 × 10^7^. However, the highest EF of Au/Ag was reported to be about 10^10^ for the silver/gold substrate which is smaller by five orders of magnitude than silver nanoparticles coating the silicon surface (EF of 10^15^) [122]. Regarding the LOD of the conventional SERS substrates, the detection of a single-molecule by the usage of the AgNP was possible as early as in 1997 [19]. Since then, a few studies using Ag and Au reported the possibility of analyzing a single-molecule [192,197]. The single-molecule detection of adenine was reported in the patent application for the first Si-based substrate by Chan et al. [227]. On average Si-based substrates could detect down to 2.3 × 10^−11^ M, which was only about two times worse than pure Au and Ag substrates (average LOD 1.04 × 10^−11^ M). Nevertheless, from the LOD of the Si-based substrates, the detection of the trace and even single molecule of the analyte seems possible. This is because LOD of 7.8 aM for the detection of miRNAs obtained with the glucose@3-MPBA@AuNF@Si wafer should enable the detection of the trace amounts of the analyte. In addition, this biosensor demonstrated good uniformity with the relative standard deviation (RSD) of 0.53% for 20 points in one sample and RSD of 0.59% for 6 different substrates. When the following substrate was used to detect miRNAs in the 100-fold diluted human serum samples good recoveries from 96.13% to 103.4% were obtained [108]. Therefore, Silicon-based substrates can be a good alternative to the SERS active metals such as Au and Ag in the analyte detection.

Si-based substrates enriched with noble metals are not only efficient in comparison to the pure Si substrates but also might be better than pure metal substrates. Kunushpayeva et al. reported that when four parametric logistic curves are applied for the calibration in sandwich immunoassay of human IgG, LOD for assay on Si would become remarkably lower than LOD for the assay on Au (3 pM vs. 28 pM) [50]. This could indicate that silicon is a very promising SERS substrate. Nguyen et al. have conducted a study to compare the SERS performance of pure silver nanocubes and silver nanocubes coated with SiO_2_ shells of different thicknesses. As a result, it was found that AgNC with the 1.5 nm layer of SiO_2_ shells gives two times higher enhancement factors than AgNC on its own, being 1.3 × 10^6^ and 6 × 10^5^ correspondingly. Another outcome of the following study is an increase in EF caused by the decrease in the thickness of the silica shell (4.4 nm SiO_2_ shell coating AgNC provided an EF of 3.8 × 10^5^) [106]. A similar trend with the rise of EF by coating with the silica layer was obtained from two SERS studies performed by Liu Yu-Chan in 2009. Silver and gold nanoparticles coated with SiO_2_ and silica, respectively, provided 3-fold increase in EF compared to bare silver and gold [112,113]. The comparative SERS measurements in the study of Wang et al. imply that the Ag-NPs@Al_2_O_3_@Ag@Si array exhibit higher SERS activity (EF of 10^9^ and LOD of 1 pM) than AgNPs@Al_2_O_3_@Ag film (without Si nanocones), which is mainly ascribed to the higher density hot spots caused by the larger specific surface area of the silicon nanocones [75].

The substrate with the highest EF presented in this review achieved the enhancement (10^15^) by producing the SERS platforms with the controlled shape, size, and most important the uniform inter gaps between the silver nanoparticles. To prepare the substrate, incompatible solutions such as PMMA/acetone and AgNO_3_/ethanol were mixed and poured into the silicon wafer. Next, two types of micelles of different sizes which were Ag+, ethanol and acetone were spread on the Si substrate. Later after the evaporation of the alcohols, nanoholes with two different types of diameters on the PMMA surface containing AgNP were left. The N-doped silicon substrate was also highly useful to promote the electrons to the charged silver to reduce and obtain silver nanoparticles. After performing these steps, AgNP embedded in a thin hydrophobic PMMA layer on the silicon substrate was obtained. shape, and interparticle gap distances [122].

The electrodeposited arrays of thorny Au nanostructures on the silicon wafer with highly roughed surfaces demonstrated EF = 1.9 × 10^7^ and LOD = 0.45 pM for R6G detection, while the ferbam fungicide was detected down to 1 ppb. The following substrate was also denoted as recyclable due to the generation of similar SERS spectra for 10^−6^ M of R6G after cleaning the substrate repeatedly three times. Not only the reusability but also the reproducibility of the AuNS@Si substrate showed promising results: 100 different spectra from different locations on the same substrate had RSD of 6.6%, whereas substrate-to-substrate reproducibility was calculated to be 11% for five substrates [230]. Numerous other studies demonstrated good spot-to-spot or substrate-to-substrate reproducibility with RSD ≤ 10% by using Si-based substrates [73,74,80,81,85,102,115,117,119,129,231].

SERS silver substrates tend to encounter the issue of stability upon exposure to the atmosphere, due to oxidation. According to Tegegne et al., the SERS signal of 1 μM of R6G molecule measured on AgNCs@ polymethylhydrosiloxane (PMHS) modified filter paper declined by 78% after 90 days of exposure to air. The same substrate after the modification with SiO_2_ showed improved stability since the SERS signal after 3 months on airdropped by 26% only [193]. Similar findings were obtained by graphene-coated Ag nanostructures on the surface of the laser-textured Si, after 50 days of exposure of the substrate and R6G analyte to the ambient air, the SERS signal decreased by about 23% [128]. Graphene-coated Si nanowires also exhibited good stability by providing identical Raman intensities of the analyte after 30 days on air [119]. Wang et al. reported that due to the protection of the alumina dielectric layer and the organic solvent for AgNPs and Ag@Si nanocones, almost no change in the signal was observed in the first five months and only a slight drop was detected after 1 year, while the signal from the bare silver nanoparticle substrates tends to drastically decrease on air because of the oxidation [75].

Substrates with silicon can also be successfully applied for SERS detection of the various analytes. For instance, Ag and Pd alloy coated on the gradient porosity Si layer could detect as low as 1 CFU/mL (colony-forming unit per milliliter) of *E. coli* [99]. Moreover, down to 100 cells/mL of *E. coli* O157:H7 could be determined by silica core coated by thin gold shell coupled lateral flow immunoassay [109]. SERS immunoassay was applied in other studies as well to detect different cancer biomarkers, as a result LOD for Prostate Specific Antigen (PSA): 1.79 fg/mL; α-fetoprotein: 0.46 fg/mL by SiNP@AgNP@SiC substrate [95]; ferritin antigen 0.32 fg/mL by AuNP@SMSiO_2_ [107]; PSA: 0.46 fg/mL, prostate-specific membrane antigen (PSMA): 1.05 fg/mL, human kallikrein 2: 0.67 fg/mL by SiC@Ag(film)@AgNPs [130] were obtained. This demonstrates the potential of Si-based substrates in clinical diagnosis. Furthermore, Si-based SERS substrates can be applied in agriculture and food safety for the detection of pesticides. The detectable concentration of the thiram (pesticide) achieved by AuNP@Si nanowire paper was 72 ng/cm^2^, while the permitted concentration was about 2 μg/cm^2^. It implies that the following substrate can be used to assess the safety of fruits by measuring the quantity of the pesticides [71]. Imidacloprid is an insecticide that was recently banned due to its lethal impact on pollinators. Considerably inexpensive SERS technique for the detection of imidacloprid was suggested by Al-Syadi et al., who applied mesoporous silicon-plated Pd nanoparticles as the substrate and achieved the detection down to 1 nM along with EF of 10^5^ [91]. Qiu et al. applied silicon both as the SERS-active substrate in the graphene-coated AgNP on the Si and as the solid phase microextraction fiber to perform the in situ detection of Bisphenol-A. Therefore, Si fiber in the substrate immersed in the water extracted BPA and provided LOD of 1 μg/L. Further, the following method was tested for uniformity and reproducibility and the obtained RSD were 14% and 13%, correspondingly. The recoveries from 97% to 110% were derived from the spiked samples of BPA in water [118]. The optical biosensing approach for bovine mastitis (inflammatory disease condition of the bovine mammary gland with a significant economic impact on the dairy industry) severity evaluation using Ag on porous Si SERS platforms was carried out by Nirala et al. [90]. The SERS signal generated by the biochemical reaction products is correlated to the N-acetyl-β-D-glucosaminidase (NAGase) activity, the biomarker of bovine mastitis. The SERS biosensing platform had recovery values of 85–98% in comparison to the conventional fluorescence technique, while LOD was 8.1 μM and 2.0 μM, respectively. Since the SERS method can differentiate between healthy, subclinical, and clinical bovine mastitis, it can be considered as an alternative to the conventional fluorescence method [90]. Thus, Si-based SERS substrates can be efficiently applied in ecology, food control, agriculture, biosensing, and clinical diagnosis.

Even when Si is not combined with other metals and nanomaterials, different types of substrates can be produced: silicon nanowires [66], silicon dioxide nanowires [67], Si@SiO_2_ quantum probes [70], laser-irradiated Si [68], black Si [65], and silicon nanoweb structures [69]. This silicon nanoweb material is comprised of an interconnected network of hybrid amorphous/crystalline nano spheroids produced by using the 1030 nm pulsed Yb-doped femtosecond laser to texture the crystalline silicon surface. These individual nano spheroids with nanogaps of 5.5 nm are the reason for the enhancement of the Raman signal. The SERS activity of these Si nanoweb structures was compared with the bare Si surface. The characteristic peaks of R6G and CV dye are well-defined, and the intensity of the signals is considerably higher when Si nanoweb substrates are applied than the bare Si wafer.

### 4.3. Application of Silicon as a Substrate for a Sandwich SERS Immunoassay

**Figure 1:**
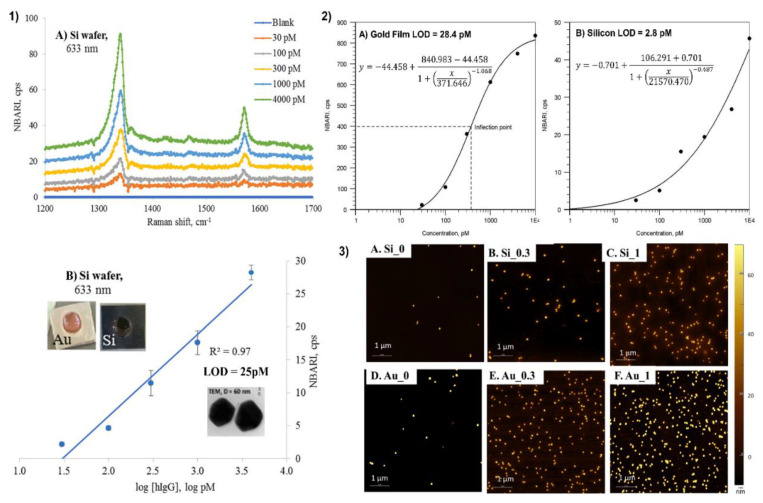
(**1**) SERS spectrum (**A**) and below calibration plot with the pictures of the substrates (**B**) for the sandwich immunoassay of human immunoglobulin (hIgG) on Si substrates obtained by using a 633 nm laser. NBARI—normalized blank adjusted Raman intensity from 3 different sets of measurements carried out on different weeks. (**2**) Calibration curves obtained by 4 parameters logistic non-linear regression analysis for Au and Si substrates on the first week. (**3**) Representative AFM maps of increasing hIgG concentration: 0 (blank), 0.3 nM, 1 nM for A, B, C—silicon; D, E, F—gold, respectively. Reproduced from Kunushpayeva et al. under the Creative Commons CC BY license.

**Figure 2:**
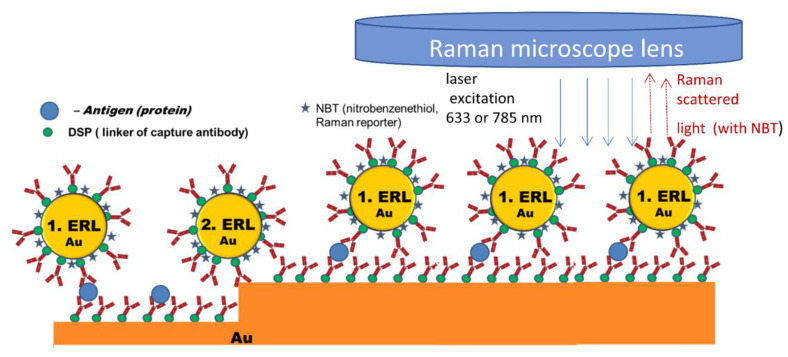
Scheme of specific (1. ERL or extrinsic Raman label) and non-specific (2. ERL) binding of Extrinsic Raman Labels (ERLs) to the capture antibody covered gold film substrate for SERS sandwich immunoassay. The detected cancer marker is an antigen. Non-specific binding to Si and even Al must be much weaker that non-specific binding to gold.

A comparative sandwich SERS immunoassay on gold and silicon surface using human immunoglobulin (hIgG) was performed by Kunushpayeva et al. [50] As demonstrated in Figure 1(1) with a rise in the antigen’s concentration, the intensity of the Raman signal increased for the silicon substrate, and a similar trend was observed for the gold as well. On average, the Raman signals on gold were at least by one order of magnitude higher in comparison to Si. Figure 1(1) displays Raman spectra and the calibration plot for five data points with different human immunoglobulin (hIgG) concentrations (from 30 to 4000 pM) for Si using a 633 nm laser. The correlation coefficient (R^2^) of the linear logarithmic trend was equal to 0.97 on Si, while the R^2^ value on the Au substrate was 0.95. The AFM maps of blank (0), 300 and 1000 pM hIgG samples for Si and Au substrates are presented in Figure 1(3). The strong positive correlation between the antigen concentration and the number of nanoparticles per area can be noted from the figures. Raman signal per nanoparticle on Au was greater than on Si substrate by approximately one order of magnitude. Correspondingly, the blank signal on the gold substrate was also higher in comparison to the silicon substrate. Despite the slope of the signal vs. log [hIgG] on the calibration plot being higher for gold, the limit of detection on the silicon substrate was a bit lower: for instance, 25 pM on Si and 34 pM on Au, respectively. Those LODs correspond to the detection of 0.8 picomoles and 1.1 picomoles of the antigen sample, respectively. Furthermore, the limit of detection for the silicon was about one magnitude lower than for the gold, 3 pM, and 28 pM, while applying the four-parameter logistic model for calibration and the calculation of LOD, which is a common approach to build a calibration curve in such well-established assay technique as ELISA. The LOD on the silicon substrate was better since the standard deviation of the blank on silicon is significantly lower than the standard deviation of the blank on the gold surface. The LOD by definition is directly proportional to the standard deviation of the blank (×3) and inversely proportional to the slope of the calibration plot. Therefore, LOD is somewhat proportional to the relative standard deviation, since the slope is very roughly proportional to the signal. Indeed, on average there is not only a much lower absolute standard deviation in the signal for the assay on silicon, but even the relative standard deviations (RSD) for the Raman signal on silicon is about two times lower than RSD for the assay on the gold substrate. For instance, the average RSDs for two lasers (633 nm and 785 nm) for 30 pM of hIgG was 12.1% on Au and 5.5% on Si [50]. Silicon might have better RSD than gold due to its better selectivity for the sandwich immunoassays and lower non-specific binding. One kind of non-specific binding is illustrated in Figure 2, where AuNP modified with antibodies and Raman active marker (NBT) is shown to bind not to antigen as it should produce a specific response, but to a spot of bare gold film, which is not an ideally flat surface but has surface roughness as measured in Sergiienko et al. [39]. Non-specific binding of proteins creates likely a major challenge to assays’ selectivity and sensitivity in the presence of other (not-antigen) proteins [4]. Non-metallic substrates such as Si are likely to have lower contamination on the surface in comparison to gold, particularly with S-containing compounds. Moreover, non-metallic and even non-noble metal substrates are likely to have also less non-specific binding (including protein adsorption) on their surface in comparison to gold, which can significantly adsorb S-containing amino acids, abundant in all proteins used in the assay [4,232]. In addition, van der Waals interactions between proteins and silicon/aluminum would be significantly weaker than the interactions between proteins and gold, as demonstrated by a lower Hamaker constant for Al in comparison to gold [233]. Those considerations would also predict better selectivity for the sandwich immunoassays on silicon and aluminum. Currently, the comparative SERS sandwich immunoassays of biomarkers on aluminum substrates are in progress.

## 5. Aluminum

To the best of our knowledge, the first application of aluminum(Al) in the fabrication of SERS substrate was recorded by Gao et al. in 1985 [234]. They studied the effect of aluminum submonolayers on the surface-enhanced Raman scattering (SERS) of oxygen on silver (Ag) films. It was found that aluminum submonolayer coverage reduces the SERS of oxygen on the silver surface (e.g., the integral of the 1047 cm^−1^ oxygen peak is reduced by a factor of ten by 0.5 Å Al layer). Though the origin of this quenching was not determined exactly, these insights paved the way for further developments and investigations of aluminum as a potential SERS substrate. Later, the group of professors at Jung Sang Suh at Seoul National University observed the strong SERS effect of benzoic acid adsorbed on surfaces of silver particles deposited on anodic aluminum oxide (AAO) pores, whose oxide layers were partially removed after the deposition of silver [235]. The SERS spectrum of benzoic acid on the surface of Ag/alumina/aluminum was compared with the spectrum on the silver colloid surface. The two spectra had almost the same signal-to-noise ratio and shape, indicating Raman enhancing the properties of AAO and its prospective applications in the fabrication of SERS substrates. In the late 2000s and early 2010s, AAO templates attracted great attention due to the facile control of different configuration shapes, rigidity, and stability [158,160,162]. At the moment, the interest in aluminum as a plasmonic material for SERS measurements has significantly increased. Aluminum-based SERS substrates are fabricated by different techniques such as e-beam lithography [235], template stripping method [152], nanoimprint lithography [236], and electron beam evaporation [162]. The following paragraphs are focused on the advantages of aluminum relative to conventional silver and gold-based SERS substrates.

**Figure 3:**
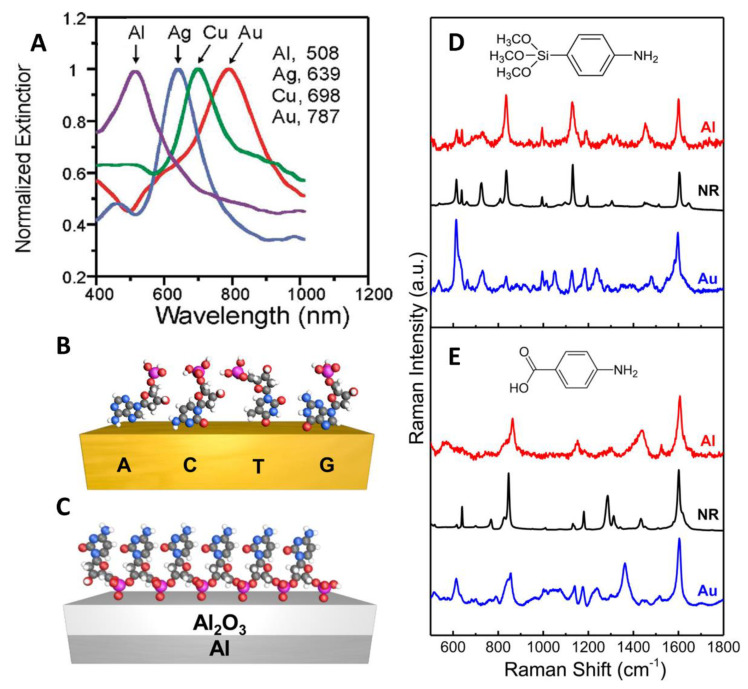
(**A**) LSPR spectra of nanoparticle arrays with identical geometries but varying metal (Al, Ag, Cu, or Au). Reprinted with permission from Chan et al. Copyright 2008 American Chemical Society. (**B**,**C**) Illustration of different surface binding mechanism of ssDNA molecules onto Au and Al substrates (**D**) SERS spectra of APhS and (**E**) PABA on Al (red) and Au (blue) substrates with normal Raman spectra (black) as reference. Reprinted with permission from Tian et al. Copyright 2017 American Chemical Society.

Aluminum is a promising plasmonic material due to its lower cost, great abundance on Earth’s crust, and inherent sustainability. Several studies of the plasmonic properties of Al demonstrated that Al nanoparticle arrays support localized surface plasmons in ultraviolet, visible, and near-infrared regions [154,205,237]. Figure 3A presents localized surface plasmon resonance (LSPR) spectra of aluminum, copper, silver, and gold nanoparticles of the same geometry (nanosphere diameter, D = 390 nm; deposited metal thickness, d_m_ = 50 nm; glass substrate, N_2_ environment), all fabricated by nanosphere lithography (NSL) method [238]. From the comparison of the LSPR behavior of Al, Cu, Au, and Ag, it can be concluded that Al nanoparticles show broader LSPR in the visible region. This property allows aluminum-based substrates to be used in broader spectral ranges compared to noble metals such as silver and gold. An example of this can be observed in the work of Sharma et al. where they compared Al and Ag-based substrates with deep UV wavelength excitation [156]. They found that the Al substrate produces 160× higher signal enhancement for adenine than the Ag substrate. As they were able to access higher excited levels which led to the excitation of different vibrational modes.

In addition, the inherent surface oxide layer of aluminum protects against corrosion and allows the binding of different types of functional groups, providing in this way a versatile platform for different molecule-substrate interactions [151]. For example, Tian et al. investigated the properties of Al nanocrystal (NC) aggregates as near-infrared SERS substrates and compared their surface chemistry with Au nanoparticle (NP) aggregates [151]. A distinguishing feature of AlNCs is their thin (2–4 nm) and stable oxide layer that precludes the further oxidation of the metal [239]. It is quite advantageous for SERS since this inherent surface oxide layer provides binding sites for different functional groups (e.g., carboxylic and phosphoric acids, silanes, and amides) other than thiol or amine groups usually found in noble metals. For comparison, Figure 3D illustrates SERS spectra of p-aminophenyltrimethoxysilane (APhS) on both Au nanoparticle (NP) and Al nanocrystal (NC) substrates. The peaks at 1132 and 838 cm^−1^ corresponding to Si-O-C stretching and Si-C rocking modes, appear more prominent on the aluminum spectrum than on the gold, indicating that the molecule of analyte binds to the Al oxide layer through the silane group more efficiently than in the case of the gold. In addition, the spectrum of p-APhS on the Au NP is more complex, due to the binding of analyte molecule to Au through the amine group, along with possible polymerization and hydrolysis of silane groups of p-APhS [151]. Another example shown in Figure 3E illustrates the SERS spectra of p-aminobenzoic acid (PABA). Here, PABA binds to the gold surface directly through the amine group (the peak at 1362 cm^−1^ characteristic of a C-N stretching mode enhanced due to the interaction of the nitrogen atom of the amine group with the Au NP surface), while in AlNCs the analyte molecule binds to the oxide surface via a carboxylic group (evident from the peak at 1439 cm^−1^ characteristic of a –COO^−^ symmetric stretching mode). All of these examples illustrate the point that the surface oxide layer of aluminum provides binding sites for a wider range of functional groups (e.g., carboxylic and phosphoric acids, silanes, and amides) other than thiol or amine moieties used in traditional Au and Ag substrates [153]. Such versatility of Al-based substrates can expand the selection of probe molecules for future applications.

For instance, the strong affinity of the phosphate group to the surface oxide layer of aluminum enables SERS-based detection of DNA [240]. Previous studies have shown that gold-based substrates exhibit poor reproducibility and large spectral variations due to different affinities of nucleic acids toward the gold surface [241,242]. This is because the binding is usually through covalent interactions of the noble metal substrate with the base ring of nitrogen and exocyclic amino/keto group, leading to the formation of randomly coiled configurations of ssDNA on the Au surface with multiple binding sites [151]. To avoid these types of nonspecific interactions, either the substrate or the molecule needs to be modified. However, further modification (e.g., modification of ssDNA chains with thiol group [243]) adds complexity to the detection process. With its native oxide layer, Al resolves this issue and enables label-free SERS-based detection of DNA. Figure 3B,C illustrate different binding mechanisms of ssDNA onto gold and aluminum-based SERS substrates. The peaks at 1096 and 786 cm^−1^ assigned to symmetric stretching and skeleton stretching vibrations of the phosphodioxy (PO_2^−^_) group indicate the interaction of phosphate groups of the ssDNA with the AlNC surface. The binding of ssDNA to the AlNC oxide surface through the phosphate backbone preserves the Raman features of bases, allowing reliable identification and study of DNA bases which is valuable in clinical diagnostics. On the contrary, on the Au surface, ssDNA binds predominantly through nitrogen-constituent groups, resulting in Raman shifts. As all of the previous examples demonstrate, the inherent surface oxide layer gives a comparative advantage to aluminum since it not only protects the substrate from corrosion but also provides a more versatile platform for various substrate-analyte interactions.

Another advantage of the aluminum substrate is its recyclability. This feature is essentially important in the detection of environmental pollutants. Gold-based substrates despite their excellent stability can be too expensive to meet the needs of environmental detection [244]. In the case of silver-based substrates, despite their lower cost and high SERS activity, they are prone to surface oxidation when exposed to air. In these regards, Al-based SERS substrates seem better alternatives for environmental detection. For example, Chen et al. developed the SERS substrate based on aluminum/graphitic-carbon nitride (Al/C_3_N_4_) loaded with various sizes of prismatic AgNPs for the cycled detection of environmental pollutants such as brilliant blue, crystal violet, and 4-aminotriophenol [245]. In comparison to the silver-based substrate, the Al/C_3_N_4_/Ag_x_ substrate exhibited a more uniform distribution of hot spots and thus more ordered distribution of target molecules. Moreover, the SERS spectra and relative intensities of brilliant blue on the Al/C_3_N_4_/Ag_168_ hardly changed for 45 days and after 7 recycles, indicating high reproducibility and continuous stability of the fabricated substrate. The limit of detection (LOD) of brilliant blue was also as low as 5.38 × 10^−11^ M, demonstrating high sensitivity in the detection of environmental pollutants. Another research carried out by Chang et al. illustrates the ease of recycling Al-based SERS substrates [161]. In their work, they tested the photocatalytic efficiency of Al-doped ZnO@SnO_2_ heteronanowires for the decomposition of rhodamine 6G (R6G) solution. The substrate was prepared using Al-doped ZnO nanowires via a simple hydrothermal method in an aqueous solution (50 mL) of tin dioxide (SnO_2_) reaction precursor of different concentrations (3, 6, 12, 24, 38 mM SnO_2_). The photocatalytic efficiency of the Al-doped ZnO@SnO_2_ (24 nM SnO_2_) substrate remained around 99% even after five cycles. Moreover, the Field Emission Scanning Electron Microscopy (FESEM) image and X-ray photoelectron spectroscopy (XPS) spectrum of recycled and fresh substrates have also been shown to be relatively the same, demonstrating high stability and reusability. The Al-doped ZnO@SnO_2_ heteronanowires can also be used for trace detection of drugs such as amoxicillin, an antibacterial drug used to treat bacterial infections in animals and humans [246]. The detection of amoxicillin was down to 10^−10^ M, by two orders of magnitude better result compared with the previous report 10^−10^ M [247]. One of the possible reasons for such a low limit of detection could be the optimization of shell-thicknesses of Al-doped ZnO@SnO_2_ heteronanowires, which allowed exhibiting a suitable geometry to deposit high-density Ag nanoparticles and hence generate more hot spots in three-dimensional structures. Choosing the right concentration of SnO_2_ precursor was also an important step in substrate preparation since it not only influences the shell thickness of SnO_2_ (shell thickness of SnO_2_ increased with the concentration of SnO_2_ precursor), but also the number of oxygen vacancies. The oxygen vacancies acted as positive charge regions to trap electrons, enhancing the photocatalytic efficiency of the substrate by separating electron-hole pairs. The appropriate amount of oxygen vacancies influenced the absorption properties of the substrate and its photocatalytic performance.

### 5.1. Limitations and Disadvantages of Al-Based Substrates

In the previous paragraphs, we pointed out several advantages of using aluminum in the fabrication process of SERS substrates. Those include resistance to corrosion, a broader range of analyzed molecules, and recyclability. We have also demonstrated that with the introduction of aluminum, the substrates can be used in the trace detection of clinically important drugs (ex. Amoxicillin) and biomolecules (ex. DNA). However, despite its numerous advantages, there are several limitations to aluminum that precludes the first from being as widely applied as gold or silver. First and the most obvious limitation of Al is its lower sensitivity compared with conventional SERS metals. From Table 1, SERS substrates based on pure aluminum have by 2–3 orders of magnitude lower enhancement factor (EF) and limit of detection (LOD) relative to pure gold and silver-based substrates. The sensitivity of Al-based substrates can be improved by several methods. For instance, using higher excitation powers, longer integration times, averaging more scans, and optimizing the geometry of the substrate [151,152]. In the case of Al nanocrystals (NCs), which were mentioned earlier, their size could be optimized to blueshift away from the energy of Al interband transition to increase the intensity of hot spots and improve the SERS signal at a more optimal excitation wavelength.

Table 3 summarizes the results of analytical studies from the years 2012 to 2020 of different types of analyte molecules (e.g., crystal violet, adenine, rhodamine 6G, NAP (naphthalene)) on pure Al-based substrates (e.g., Al NP-film, Al-coated inverted pyramid arrays, Al nanocrystals nanodots, nanovoids, Al bow-tie nanoantenna). On average, various Al-based SERS substrates without the addition of silver and gold are capable of producing surface enhancement of about 5 orders of magnitude (EF 10^5^) and ~10^−7^ M limit of detection (LOD) (Refer to Table 1).

### 5.2. Aluminum with Nanostructured Ag and Au

To analyze the impact of silver and gold addition on surface enhancement and the limit of detection of Al-based SERS substrates, we analyzed 9 analytical papers in total separately for each type of substrate (Al, Al + Au, Al + Ag) where R6G was used as a single probe molecule (See Table 2). In terms of surface enhancement silver and gold have approximately the same effect; EF in both cases equals ~10^7^. In the case of LOD, Ag had a better effect than Au. With the addition of Ag, LOD was 10^−11^ M which was 10 times lower than that of Au (10^−10^ M). Overall, these results demonstrate that silver and gold have approximately the same surface enhancement effect on Al-based SERS substrates, while LOD is slightly better with an addition of Ag than that Au.

Several studies demonstrate that the synergistic effects of Al and Ag can produce high EF and low LOD. For example, Das et al. fabricated Ag-capped Al nanorods by glancing angle deposition (GLAD) technique [164]. Compared with Ag nanorods (NRs), 50 nm Ag nanocaps on Al nanorods (NRs) exhibit significant surface enhancement up to the order of 10^7^ and a low limit of detection down to 10^−15^ molar concentration. The role of Al in the fabrication process is that it is cheaper than silver and gold and so Al NRs of larger sizes and lengths can be fabricated. Moreover, Al absorbs Ag nanolayer easily, resulting in uniform caps-like nanostructure throughout the SERS substrate and significant Raman enhancement. This type of low-cost, effective SERS substrate can be used for the onsite detection of clinical pathogens. For example, the developed substrate detected E. coli bacteria with concentrations varying from 10^8^ colony-forming units per mL (CFU mL^−1^) up to 10^2^ CFU mL^−1^.

Another study by Shan et al. showed that by optimizing the thickness of both deposited Ag and AAO membrane in an AAO/Al-based Ag nanostructure array, it is possible to achieve the SERS enhancement ~10^8^ which is higher in comparison with AAO-based Ag nanostructure array [162]. Overall, with the introduction of Al, the substrate not only has higher EF but also is more robust and easier to fabricate, which are important parameters to consider in practical applications.

### 5.3. Application of Aluminum Foil for Biosensing

**Figure 4:**
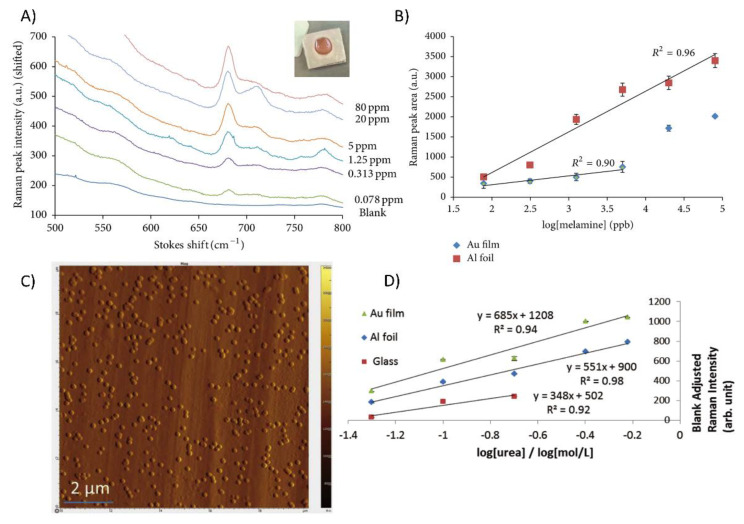
(**A**) Raman spectra of melamine (0.078–80 ppm concentration range) on commercial gold nanoparticles on Al foil (AuNPs@AlF) measured at 785 nm excitation wavelength (**B**) Calibration plot of 820 cm−^1^ characteristic vibration peak area vs. logarithm of melamine concentration in ppb on gold film and Al foil. Reprinted from Gudun et al. under Creative Commons Attribution License. (**C**) AFM map of 10 × 10 micron of 80 nm commercial AuNPs@AlF substrate (**D**) The plot of blank adjusted Raman intensity vs. logarithm of urea concentration (log[urea]) on three different substrates: AuNPs@Au film, AuNPs@AlF, AuNPs@glass. Reprinted from Mukanova et al. Copyright © 2018 The Japan Society for Analytical Chemistry.

In the work of Gudun et al., they fabricated a relatively low-cost, tunable, hybrid SERS substrate using commercial gold nanoparticles drop-casted on untreated Al foil (AuNPs@AlF) [36]. The limits of detection of 4-nitrobenzenethiol (4-NBT) and crystal violet were 0.12 nM and 0.19 nM, respectively, while the maximum analytical enhancement factor (AEF) was about 10^7^. They also detected KNO_3_ with LOD 0.7 mM and EF ~10^3^, which was about the same order of magnitude on commercial gold Klarite substrate [248]. Using 60 nm gold nanoparticles drop-casted on Al foil versus gold film (AuNPs@AlF vs. AuNPs@Au film), Gudun et al. detected melamine in an aqueous solution [36]. The Raman spectra of melamine at different concentrations are illustrated in Figure 4A. Though the LOD of melamine was about 7 times lower on gold film compared to Al foil (4 ppb vs. 28 ppb), the linear range of the assay on Al foil was significantly wider (1280 for Al foil, 64 for Au film) and the linear response was better (R^2^ 0.96 on Al foil, 0.90 on Au film) in comparison to gold film (Figure 4B).

The obtained SERS limit of detection on both substrates was 1.5–2.5 orders of magnitude below the tolerance level (1 ppm) for melamine suggested by the World Health Organization [249]. The same substrate (AuNPs@AlF) was used later on by Mukanova et al. for the detection of paracetamol in water and urea in artificial urine [37]. The paracetamol was detected at concentrations as low as 0.11 mM, which is about 9 times lower/better LOD compared to the previous work by Santos et al. on AuNP-chitosan films [250]. The limit of quantification of urea obtained on AuNPs@Au film was about 30% better than on AuNPs@Al foil substrate (18 mM and 26 mM, respectively), but still, SERS substrate based on Al foil detected urea in urine within the physiological range, below the lowest pathophysiological concentration of urea of 0.03 mM [37]. The key factor for the successful performance of the SERS substrate in these experiments was the optimal number of centrifugation/resuspension cycles in the substrate preparation step. Previously reported by Gudun et al., 3 centrifugations in the case of SERS detection of NBT produce 2 orders of magnitude better LOD (0.1 nM vs. 30 nM) than 1 centrifugation [36]. Overall, 2–3 centrifugation cycles produce the best SERS response. The possible reason for this phenomenon is that after each centrifugation/resuspension cycle, the loss of surfactant leads to a decrease in distance between particles, thus “hot spots” between gold nanoparticles become “hotter” and more accessible for the absorption of the analyte. However, a higher (4+) number of centrifugation/resuspension cycles may lead to strong agglomeration of nanoparticles, which would complicate or even prevent their resuspension [36].

## 6. Other Metals and Alloys

As it was illustrated in the previous sections, SERS substrates are generally made of noble metals (e.g., Au, Ag, Cu) due to their high plasmonic efficiency [158,160,162,164,167,168,169,176,207,234,235]. However, the costly preparation and disposable issues of these substrates obstruct the universality of SERS measurements [189]. To address these issues, numerous studies were focused on the fabrication of cost-effective and recycled SERS substrates for practical use. For example, the combined effect of TiO_2_/ZnO nanocomposites and noble metal present immense optical enhancement and photocatalytic degradation, which allows them to simultaneously detect and degrade target molecules, making them self-cleaning and recyclable SERS platforms [186,251].

Ma et al. demonstrated that Ag nanorods (NRs) coated by ultrathin and uniform HfO_2_ layer exhibited high SERS sensitivity and high-temperature robustness [189]. As it was able to continuously detect vapor phase analytes at low concentrations throughout 30 “detection-heating” cycles. This Ag NRs@HfO2 SERS platform can be used for SERS determination in aqueous solutions as well as in real-time identification of vapor phase samples such as air pollutants at ultralow concentrations. ZnO has also been used as a potential candidate for SERS substrate because of its high refractive index which can promote strong light confinement and, respectively, contribute to the increase of the SERS effect [184]. For example, Xu et al. achieved a 3-fold higher SERS signal with a superhydrophobic substrate based on a silver nanoparticle coated zinc oxide nanorods array (Ag@ZnO) than that on the ordinary hydrophilic Ag@ZnO substrate due to the superhydrophobic condensation effect [252]. Another research carried out by Shi et al. fabricated multilayer core−shell-nanostructured ZrO_2_@Ag@SiO_2_ nanoparticles through liquid extraction for the detection of 4-aminothiophenol (4-ATP) and rhodamine 6G (R6G) at 785 nm excitation and demonstrated that zirconia film protects the silver nanoparticles from aggregation, improving the stability of the substrate [190]. The list of 11 studies for SERS non-noble metal substrates other than Al and Cu (e.g., Fe_3_O_4_ @Au nanoshells, CoFe_2_O_4_@HNTs/AuNPs, Au-ZnO NRs, Au semishells on TiO_2_ spheres, etc.) are summarized in Table 4 [182,183,185,186,187,188,189,190,191,252].

Among those studies, several prominent works which achieved significant enhancement factor and limit of detection should be noted. For example, the Au-coated ZnO nanorods were capable of detecting methylene blue (MB) at picomolar levels with good reproducibility [184]. After ten adsorption/UV-cleaning cycles, the substrate gave reproducible results and kept full SERS activity upon storage for several months in the air. Another interesting research was carried out by Shao et al. [185]. They prepared a metal-organic framework (MOF) based MIL-101(Cr) film substrate. To be more specific, they synthesized a layer of MIL-101(Cr) film on rough titanium oxide foil by a secondary growth method, and then coated the surface of the film with AgNPs by partial reduction of silver ions. Recently, metal-organic frameworks (MOFs), a porous material with periodic reticular structures [185] formed by organic ligands and metal ions are gaining great interest due to their large surface area [253], high porosity [254], and tunable pore size [255]. In this work, the fabricated MOF-based SERS substrate was able to detect 4-ATP up to 10^−10^ M with a relative standard deviation (RSD) of 5%, which is a proof of good SERS performance and reproducibility. The substrate was also used to detect nitrofurantoin (NFT), a well-known antimicrobial agent, down to 10^−7^ M. Finally, Filippin et al. detected rhodamine 6G in water to picomolar levels using TiO2 nanotubes decorated with AgNPs as SERS active substrate [191]. They reported SERS enhancement up to ~10^8^ and they also suggested that the newly synthesized SERS substrate can be applied for the detection of different biomolecules (e.g., biomarkers of cancer) and organic pollutants (e.g., formaldehyde).

For the past few years, Au/Ag-coated Fe_3_O_4_ magnetic nanoparticles (Fe_3_O_4_@Ag or Fe_3_O_4_@Au MNPs) have attracted great interest for their versatility, complex sample detection, and stability [256]. The new type of Fe_3_O_4_@Ag magnetic tags conjugated with dual-layer Raman dye can recognize target molecules from a real biological sample without any pre-treatment steps partially due to the stability and enrichment ability of magnetic nanoparticles (MNPs) [257]. The role of the dye (5,5-dithiobis-(2-nitrobenzoic acid) (DTNB)) in the magnetic tags is to produce a strong Raman signal. Wang et al. used all of these properties to detect influenza A H1N1 virus and human adenovirus (HAdV), two well-known respiratory viruses. The limit of detection (LOD) for H1N1 and HadV were 50 and 10 pfu/mL, respectively, which were 2000 times better than the standard colloidal gold strip technique. By changing specific antibodies, it is also possible to detect other analytes such as biomarkers, bacterial pathogens, and toxins.

## 7. Performance Comparison with Gold and Silver Substrates

### 7.1. R6G (Rhodamine 6G) Detection by SERS

Even though the average results for each category of the substrates were described above, the EF and LOD of the same substrate can considerably deviate depending on the probe and the measurement conditions. For this purpose, studies using probably the most popular SERS probe molecule R6G as the analyte were separately analyzed (See Table S5), including publications about SERS on gold and silver substrates [196,197,199,200,258,259,260,261,262,263,264,265]. The results of this analysis can be observed in Table 5. The geometric mean for the EF of Si, Pt/Pd, and Au/Ag categories were calculated to be 1.7 × 10^7^, 1.8 × 10^5^, and 5.5 × 10^8^. It can be easily noticed that the previous trend with the Au/Ag group providing the best enhancement followed by the silicon-based category is kept. However, in the previous table with different analytes, the enhancement delivered by Au/Ag were about 100 times greater than by the silicon substrates, this time the difference between the following categories is only about 3 times. The trend for the mean LOD of the Si, Pt/Pd groups also do not experience a noticeable change by selecting only the R6G probe for the calculation, 3.8 × 10^−11^ M and 10^−9^ M, respectively. The LOD for Au/Ag substrates is 6 × 10^−12^ M for the R6G probe. Overall, it can be concluded that there is not any important deviation in the average data caused by the selection of R6G as a probe.

### 7.2. TNT (2,4,6-Trinitrotoluene) Detection by SERS

2,4,6-Trinitrotoluene is one of the most applied explosive chemicals for military purposes, the extensive exposure of which to human skin tends to correlate with anemia or abnormal liver functions [134]. Therefore, there is a need to detect the trace amount of TNT in the soil and groundwater, and numerous SERS studies were investigated for this purpose. Silver nanoribbons synthesized from 2 ps laser pulses and 1200 mJ input pulse energy provided EF of 10 million and LOD of 25 nM in the detection of TNT. AuNP modified with cysteine substrates generated better SERS results, EF of $10^{9}$ and LOD of 2 pM, in particular [194]. Despite Si-based substrates demonstrating EF of about 6–7 orders of magnitude lower than Au and Ag substrates [98,134], the best discussed LOD of 1 picomolar TNT was achieved by AgNP@Si substrate. Moreover, portable AgNP decorated silicon SERS chip can be used with a hand-held 785 nm excitation Raman instrument to qualitatively analyze $∼10^{-8}$ M of TNT in the environmental samples. Thus, the Si-based SERS chip is a promising tool in the detection of explosives in practice due to the portability and good LOD of 1 pM [134].

### 7.3. Adenine Detection by SERS

Adenine is one of the building blocks of nucleic acids that is widely applied in biomedicine and agriculture. Numerous SERS studies have been conducted to detect this molecule and one of the best reported LOD was achieved by Tzeng and Lin who could detect down to 10 picomolar neutral adenine solutions with good reproducibility by using Ag@Cu@Si substrate. Further, 1 picomolar detection was achieved with the same substrate by taking SERS measurements of the aqueous adenine solution at a pH of 9 [266]. Other silicon-based substrates such as AgNC@SiO_2_ on a polymethylhydrosiloxane modified filter paper and AuNP@FePt@SiO_2_ demonstrated the enhancement by 6–7 orders of magnitude and LOD of about 1 nM [101,231]. By using Al nanoparticles on Al film enhancement of 4 × 10^5^ and detection of 1 μM of adenine were attained [154]. Silver nanoparticles deposited on an anodic aluminum oxide presented a comparable performance with a detection limit of less than 1 nanomolar, and higher EF of around 7 million and 200 million, respectively [267,268]. Silver nanoparticles on the filter paper with EF of 10^7^ had presented a LOD of 160 nM [269], which is considerably worse than the performance of the substrates discussed before, while the SERS study applying AgNP on a chitosan flake (biopolymer) could detect down to 12 picomolar concentration of the adenine [270]. By comparing the following studies, it can be assumed that Si and Al-based substrates can outperform the common SERS substrates.

## 8. SERS Clinical Applications

With the enhancement of the Raman signal up to 10 million times and the limit of detection of about 25 pM, Silicon can be regarded as a substrate with promising potential in clinical applications. However, despite the plurality of the analytical SERS measurement papers using Si-based substrates that provided promising SERS results, the number of clinical papers was limited. For this review paper, 35 studies were selected for analysis of the clinical application of the SERS substrates, among which 14 are silicon-based, 7 are aluminum-based, 7 are bare silver, and 7 gold applying papers, respectively. Table 6 compares the average clinal performance of different substrate groups and Table S11 contains information on each study. The comparison reveals that the silicon-based substrates provide the most reliable SERS results, with a sensitivity/specificity/accuracy of 96%/95%/94.4%. Comparable accuracy of 94.2% was achieved by using aluminum substrates. The Ag@Al substrates demonstrated a notably better performance than the whole Al group, with the sensitivity of 93%, specificity of 100%, and overall accuracy of about 96%. The conventional substrates (Au and Ag) have slightly lower accuracy of 93% and 94%. The information about the specificity, sensitivity, or accuracy of each substrate type is limited to just several reports so those comparisons have limited reliability.

One of the advantages of conventional substrates such as AuNP and AgNP is they can be applied to differentiate between the various disorders and still maintain their high reliability. As an example, nasopharyngeal and liver cancer, and healthy species (overall 75 samples) were distinguished with an accuracy of 90.7% in the study conducted by Yun et al. employing AgNP as a SERS substrate [271]. Fang Yaping achieved 81.2% accuracy in differentiating the 7 different types of cancer cells in 350 samples by using gold nanoparticles for the SERS measurements [272]. This means that Au and Ag substrates are accurate not only in simply detecting the analyte, but also demonstrate fairly accurate results in the multi-variate clinical analysis.

**Table 6:** Summary of SERS clinical applications with different substrates.

Substrates	Average Sensitivity	Average Specificity	Average Accuracy	N of Samples	References
Si	95.7%	95.1%	94.4%	All: 56 (17, 116)	[70,130,131,132,133,273,274,275,276,277,278,279,280,281]
Only Al	83%	83%	83.3%	All: 60 (30; 30).	[282]
Ag@Al	91.3% (85%; 98%)	99.5%	94.2% (91%; 98%)	All: 108 (28; 190).	[283,284,285,286,287,288,289]
Au	94.7% (80.7%; 100%)	95.5% (84.1%; 100%)	92.8% (81.2%; 100%)	All: 127 (18; 280).	[272,290,291,292,293,294,295]
Ag	93.0% (80.9%; 100%)	95.3% (87.5 %; 100%)	94.2% (84.1%; 100%)	All: 145 (75; 220).	[271,283,296,297,298,299,300]

Note: The sensitivity/specificity/accuracy of the overall substrate group is presented in the following way: average (min; max). The number of samples: All samples (positive “+”, negative “−”).

### 8.1. Prostate Cancer Clinical Diagnosis by SERS

Prostate cancer (PCa) is the second most frequent cancer and one of the leading causes of cancer-related deaths among men that can be diagnosed by an elevated level of the prostate-specific antigen (PSA) [301]. However, sometimes the increased amount of PSA can correlate with non-cancerous benign prostate hyperplasia (BPH), which is the enlargement of the prostate gland that is less severe for the health than PCa [302]. Different SERS substrates coupled with statistical analysis methods such as Principal component analysis (PCA), Partial least squares-discriminant analysis (PLS-Da), and Support-vector machine (SVM) can be used to accurately diagnose PCa. PCa and BPH classification by SERS study of PSA in serum on AgNP substrates was carried out by Chen Na et al., as a result, prostate cancer was detected with 94.2% accuracy in 120 samples [296]. In the comparable multi-variate immunoassay applying SiC@Ag(film)@AgNPs as a substrate to differentiate PCa from BPH and healthy samples, prostate cancer was determined with 70% accuracy, while benign prostate hyperplasia with 60%, and healthy samples with 75%, respectively [130]. However, due to the small sample size of 10 prostate cancer positive, 10 benign prostate hyperplasia (BPH), and 12 healthy samples, the following results have limited reliability [130]. Furthermore, aluminum foil coated with the silver colloid also demonstrated high reliability in classifying prostate cancer and BPH by providing 98% accuracy for 28 plasma samples [289]. In addition, aluminum-based SERS substrate presented a high accuracy of 98% in diagnosing prostate cancer among 93 positive and 68 negative serum samples [284].

### 8.2. Lung Cancer Diagnosis by SERS

Lung cancer is considered to be the cause of the most cancer-related death both among males and females worldwide resulting in the death of more than 1.8 million people in 2020 [301], this shows the significance of the early and accurate diagnosis of lung cancer. Qian Kun et al. presented a 100% accurate diagnosis of lung cancer by the saliva test [293]. In this assay, SERS measurements of 61 positive and 66 negative samples were taken on gold substrates and later analyzed using the SVM [293]. According to Zhang et al., pure AgNP substrates in the SERS analysis of the serum samples resulted in the issue with the repeatability and the stability of the signals, due to the maldistribution of the substrate [274]. Nevertheless, the problem was tackled by coating the mixture of AgNP and serum on the pyramidical Si surface, in this research work, lung cancer was diagnosed with the sensitivity/specificity/accuracy of 100%/90%/95% in 50 patients and 50 healthy serum samples from the PCA-LDA method [274].

### 8.3. SARS-CoV-2 Detection by SERS

**Figure 5:**
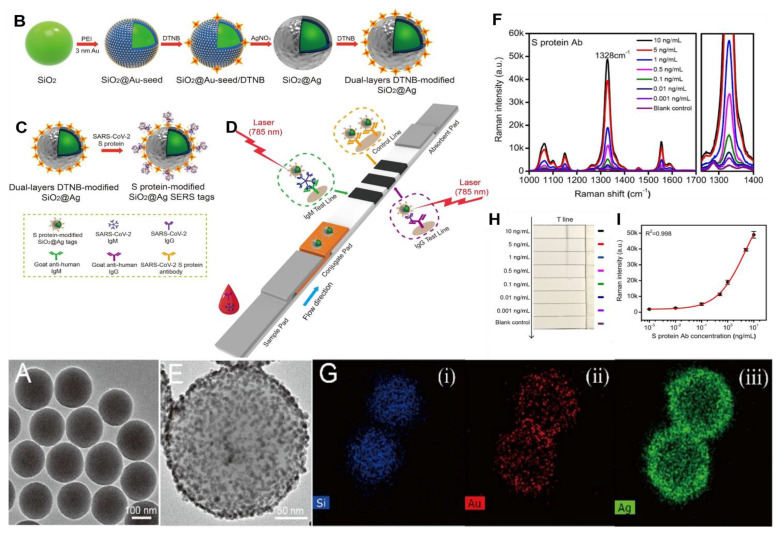
(**A**) HRTEM image of the prepared SiO_2_ Nanoparticles, (**B**) The preparation of the dual-layers DTNB-modified SiO_2_@Ag NPs. (**C**) The modification of the SiO_2_@Ag SERS tags with SARS-CoV-2 S protein. (**D**) Operating principle of the SERS-LFIA strip for the detection of the SARS-CoV-2 IgM/IgG. (**E**) Enlarged HRTEM image of SiO_2_@Au-seed NPs. (**F**) Raman spectra measured in the corresponding test lines and the enlarged viewport at the 1328 cm−^1^ characteristic peak. (**G**) Element mapping results of the SiO_2_@Ag NPs. (**H**) The images of the SERS-LFIA strips with a single Test line after application of the different S protein antibody concentrations (10–0.001 ng/mL). (**I**) The calibration curve of SiO_2_@Ag SERS-based LFIA for the S protein antibody. Reproduced with permission Liu et al. Copyright ©2021 Elsevier.

SARS-CoV-2 virus is the cause of the Coronavirus infection (COVID-19) that up to date resulted in more than 6 million deaths and about 627 million confirmed cases according to WHO (https://covid19.who.int/ accessed on 1 November 2022). Since the start of the pandemic, numerous studies based on different techniques including SERS have been conducted on the detection of SARS-CoV-2. Clustered regularly interspaced short palindromic repeats (CRISPR) coupled SERS method by Liang et al. introduced the amplification-free detection of SARS-CoV-2 RNA in 30–40 min of incubation by using silver nanoparticle substrates [299]. RNA extracts obtained from 24 infected and 88 healthy nasopharyngeal samples were classified with 87.5% sensitivity and 100% specificity, resulting in 97% accuracy of the following method [299]. The silicon-based substrate delivered better clinical results in the diagnosis of SARS-CoV-2 than AgNP. SERS lateral flow immunoassay (LFIA) biosensor employing Ag shell on SiO_2_ core substrate demonstrated 100% accuracy on 19 positive and 49 negative serum samples [273]. The visual demonstration of the assay can be found in Figure 5. The SERS tags were modified with SARS-CoV-2 S protein and three test lines designated for human IgG, IgM and control were present. When viral immunoglobulin is present in the blood, it would attach to the SERS tags in the conjugate pad and later be detected by the human immunoglobulins present on the test lines. The limit of detection was as low as 1 pg/mL which is about 2.2 fM (taking the molar mass of SARS-CoV-2 S protein as 455 kDa). Furthermore, according to the authors when the S protein concentration is above 1 ng/mL, the black bands can be easily detected by the naked eye. Another beneficial side of the assay was the quickness of the test because only about 25 min are required to obtain the results. In addition, the substrates can be considered stable, since even after 60 days of storage the detection performance of the SERS-LFIA method was not significantly worsened [273].

### 8.4. Clinical Diagnostics of Other Bio-Analytes on Si and Al-Based SERS Substrates

AgNP@Si substrate used in the research by Kaminska demonstrated 100% accuracy with PLS-Da analysis in the detection of Neisseria gonorrhoeae in male swab specimens and the differentiation from other bacterial pathogens such as Mycoplasma hominis, Mycoplasma genitalium, Ureaplasma urealyticum, and Haemophilus ducreyi [132]. 10 samples infected with gonorrhea or chlamydiosis and 10 healthy male urethra swabs were collected from which 600 spectra were obtained. 120 of spectra were used to test the following method, while the other spectra were used as a training set. Moreover, the following SERS study presented a good sensitivity for N. gonorrhoeae with the limit of detection as low as 100 colony forming units per mL. By the comparison of the bands’ characteristic to infected and healthy samples as shown in this study, sexually transmitted diseases can be diagnosed as fast as 15 min with high accuracy [132]. This demonstrates the promising potential of point-of-care SERS devices with Si-based substrates in the fast and reliable diagnosis of sexually transmitted diseases (STDs) and other illnesses.

100% accurate clinical diagnosis of breast cancer was achieved by Weng et al. for 60 samples [278]. In the following study, the targeted miRNA-21 and miRNA-155 were amplified by applying the isothermal catalytic hairpin assembly (CHA) strategy before SERS analysis. In addition, through the linkage between two-dimensional Au–Si substrate and upper Ag@4-MBA@Au core-shell nanoparticles, a sandwich SERS chip with numerous hot spots was built. The application of the signal second order peak of Si 936 cm^−1^ as an internal standard calibration enabled the reliable quantitative detection of miRNA with the correlation coefficient (R2) of 0.99. Thus, the ultralow concentration of miRNA-21 and miRNA-155 was detected (0.398 fM and 0.215 fM, respectively) and a 100% accurate diagnosis of breast cancer was performed [278].

Ma et al. compared the conventional AgNPs single-layer porous Si substrates with the AgNP porous silicon Bragg reflector SERS substrate, which was prepared by controlling the corrosion current. It was found that the enhancement coefficient of the Bragg reflector substrate is 3.2 times higher than the single-layer Si substrate. The X-ray diffraction analysis of the substrates demonstrated that the diffraction peaks of the multi-layer porous Si (Bragg reflector) became wider and wider, and the field intensity of the porous Si photonic crystals was stronger. The enhanced electric field on the surface of photonic crystals is effective in attaining a stronger LSPR that results in the enhancement of the Raman signal. As a result of using Bragg reflector porous Si substrate, breast cancer was diagnosed with 95% accuracy in 60 samples [275].

Electric field enhancements of the hybrid silica microsphere covered gold/silver nanoparticles and the pure metal nanoparticles (gold/silver) were investigated in the article by Wang et al. [277]. The interface of the plasmonic metal and dielectric spheres produces extra enhancement of neighboring electromagnetic field therefore both SiO_2_@Au and SiO_2_@Ag particles demonstrate substantial E-field enhancement in contrast to pure metal nanoparticles (AgNP and AuNP). It was also revealed that the hybrid silica sphere gold nanoparticles generate more enhancement of the signal than the silica-covered silver nanoparticles at 785 nm, while the silica-covered gold NPs produce higher signal at 532 nm This is because of the matched overlapping between AuNP LSPR in the hybrid particle and the excitation wavelength at 785 nm, whereas the electric field enhancement of SiO_2_@Ag particle is escalated around the LSPR of AgNP [277].

Metal-free SERS substrate approach using ∼a 2 nm layer of silicon dioxide on Quantum (Q)-probes for in situ live biosensing was attempted by Keshavarz et al. [70] Si@SiO_2_ Q-probes in contrast with Si Q-Probes displayed the further enhancement of the signal with the decrease in the dye concentration till reaching the limit of 5 pM, whereas the limit for Si Q-probes was about 1 mM. This phenomenon was hypothesized to be caused by the charge transfer mechanism from the semiconductor band edges to the affinity levels of the adsorbed molecule. The clinical diagnosis of HeLa cancer cells using Si@SiO_2_ substrate resulted in 86% sensitivity and 94% specificity [70].

**Figure 6:**
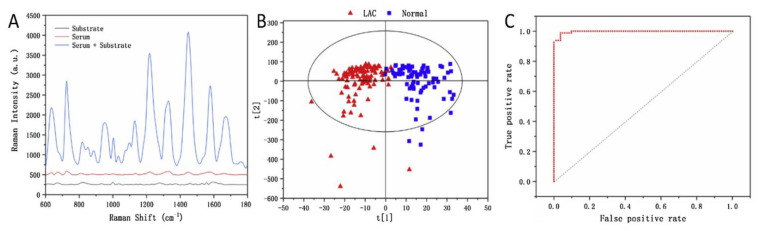
(**A**) Raman spectra of Ag NRs wrapped with Al_2_O_3_ layer, serum, and serum with the substrate. (**B**) Principal component score plots of the SERS spectrum based on OPLS-DA for LAC and normal samples. (**C**) ROC curve of LAC samples. Reproduced with permission from Liu et al. Copyright ©2019 Elsevier.

Even though the number of clinical studies on aluminum is limited if compared with silicon, in general aluminum demonstrated pretty good results in clinical studies that we reviewed so far [282,283,284,285,287,288,289]. For example, in the diagnosis of colorectal cancer (CRC), the third most common cause of cancer death worldwide (WHO 2020), aluminum foil alone proved to be not only cheap but also efficient at drying serum samples [284] and collecting the spectrum, along with being diagnostically effective when analyzing the fresh serum samples with sensitivity 83%, specificity 83% and accuracy 83.3% [282]. Several studies where AgNPs have been transferred onto an Al substrate (either Al plate or foil) also showed significantly good clinical results [284,285,287,288,289]. In addition, work by Liu et al. showed the application of aluminum oxide-modified silver nanorods on the diagnosis of lung adenocarcinoma with the sensitivity of 98.1% and specificity of 97.6% [286]. The comparison of spectra before and after atomic layer deposition of 1.5 nm Al_2_O_3_ layer on silver nanorods revealed a significant increase in Raman signal after aluminum oxide modification. This comparison and results of LDA analysis can be seen in Figure 6. Overall, from Table 6 Ag@Al substrates have demonstrated better clinical specificity (100% Ag@Al vs. 95.5% Au) than both silver and gold and relatively better accuracy (94.2% Ag@Al vs. 92.8% Au) than gold-based substrates. It’s worth mentioning that these values only give the general picture on how aluminum-based substrates perform relative to noble metals. There are several limitations in our calculation of average sensitivity/specificity/accuracy values. For example, we did not take into account the type of statistical algorithms applied in individual cases. Among studies that we reviewed, different multivariate statistical algorithms such as principal component analysis (PCA) and linear discriminate analysis (LDA), support vector machine (SVM), genetic algorithm (GA) combined with linear discriminate analysis (GA-LDA) techniques were used to distinguish different type of cancers from healthy individuals. In terms of performance, GA-LDA is better than the SVM algorithm, while SVM is better than PCA-LDA (GA-LDA>SVM>PCA-LDA), hence the difference between individual sensitivity/specificity/accuracy values was significantly different [287,288,289]. For example, Li et al. compared the performance of PCA-LDA algorithms to SVM in the classification of prostate cancer patients from healthy individuals [284]. The receiver operating characteristic curve (ROC) is a plot that demonstrates the performance of the classification model as a classification threshold is varied. The integration area under the ROC (AUC) of Gaussian radial basis function (RBF) kernel SVM and PCA-LDA were 0.998 and 0.991, respectively, in the work of Li et al. [284]. Generally, the AUC value correlates with diagnostic accuracy. The larger the AUC value, the greater the forecast accuracy for the classifier. From these results, the SVM algorithm exhibits a better accuracy (98.1% vs. 91.3%) than PCA-LDA. There are two possible reasons for the worse performance of the PCA-LDA algorithm. First, PCA-LDA can lose some important diagnostic information while processing the SERS spectra. Second, since PCA-LDA is a linear algorithm, it is unable to distinguish the nonlinear boundary between SERS spectra of prostate cancer and normal serum samples. Similarly, GA-LDA yielded a better diagnostic sensitivity of 90.9% and specificity of 100% than PCA (sensitivity 74.6%, specificity 97.2%) for classifying bladder cancer patients from healthy individuals [288].

In addition, due to different sample sizes, the classification accuracy of certain individual studies that we reviewed might not be reliable. For example, Zhao et al. achieved significantly higher accuracy 97.9% (28 serums samples) [289] than Li et al. 91.3% (161 serum samples) using the same PCA-LDA algorithm [284]. Herein, the lucky chance of getting a better result in a smaller sample-sized experiment is likely to be higher than with larger samples.

Finally, it’s worth mentioning that in clinical studies we reviewed so far, patients who submitted blood have been at different stages of cancer development. Thus, the results of statistical models that have been applied correspond to different cancer staging and may not be applied for the early detection of cancer as authors of individual studies claim. These and other factors have to be taken into account in our future works to improve the reliability of the reported results and present a clearer picture of the performance of non-noble substrates relative to traditional noble metals.

The clinical performance of the overall Si-based SERS substrates is a bit inferior in comparison to noble metals such as gold, silver, and silver on aluminum. Nevertheless, the silicon coated with noble metals as a substrate has demonstrated the best sensitivity/specificity/accuracy values (95.7%/95.1%/94.4%) and this illustrates the promising potential of the following substrate in clinical detection. The reason for such a claim is that there are already methods that demonstrated 100% accuracy [131,273], and with a slight optimization, the Au or Ag @ silicon-based substrates can be a good alternative to conventional substrates, while Ag@Al-based substrates already show comparable to gold and silver detection parameters.

## 9. Conclusions

In this review, we presented and discussed both analytical and clinical SERS applications of less conventional substrates and compared them with staple metal substrates such as silver and gold. As in the last 40 years since the discovery of the SERS phenomena, the need for more cost-effective, scalable, and robust substrates for it increased exponentially with the number of its applications.

Silver and gold remain the predominant/conventional substrate materials since the discovery of the SERS phenomena more than 40 years ago. However, the need for more cost-effective, scalable, and robust substrates for SERS is on the rise as the number of SERS applications continuously increases. This paper not only has presented and discussed analytical and clinical applications of less conventional SERS substrates but also has compared them with similar SERS applications on both silver and gold. According to a literature search, the most prominent alternatives for gold or silver substrates are platinum, palladium, copper, silicon, and aluminum-based substrates. To survey as full substrate landscape (substratescape) as possible we searched for even uncommon substrates such as alloy nanocomposites or HfO_2_-modified nanorods. However, the applicability of the latter substrates is so far much too narrow to compete with gold and silver substrates. Palladium and platinum show sometimes satisfactory performance as SERS substrates usually in combination with other plasmonic metals, but still, they demonstrate on average two orders of magnitude lower SERS enhancement than silver and gold. Moreover, Pt and Pd are more expensive and less commercially available than Au and Ag and this fact drives motivation for their practical SERS applications close to zero.

We observed and described SERS applications of copper-based substrates, which indicated some promising results in combination with gold and silver. However, the problems of surface oxidation and stability make it less attractive as an alternative to conventional substrates. The last two possible alternatives, silicon, and aluminum demonstrated good SERS results in a variety of applications, and further improved their performance in combination with noble metals.

To make an objective comparison between these potential substrates and conventional substrates, we tabulated and calculated averages not only for analytical SERS figures of merits (FOM) (EF, LOD) in the detection of Raman probe R6G, TNT, adenine, etc. but also FOMs for clinical applications of SERS, such as clinical accuracy, sensitivity, and specificity. The results of the comparison showed that silicon and aluminum substrates combined with noble metals, such as gold or silver produce similar LODs and EFs as conventional gold and silver substrates, with an average difference within one order of magnitude at most. However, the silicon itself is almost unusable for SERS applications with its weak plasmonic effects. When we compare performance in the clinical applications, we see the same or even better performance for combined substrates, which use Si and Al surface in combination with gold or silver nanoparticles vs. the results obtained with SERS on pure gold or silver-based substrates. For instance, AgNPs on aluminum substrates showed the best numbers for sensitivity, specificity, and accuracy (93%/100%/96%). In addition, the silicon-based sensors with other metals show 100% accuracy in several cases, which gives promise for its clinical applications. Overall, silicon and aluminum-based substrates, particularly when those are combined with gold or silver nanoparticles show significant promise as an alternative for traditional “gold only” or “silver only” substrates, The advantage of the former (Si or Al-based) substrates include but not limited to: relatively lower cost, better scalability, stability, likely better resistance to contamination with thiols and S-containing compounds; frequently comparable and sometimes superior SERS and clinical assay performance. However, there are still a lot of challenges for SERS on the path to becoming the staple choice for analytical and clinical applications [34].

In the future, the SERS research would try to overcome those challenges. In particular, the SERS research would strive for increase of stability and reproducibility of the SERS substrates, increase in robustness of the signal and improve the affinity of the substrate for the analyte, decrease in cost and time of both substrate preparation and SERS analysis. Those advances would enable the transition of SERS detection from the lab to real life (commercial) sensing applications including applications in clinical analysis.

## Figures and Tables

**Table 3:** The list of SERS studies of different Al-based substrates from 2012 to 2020.

Substrate	Analytes	Analytical Parsameters	Ref
Al NP−film	Adenine, CV (325 nm)	Adenine: EF 3.62 × 10^5^, LOD 10^−6^ M CV: EF 4.1 × 10^5^, LOD 10^−7^	[154]
Al nanocrystals	ssDNA (785 nm)	EF 10^5^–10^6^ LOD 2 × 10^−6^ M	[151]
Al nanodots	NAP, TEPS (532 nm)	EF 7 × 10^4^ EF 1.7 × 10^4^	[153]
AlFON	Adenine (229 nm), (Ru(bpy)_3_)^2+^ MH (355 nm), BPE (405 nm)	EF 10^3^–10^5^	[156]
Al nanovoids	Adenine (244 nm)	EF 5 × 10^3^	[150]
Al nanovoids	Adenine (488 nm, 785 nm)	EF 10^6^	[152]
Al bow-tie nanoantenna	Liquid benzene (258.8 nm)	EF~10^5^	[155]

Abbreviations: NAP—naphthalene, TEPS—triethoxyphenylsilane, CV—crystal violet, R6G –Rhodamine 6G, (Ru(bpy)_3_)^2+^—tris(bipyridine) ruthenium (II), MH—6-mercapto-1-hexanol, BPE—trans-1,2-bis(4-pyridyl)-ethylene, AlFON—aluminum film-over nanosphere.

**Table 4:** List of studies for SERS substrates other than Cu and Al (e.g., Fe_3_O_4_ @Au nanoshells, CoFe_2_O_4_@HNTs/AuNPs, etc.).

Substrate	Analyte	Analytical Parameters	Ref
Fe_3_O_4_ NPs @Au nanoshell	acid orange II, brilliant blue	LOD 6.31 × 10^−7^ M brilliant blue, 2.85 × 10^−3^ M acid orange II RSD 2.49–3.75%, R^2^ = 92–98%	Xie et al., 2019 [182]
CoFe_2_O_4_NPs@HNTs/AuNPs	4,4′-thioaniline, nitrofurantoin	LOD 1.20 × 10^−7^ M 4,4′-thioaniline, 5.88 × 10^−8^ M nitrofurantoin EF 2.7 × 10^7^	Zhang et al., 2020 [183]
Au coated-ZnO NRs	MB	LOD 10^−12^ M	Sinha et al., 2011 [184]
Ag NPs@ZnO	4-ATP	3-fold enhancement compared with hydrophilic Ag@ZnO RSD 11%	Xu et al., 2011 [252]
Ag NPs@MIL-101(Cr) film	4-ATP, nitrofurantoin (NFT)	LOD: 10^−11^ M 4-ATP, 10^−7^ M NFT RSD 5%	Shao et al., 2021 [185]
Au semishells on TiO_2_ spheres	Rhodamine 6G (R6G), brilliant cresyl blue (BCB)	LOD: BCB 10^−7^ M, R6G 10^−6^ M EF 1.4 × 10^5^ RSD 12%	Li et al., 2021 [186]
nanoPAA-ZnCl_2_- AuLs	ctDNA, Surfactant CTAB, SDS, composites ctDNA−CTAB, DNA−CTAB−SDS	LOD: 10^−9^ M EF 9.18 × 10^7^	Hao et al., 2020 [187]
ZnS capped CdSe Ag-QDs	Adenine, guanine, cytosine, thymine, xanthine, and hypoxanthine	LOD: 2 × 10^−6^ M RSD 3.0–6.3% R^2^ = 0.991–0.999	Carrillo-Carrion et al., 2011 [188]
Ag NRs@HfO_2_ shell	CV, MB;2-NAT and 2MPy	LOD 1.25 × 10^−7^ M EF 6.1 × 10^7^ RSD 5.14%	Ma et al., 2016 [189]
ZrO2@Ag@SiO_2_ NPs	4-ATP, R6G	LOD: 10^−9^ M 4-ATP, 10^−8^ M R6G RSD 17.4%	Shi et al., 2020 [190]
TiO_2_ NTs@ AgNPs (diameter 10–12 nm)	R6G	LOD 10^−12^ M EF 9.4 × 10^7^	Filippin et al., 2020 [191]

Abbreviations: MG—malachite green, NB—Nile Blue, MB—methylene blue, 4-ATP—4-aminothiophenol, R6G—Rhodamine 6, APTES—(3-Aminopropyl)triethoxysilane, HNTs—halloysite nanotubes, QD—quantum dots.

**Table 5:** Summary of performance of different SERS substrates for the detection of R6G.

Substrate	Average EF		Average LOD (M)		References
	Arithmetic	Geometric	Arithmetic	Geometric	
Si	$1.9\times 10^{12}$ $\left(33;\;6\times 10^{13}\right)$	$1.7\times 10^{7}$ $\left(33;\;6\times 10^{13}\right)$	$3.7\times 10^{-6}$ $\left(10^{-16};10^{-4}\right)$	$3.8\times 10^{-11}$ $\left(10^{-16};10^{-4}\right)$	[55,66,68,71,75,77,78,82,83,84,85,86,87,88,92,93,100,102,103,105,106,117,119,120,121,124,128,129,230,265]
Pt/Pd	$2.8\times 10^{7}$ $\left(33;\;2.6\times 10^{8}\right)$	$1.8\times 10^{5}$ $\left(33;2.6\times 10^{8}\right)$	$3.4\times 10^{-6}$ $\left(10^{-11};2\times 10^{-5}\right)$	$1.1\times 10^{-9}$ $\left(10^{-11};2\times 10^{-5}\right)$	[55,100,136,137,138,143,147]
Au/Ag	$7.4\times 10^{8}$ $\left(9\times 10^{5};\;6\times 10^{9}\right)$	$5.5\times 10^{7}$ $\left(9\times 10^{5};\;6\times 10^{9}\right)$	$1.3\times 10^{-8}$ $\left(10^{-16};10^{-7}\right)$	$6.1\times 10^{-12}$ $\left(10^{-16};10^{-7}\right)$	[157,158,159,160,161,162,163,164,165,196,197,199,200,258,259,260,261,262,263,264]

Note: The enhancement factors (EF) and limits of detection (LOD) of the substrate group is presented in the following way: average (min; max).

## Data Availability

Not applicable.

## Supplementary Materials

The following supporting information can be downloaded at: https://www.mdpi.com/article/10.3390/bios12110967/s1, Table S1: Multi Elemental Silicon-based SERS substrates (NP: Nanoparticles). Table S2: SERS substrates using pure Si with/or SiO₂ as a substrate. Table S3: Platinum and/or Palladium based SERS substrates. Table S4: Performance of Gold and Silver as SERS substrates. Table S5: The average SERS performance of Si, Pt, Pd based substrates and pure Ag, Au substrates in detection of R6G (analyte). Table S6: The Clinical Performance of Gold and Silver SERS substrates. Table S7: Multi Elemental Aluminum-based SERS substrates. Abbreviations: AAO—anodic aluminium oxide. Table S8: Multi Elemental Copper-based SERS substrates. Abbreviations: CV- crystal violet, R6G—rhodamine 6G, MB—methylene blue. Table S9: The list of SERS studies of different Al-based substrates from 2012 to 2020. Abbreviations: NAP—naphthalene, TEPS—triethoxyphenylsilane, CV—crystal violet, R6G- Rhodamine 6G, (Ru(bpy)_3_)^2+^—tris(bipyridine)ruthenium(II), MH—6-mercapto-1-hexanol, BPE -trans-1,2- bis(4-pyridyl)-ethylene, AlFON—aluminum film-over nanosphere. Table S10: The clinical performance of the SERS studies with different substrates.
