# Supplementary material for: Trends in Application of SERS Substrates beyond Ag and Au, and Their Role in Bioanalysis
Source: Biosensors (Basel). 2022 Nov 3;12(11):967. doi: 10.3390/bios12110967 (PMC9688019; doi:10.3390/bios12110967)
Supplement: Supplementary file 1 [file biosensors-12-00967-s001.zip › biosensors-1962824-supplementary.pdf]

*Supplementary Materials*

# Trends in Application of SERS Substrates beyond Ag and Au, and Their Role in Bioanalysis

**Alisher Sultangaziyev, Aisha Ilyas, Aigerim Dyussupova and Rostislav Bukasov ***

Department of Chemistry, School of Sciences and Humanities (SSH) Nazarbayev University, Kabanbay Batyr av. 53, Nur-Sultan, Kazakhstan
* Correspondence: rostislav.bukasov@nu.edu.kz

Remark

Our classification of substrates into conventional and non-conventional is based on

1) Historic perspective of SERS development since initially SERS was discovered on silver rough film and most of the first, including the most cited, applications of SERS in biodetection were conducted on silver or gold substrates/nanoparticles (glucose and anthrax biomarker by Van Duyne's group, PSA by Porter's group, and many others)

2) We also did a Scopus search for " SERS" and "silver" or "gold" or "silicon" or "copper" or "aluminum" or "platinum" or "palladium" For instance Scopus shows 11,406 document results TITLE-ABS-KEY ( sers AND silver) The numbers of documents found for each of those substrate are 11406 for Ag, 9974 for Au, 2256 for Si , 1066 for Cu , 811 for Al, 474 for Pt, 205 for Pd. Therefore, so called "conventional "Au and Ag substrates are on average about 11 times more common in SERS research literature than average "non-conventional" SERS substrate. Since there is a factor x4.5 difference between search results for Au and for Si, there is hardly much doubt where to draw a border between conventional and non-conventional substates. Even gold substrates alone are likely to be about twice as common in title, abstract and keywords of SERS related publications as all other 5 above mentioned " non-conventional substrates " Therefore term "conventional" ( synonym of the most common or the most frequently used ) for silver and gold substrate materials is well justified.

**Table S1.** Multi Elemental Silicon-based SERS substrates (NP: Nanoparticles).

| Substrate | Analyte | Enhancement Factor | LOD (mol/L) | Details (Laser, power; acquisition time, RSD and etc) | Ref |
|---|---|---|---|---|---|
| AuNP@Si nanowire paper | R6G | 1.00E+05 | 1.00E-08 | 633 nm; RSD <0.15. Detection limit for a pesticide: 2.5*10^-7M; acq t: 10s | [1] |
| AuNP@ Si | Phenylalanine | 2.60E+04 | | 785 nm; Laser power:4 mW; acq t:10 s | [2] |
| AuNP@ porous Si | p-MBA | 3.70E+08 | | 785 nm; RSD 6.7%; acq t: 4s; Laser power 5mW; Au layer is 30nm; with the incr. in the thickness of the Au layer intensity signal decr. | [3] |
| AgNP@Si | p-MBA | 1.00E+08 | | 785 nm; power: 5mW; acq t: 10s Ag layer thickness: 100nm; RSD 7%; | [4] |
| $Al_2O_3$@AgNP@Si nanocones | R6G | 1.00E+09 | 1.00E-12 | 532 nm; Power: 0.05mW Al2O3 thickness 2nm; good stability after 5 months. | [5] |
| AgNP@Si wafer | Pb2+ | 2.00E+06 | 9.90E-11 | 633 nm; Power: 0.2mW; acq t: 1s, LOD for Hg2+: 8.4*10^-10 | [6] |
| AgNP@Si nanowires | R6G | 1.00E+07 | 1.00E-09 | 785 nm | [7] |
| AgNP@meso porous Si | R6G | 1.00E+10 | 1.00E-12 | 514.5nm; acq t: 10s; detection range: (10^-8M; 10^-12M) | [8] |
| AgNP@meso porous Si | Cy5 | 1.00E+07 | 5.00E-09 | 514.5nm; acq t: 10s | [9] |
| AuNP@porousSi | Penicillin G | 5.00E+07 | 1.00E-09 | 532 nm; Power: 10mW; acq t: 3s RSD 4.5%; | [10] |
| Graphene@AgNPs@Si (Aptosensor) | ATP | 8.30E+06 | 1.00E-12 | 633 nm; Power: 20mW; acq t: 1s; substrate size: 140nm; RSD: 10.5% | [11] |
| Si nanowires@GrapheneNP | R6G | 1.00E+07 | | 785 nm; Si is d=50nm cylinder; Au d=60nm sphere on the top of cylinder | [12] |
| CuNP@Si wafer | R6G | 2.29E+07 | 1.00E-09 | 633 nm; acq t: 1s; RSD <20%; CuNP d=20nm | [13] |
| AgNP@Si nanowires | R6G | 6.00E+05 | 1.00E-11 | 633 nm; power: t 1 mW ; acq t: 10s; SiNW thickness is 20 um; AgNP diameter is 40-120nm | [14] |
| AgNP@porous Si photonic crystals | R6G | 1.20E+06 | | 633 nm; power 1mW; acq t 3s; RSD 8%; 3.58 times better EF than just porous Si w/o photonic crystals. | [15] |

| | | | | | |
|---|---|---|---|---|---|
| | Picric Acid | | 1.00E-08 | 532 nm | [15] |
| AgNP@Si nanowires | R6G | 4.12E+09 | 5.00E-09 | 532 nm; power: 0.05 mW; acq t: 10s; | [16] |
| AgNP@meso porous Si | R6G; Cy5 | 1.00E+08 | 1.00E-11 | 514.5 nm; acq t: 10s; | [17] |
| Ag@porous Si wafers | R6G | 2.00E+08 | 1.00E-10 | 514.5 nm; | [18] |
| Ag@mesopor ous Si | Cy3 | 5.30E+12 | 1.00E-14 | 785 nm; acq t: 10s; size of AgNP 5-100nm; | [19] |
| AgNP@porou s Si | NAGase | 1.06E+05 | 1.97E-06 | 785 nm; SERS recovery in comparison to FL technique is 85–98% | [20] |
| PdNP@PSi | imidacloprid (insecticide) | 1.20E+05 | 1.00E-09 | 785 nm; acq t: 20s; substrate stable for at least two weeks; | [21] |
| AgNP@PyrSi | R6G | 1.20E+09 | 1.00E-10 | 532 nm; acq t: 10s; laser spot: 2um; | [22] |
| Au@porousSi | R6G | 1.00E+06 | 1.00E-09 | 785 nm; acq: 10s | [23] |
| AuNP@Si nanowire arrays | RhB | 1.00E+09 | 1.00E-11 | 514 nm; power: 0.5mW; acq t: 10s | [24] |
| AgNP@Si nanowires | R6G | 2.30E+08 | 1.00E-14 | 633 nm; 8mW; acq t: 50s. | [25] |
| SiNP@AgNP @SiC (sandpaper) (Immunoassay ) | PSA (prostate specific antigen) | 3.14E+05 | 5.60E-17 | 785 nm; Si NP size: 50 to 200 nm; LOD: tumor markers PSA: 1.79 fg/ml; AFP:0.46 fg mL−1 ; CA19-9 1.3 × 10−3 U mL−1 | [26] |
| Fe3O4@AgN P@Si nanopillars | malachite green | 1.00E+05 | 1.00E-08 | 532 nm; acq t: 10s; thermoelectrical cooling CCD | [27] |
| AuNP(core)@ $SiO_2$(shell) | Pyridine | 1.00E+06 | | 632.8 nm; power: 1mW; Au core: 55 nm; silica shell: 23 nm; two dimers distance is 2nm | [28] |
| PtNP(core)@S $iO_2$(shell) | Pyridine | 1.00E+05 | | 632.8 nm | [28] |
| PdNP@porous Si | R6G | 100 | | 488 nm; power: 5 mW; acq t: 30s; Pd size 40-86nm; | [29] |
| PtNP@porous Si | R6G | 33 | | 488 nm; power: 5 mW; acq t: 30s; Pt size 56-68 nm; | [29] |
| PtNA@Si wafers | R6G | 5.00E+04 | | 514.5 nm; Power: 0.17 mW, acq t: 30 s; 632.8nm laser has half of 514.5nm's EF | [30] |
| PdNP@porous Si | TNT | 2.54E+06 | 1.00E-07 | 514 nm; power:10 mW, acq t:60 s min gap size (4nm) at etching | [31] |

| | | | | | |
|---|---|---|---|---|---|
| | | | | time of 6min; by weight: Si60%;Pd37% | |
| Ag&Pd alloy NP@Gradient Porosity Si | E.Coli | 2.30E+05 | 1 Cfu/ml | 785 nm; acq t: 10s; best immersion time: 180s; Ag & Pd NP 10nm | [32] |
| PdNP@ pyramidical Si | R6G | 1.91E+06 | 1.00E-11 | 632.8 nm; power: 0.1mW; acq t: 10 s; Pd thick in 60min is 300nm | [33] |
| AuNP@FePt @$SiO_2$ | Adenine | 2.00E+07 | | 633 nm; overall substrate: 30nm | [34] |
| AuNP@Si nanorods | R6G | 3.30E+07 | 1.00E-10 | 633 nm; power: 1.3 mW; acq t:5 s; RSD 3.9−7.2%; SiNR length: 1.3 µm, diameter of 200 nm | [35] |
| Au$SiO_2$ nanorods @ silica gel | R6G | 3.60E+09 | 1.00E-16 | 532 nm; power: 1 mW; acq t: 10 s; Silica gel : 1-2nm, AuSiO2 (70 nm core– 102 nm shell); LOD of CEA antigen 0.86 fg/ml | [36] |
| Au(core)@$SiO_2$(shell) | glucose | 1.20E+08 | 1.00E-12 | 532 nm; power: 20mW; acq t: 10s; AuNP 36nm; silica layer 1-2nm best EF than thicker silica. | [37] |
| AgNP@Si wafer | R6G | 2.90E+07 | | 514 nm; acq t: 1s; RSD <15%; AgNP size is 40nm; | [38] |
| AgNC@SiO2 | R6G | 1.26E+06 | | 532 nm; power: 0.15mW; acq t: 1s; Ag NCs (~50 nm); SiO2 layer is 1.4nm | [39] |
| AuNP@stellate mesoporous SiO2 (Immunoassay) | Ferritin antigen | 1.68E+06 | 6.67E-17 | 785 nm; power: 14mW; acq t: 1s; uNPs (d=10nm) covering SMSiO2 (d=100nm); RSD 22.94%; LOD of ferritin 3.16*10^-14 g/ml | [40] |
| glu@3-MPBA@AuNanoFlower@Si wafer (biosensor) | miRNA 122 | | 7.75E-18 | 785 nm; size of AuNF@Si is 100nm; linear range 10aM-100pM; RSD 0.59% for 6 different samples | [41] |
| $SiO_2$ (core)@AuNPs (shell) Immunoassay | E. coli O157:H7 | | 100 cells/ml | 785 nm; power: 10 mW, acq t: 10 s; substrate is 180nm; assay time 15min; SERS based LFIA strip | [42] |
| AuNP@SiNP | RhB | 1.00E+06 | 1.00E-10 | 785 nm; power: 2mW; acq t: 20s; Au film (10nm) on self assembled SiNP (120nm) | [43] |
| AuNP@P$SiO_2$ | R6G | 6.50E+07 | | 633 nm; power: 1mW; 350nm pores; thickness 60nm; RSD 12%; | [44] |

| | | | | | |
|---|---|---|---|---|---|
| $SiO_2$ NP@AuNP | R6G | | | 633 nm; power: 1mW; adding SiO2 increases EF by 3 times. | [45] |
| $SiO_2$ NP@AgNP | R6G | | | 514nm; power: 1mW; with SiO2 3 times better EF than bare AgNP | [46] |
| AgNP@Au(core)@$SiO_2$(shell) | RdB | 1.03E+05 | 1.00E-11 | 785 nm; acq t: 10s; core diameter of 70 nm and a 5 nm silica shell. | [47] |
| Ag@$SiO_2$ Nanocubes | Melamine for LOD R6G for EF | 1.93E+07 | 4.76E-07 | 632.8 nm; acq t: 30s;SiO2 shell is 2.56nm; AgNP has d=56nm;RSD 10.3%; LOD 0.06mg/L melamine | [48] |
| Ag@SiNWs | 4-ABT | 1.10E+06 | | 632.8 nm; Power: 4.4mW; acq t: 1s; Ag-coated wire d=150nm; Si d=110nm; RSD 14% | [49] |
| AgNano assemblies@Si | R6G | 6.00E+13 | 1.00E-16 | 532 nm; power: 0.6mW; acq t: 0.1s; RSD: 7.3% | [50] |
| Graphene@AgNP@Si fiber | BPA (bisphenol A) | 6.00E+06 | 4.38E-09 | 633 nm; acq t: 4s; AgNP size 60nm; LOD 10^-6 ppm; for 60 sample study recoveries of BPA from 89% to 116% | [51] |
| Graphene@Si nanowires | R6G | 1.00E+05 | 1.00E-07 | 532 nm; power: 1mW; acq t: 10s; NW length 2.56 um; RSD 2.6% | [52] |
| AgNP@mesoporous Si | R6G | | 1.00E-09 | 514.5nm; power: 5mW; acq t: 10s; mesoporous Si 100 times better SERS signal than crystalline Si. | [53] |
| AgNP@Si nanowires | R6G | 3.00E+08 | 1.00E-08 | 514 nm; power: 0.5mW; SiNWs size 1.7um; AgNP d=5-8 nm | [54] |
| AgNP@Si | BPE ( bipyridyl ethylene) | 1.00E+15 | 1.00E-10 | 632.8nm; power: 5 mW; acq t: 5 s; AgNP d=12-22nm; | [55] |
| AgNS@$Cu_2O$@Si nanocones | 4-ATP | 1.00E+07 | 1.00E-14 | 785nm; power: 2mW; acq t: 1s; nanocones: 600-700nm | [56] |
| Au film@PSi | R6G | 1.00E+12 | 1.00E-15 | 785nm; RSD: 6.2%; d=160nm | [57] |
| Au&Ag alloy NP@Si | MB (molecular beacon) | 1.00E+10 | 1.00E-11 | 785nm; 30mW; acq t:5s; RSD <15% | [58] |
| Ag film@Laser Textured SiNP | 4-methylbenzene thiol | 5.50E+06 | | 532nm; Ag optimum thickness 20nm | [59] |
| AgNP@Si Nanopillars | R6G for LOD; PhSH (thiophenol) for EF | 2.40E+08 | 1.00E-13 | 785nm; Si nanopillars of size 80 nm, height 1 um, periodicity 100 nm | [60] |

| | | | | | |
|---|---|---|---|---|---|
| Graphene@Ag film@Laser Textured SiNP | R6G | 2.60E+07 | 1.00E-10 | 532nm; power: 10mW; acq t: 2s; Ag 30nm; stable for more than 50 days | [61] |
| AgNP@ZnO nanowires @Si nanorods | R6G | 1.40E+07 | 1.00E-12 | 532nm; power: 0.37mW; acq t: 5s; RSD 6%; SiNR d=250nm; ZnO NW d=80nm | [62] |
| SiC@Ag(film) @AgNPs (Immunoassay) | PSA, PSMA, and hK2 | 1.14E+07 | 1.50E-08 | 785nm; LOD 0.46 fg mL−1 (PSA);1.05 fg mL−1(PSMA) and 0.67 fg mL−1 (hK2) | [63] |
| AgNP@Si | N. gonorrhoeae | 1.00E+08 | LOD 100 cfu/ml | 785nm; acq t: 6s; Si wafers (3mmx3mm used) & AgNP layer 100nm | [64] |
| Au@ZnO@Si | Neoprotein | | 1.60E-09 | 785nm; power: 5mW: acq t: 10s; Au layer 90nm; ZnO layer; 1.4um | [65] |
| AgNP@Si wafer | miRNAs | 2.00E+06 | 1.00E-18 | 633nm; power: 0.2mW; acq t: 1s | [66] |
| AgNP@ Si wafer | TNT | 6.40E+06 | 1.00E-12 | 514 nm, acq t: 1s | [67] |
| AuNP@Si Immunoassay sandwich | hIgG | | 3.00E-12 | 633 nm and 785nm | [68] |
| AuNanothorn @Si wafer | R6G | 1.9E+07 | 4.50E-13 | | [69] |
| AuNP@Si | R6G | 2.5E+08 | 1.00E-11 | 633 nm, power: 0.94mW | [70] |
| AgNC@SiO2 @PMHS paper | adenine | 6.55E+06 | 8.90E-10 | 633 nm, power: 0.3 mW, acq t: 2 s, stability up to 90 days | [71] |

**Table S2.** SERS substrates using pure Si with/or $SiO_2$ as a substrate.

| Substrate | Analyte | Enhanceme nt Factor | LOD | Details (Laser, power; acquisition time, RSD and etc) | Ref |
|---|---|---|---|---|---|
| black Si | PATP-to-DMAB | 1.00E+03 | 1.00E-06 | 532nm; para-aminothiophenol (PATP)-to-4,4’-dimercaptoazobenzene (DMAB) reaction was observed. | [72] |
| Si Nano Wires | R6G | | 1.00E-04 | 633nm; power: 8mW; acq t: 50s; | [25] |
| $SiO_2$ Nano Wires | Interleukin 10 | 4.00E+07 | 5.55E-12 | 415.5nm; power: 24.6 mW; acq t: 10 s; LOD 0.1ng/ml ; EF of Au@SiO2 substrate is 2.7*10^3 | [73] |
| Laser irradiated Si structure | Malachite green | 500 | | 533 nm; acq t: 1s; spongiform Si structures of average dimension of 300 nm | [74] |
| Si Nano Spheroids | R6G | 5.38E+06 | | 785 nm; acq t: 3s; substrate is composed of both crystalline and amorphous Si and amorphous SiO2. | [75] |
| Si@$SiO_2$ | HeLa cancer | 1.00E+07 | 1.00E-12 | 785 nm; acq t: 180s/line; in situ live bioimagining | [76] |

**Table S3.** Platinum and/or Palladium based SERS substrates.

| Substrate | Analyte | Enhancement Factor | LOD | Details (Laser, power; acquisition time, RSD and etc) | Ref |
|---|---|---|---|---|---|
| Anodic Al oxide@ Pt Metal Film | Methylene blue | 1.88E+05 | 1.00E-09 | 532nm; power: 0.01mW; acq t: 1s; RSD 7.14% for 5 signals | [77] |
| PtNP@AgNP@ Ge wafers | R6G | 9.10E+06 | | 633 nm; acq t: 1s; RSD <10% for 100 spots; Pt w% is 8.57% | [78] |
| PtNP | pyridine | 2000 | | 632.8 nm; size of PtNP 10-20nm | [79] |
| PtNP (core @ SiO2 (shell) | Pyridine | 1.00E+05 | | 632.8 nm; EF obtained for electrochemically roughened platinum electrodes is about 100. | [28] |
| AgNP@4-ABT@Pt | 4-ABT | 790 | | 514.5nm; acq t: 30s; Pt surface 400 nm ×400 nm ×150 nm. | [80] |
| Pt@TiO2 Nanotube Arrays | R6G | 4.30E+04 | 2.00E-05 | 532 nm; power: 0.5mW; acq t: 5s; polydopamine layer is on vertically aligned TiO2 nanotube. | [81] |
| AgNanoCubes @ pATP@Pt (sandwich) | p-ATP | 4.10E+06 | | 532 nm; no signal w/o AgNC; signal increases as the thickness of Pt film from 42 to 90nm | [82] |
| PdNP @ AuSSV (sphere segment void) | pyridine | 7.80E+03 | | 632.8nm; power: 3mW; EF decreases with the thickness of Pd layer | [83] |
| PtNP @ AuSSV (sphere segment void) | pyridine | 4.90E+03 | | 632.8nm; power: 3mW; EF decreases with the thickness of Pt layer | [83] |
| PdNP @ porous Si | R6G | 100 | | 488 nm; power: 5mW; acq t; 30s; size of PdNP 40-86 nm | [29] |
| PtNP @ porous Si | R6G | 33 | | 488 nm; power: 5mW; acq t; 30s; size of PtNP 56-68 nm | [29] |
| PtNA@Si wafers | R6G | 5.00E+04 | | 515nm;power:0.17 mW;acq t:30 s | [30] |
| AuNP shell @ PdNP core | p-ATP | 5.00E+04 | | 632.8nm; power: 1mW; 135 nm Au covering 0.7 nm Pd (about two atomic layer of Pd) | [84] |
| PdNP@porous Si | TNT | 2.54E+06 | 1.00E-07 | 514 nm; power: 10mW; acq t: 60s; w%: Si is 60%; Pd is 37% | [31] |

| | | | | | |
|---|---|---|---|---|---|
| Ag&PdNP@Gradient porosity Si | E.Coli | 2.30E+05 | 1 Cfu/ml | 785 nm; acq t: 10s; NP is 10nm; optimum 180s immersion time | [32] |
| Au&Pd alloy@$Cu_2O$/CuO | CV | 5.00E+05 | 1.00E-06 | 488 nm; acq t: 10s; size:160nm; EF increases with the thickness of alloy; 100% self-recovery in 1 min | [85] |
| PdNP@Si | R6G | 8.54E+05 | 1.00E-10 | 682.8nm; power: 0.1mW; acq t: 10s; Pd thickness is 300nm | [33] |
| PdNP@ pyramidical Si | R6G | 1.91E+06 | 1.00E-11 | 682.8nm; power: 0.1mW; acq t: 10s; Pd thickness is 300nm | [33] |
| PdNP@ Ge | R6G | 3.48E+05 | 1.00E-09 | 682.8nm; power: 0.1mW; acq t: 10s; Pd thickness is 64.4 nm | [33] |
| Polyvinyl alcohol scaffold@PdNW | MB (methylene blue) | 1.29E+02 | 1.00E-05 | 532 nm; Diameter: 55–70 nm, length; 3–4 µm | [86] |
| CTAB@Pd nanocubes | MB (methylene blue) | 1.93E+03 | 1.00E-06 | 532nm; side length: 37 ± 5 nm | [86] |
| DNA@PdNP | MB (methylene blue) | 1.20E+05 | 1.00E-08 | 532 nm; size of the PdNPs is 3.5- 5 nm. | [86] |
| AuPdNanoBrambles | Crystal Violet | 5.37E+06 | 8.00E-08 | 633 nm; power: 0.5mW: acq t: 10s; w% of Au is 46%, Pd is 54% | [87] |
| PdAu Hollow NanoChains | R6G | 3.50E+05 | 1.00E-08 | 633nm; power: 1.7mW; acq t: 10s; w% of Pd is 4 times more Au | [88] |
| PdNP@Au | 4-ABT | 4.80E+03 | | 632.8nm; power: 10mW; acq t: 10s; triangular 100nm Pd with height of 300-400nm | [89] |
| PdNP | 4-ABT | 1.90E+09 | 1.00E-10 | 633 nm; power: 10mW; acq t: 5s; | [90] |
| Nanocrystalline Pd films@Si | thiophenol | 1.20E+05 | | 632.8 nm; power: 5mW; acq t: 10s; NP size 50-60nm | [91] |
| Ag/Pd alloy NP | R6G | 2.62E+08 | 1.00E-09 | 532 nm; power: 30mW; w% of Pd is 18%; SERS activity dec with inc in w% of Pd; stable for 20 days. | [92] |

| | | | | | |
|---|---|---|---|---|---|
| PdNP | 4-mercaptopyridine; | 1.70E+05 | | 514nm; power: <0.5mW; acq t: 10s; | [93] |
| Flake PdNP@Au surface | 4-ABT | 1.50E+03 | | 632.8nm; power: 8.5mW; | [94] |
| PdNP | pyridine | 1.80E+03 | | 632.8nm; power: 7mW;size 50nm | [95] |
| AuNP@FePt@ $SiO_2$ | adenine | 2.00E+07 | | 633 nm; substrate diameter 30nm | [34] |
| Ag/Au/Pt Nanocages | Rhodamine 3B | 4.70E+09 | 1.00E-15 | 532 nm; acq t: 10s; w% Ag: 63%, Au: 16%; Pt 21%, | [96] |

**Table S4**. Performance of Gold and Silver as SERS substrates.

| Substrate | Analyte | Enhancement Factor | LOD | Details (Laser; power; acquisition time; RSD and etc) | Ref |
|---|---|---|---|---|---|
| Ag | 4-Mercaptobenzoic acid | 7.33E+07 | 1.00E-10 | 785 nm, | [97] |
| Ag Nano Sheets@opal | R6G | 6.00E+07 | 1.00E-14 | 514 nm, power: 0.17mW; Ag nanosheets are 370nm | [98] |
| Ag Nano Cubes | R6G | 2.00E+08 | 1 molecule detection | 532 nm, power: 60mW; acq t: 5s | [99] |
| AuNP | 4-Mercaptobenzoic acid | 1.10E+09 | 1-2 molecule detection | 785 nm; AuNP with hollows 20 to 45 nm, | [100] |
| AuAg allow nanoporous film | R6G | 3.00E+08 | 1.00E-12 | 785 nm; power: 0.1mW; EF of hotspots: 10E+10 to 10E+11, w% of allow Au 79%, Ag 21% | [101] |
| Au Nano Flowers | MUC 4 (pancreatic cancer marker) | 7.30E+06 | 1.00E-13 | 785 nm; LOD was 0.1ng/ml; MUC 4 MW was taken to be 1000kDA | [102] |
| Ag/Au Nano Cubes | R6G | 6.10E+09 | 1.00E-16 | 532 nm; acq t: 10s; | [103] |
| AuNP | hIgG | | 2.80E-11 | 633 nm; power: 5mW; acq t: 5s; Immunoassay sandwich; antigen used; AuNP 60nm; were modified with Raman reporters (4-NBT) | [68] |
| AuNP@cysteine | TNT | 1.0E+09 | 2.00E-12 | 670nm, power: 2mW | [104] |
| Ag nano ribbons | TNT | 1.0E+07 | 2.50E-08 | 532 nm, 1ps laser and input energy of 1200mJ was used to synthesize Ag nano ribbons. | [105] |

| | | | | | |
|---|---|---|---|---|---|
| Au nanofilms/cicada wing | R6G | 2.2E+06 | 1.00E-08 | | [106] |
| Ag pyramids | R6G | 9.1E+05 | 1.00E-10 | 532 nm | [107] |
| Ag&AuNP | R6G | 5.0E+08 | 2.00E-14 | 785 nm, power: 1mW | [108] |
| AuNP | R6G | 5.8E+07 | 2.00E-12 | | [109] |
| AuNP | R6G | | 3.00E-09 | 638 nm, acq r: 1s | [110] |
| AuNP@polysterene | R6G | 1.8E+08 | | 473 nm, size of Au 5nM | [111] |
| AgNP@cicada wings | R6G | 1.0E+06 | 1.00E-07 | 785 nm, power: 0.325mW, acq t: 1s | [112] |
| AgNP@filter paper | adenine | 1.00E+07 | 1.60E-07 | 785 nm | [113] |
| Ag@chitosan flakes | adenine | | 1.20E-11 | 633 nm | [114] |

**Table S5.** The average SERS performance of Si, Pt, Pd based substrates and pure Ag, Au substrates in detection of R6G ( analyte).

| Substrate | Average EF | | Average LOD (M) | | References |
|---|---|---|---|---|---|
| | Arithmetic | Geometric | Arithmetic | Geometric | |
| Pure Si | $5.38 \times 10^6$ (only one EF) | | $10^{-4}$ (only one LOD) | | [25,74] |
| Si with metals | $2.0 \times 10^{12}$ $(33;\ 6 \times 10^{13})$ | $1.8 \times 10^7$ $(33;\ 6 \times 10^{13})$ | $5.0 \times 10^{-9}$ $(10^{-16}; 10^{-7})$ | $2.2 \times 10^{-11}$ $(10^{-16}; 10^{-7})$ | [1,5,7,8,12-18,22,23,25,29,33,35,36,38,39,50,52-54,57,61,62,69,70] |
| | | | | | |
| Pt | $2.3 \times 10^6$ $(33; 9.1 \times 10^6)$ | $2.8.\times 10^4$ $(33;\ 9.1 \times 10^6)$ | $2.0 \times 10^{-5}$ (Only one LOD available) | | [29,30,78,81] |
| Pd | $4.4 \times 10^7$ $(100; 2.6 \times 10^8)$ | $4.6 \times 10^5$ $(100;\ 2.6 \times 10^8)$ | $2.4 \times 10^{-9}$ $(10^{-11}; 10^{-8})$ | $3.2 \times 10^{-10}$ $(10^{-11}; 10^{-8})$ | [29,33,88,92] |
| | | | | | |
| Al+Au | $8.1 \times 10^6$ | | $7 \times 10^{-10}$ $(10^{-10}; 10^{-9})$ | $3.16 \times 10^{-10}$ $(10^{-10}; 10^{-9})$ | [115], [116], [117], [118] |
| Al+Ag | $5.39 \times 10^7$ $(10^7; 9.77 \times 10^7)$ | $3.13 \times 10^7$ $(10^7; 9.77 \times 10^7)$ | $2.50 \times 10^{-7}$ $(10^{-15}; 10^{-6})$ | $1.78 \times 10^{-11}$ $(10^{-15}; 10^{-6})$ | [119-123] |
| Au | $8.0 \times 10^7$ $(2 \times 10^6, 2 \times 10^8)$ | $2.8 \times 10^7$ $(6 \times 10^7, 2 \times 10^8)$ | $4.3 \times 10^{-9}$ $(2 \times 10^{-12}; 10^{-8})$ | $3.9 \times 10^{-10}$ $(2 \times 10^{-12}; 10^{-8})$ | [106,109-111] |
| Ag | $6.6 \times 10^7$ $(9 \times 10^5;\ 2 \times 10^8)$ | $1.0 \times 10^7$ $(9 \times 10^5;\ 2 \times 10^8)$ | $3.34 \times 10^{-8}$ $(10^{-14}; 10^{-7})$ | $4.6 \times 10^{-11}$ $(10^{-14}; 10^{-7})$ | [98,99,107,112] |
| | | | | | |

**Table S6.** The Clinical Performance of Gold and Silver SERS substrates.

| Substrate | Diagnosis of | Analyte, biomarker | Sensitivity | Specificity | Accuracy | N of sample (positie+, negative -) | Ref |
|---|---|---|---|---|---|---|---|
| AuNP | 7 different cancer cells | cells | | | 81.2% | 245 | [124] |
| AuNP | Cervical cancer | Serum | 100% | 100% | 100% | 36 (24+,12-) | [125] |
| AuNP | Oral squamous cell carcinoma | Serum | 80.7% | 84.1% | 82.5% | 280 (135+, 145-) | [126] |
| AuNP | Colorectal cancer | Serum | 97.4% | 100% | 98.8% | 83 (38+,45-) | [127] |
| Au chip | Lung Cancer | Saliva | 100% | 100% | 100% | 127 (61+,66-) | [128] |
| AuNP | Prostate cancer | Urine | 100% | 89% | 95% | 18 (9+,9-) | [129] |
| AuNP | Colorectal Cancer | Serum,carcino embryonic antigen | 90% | 100% | 91.8% | 98 (80+,18-) | [130] |
| AgNP | Colorectal Cancer | Purified serum, Albumin | 100% | 100% | 100% | 206 (103+, 103-) | [131] |
| AgNP | Liver cancer | Serum | 92.3% | 100% | 97.3% | 75 (26+, 49-) | [132] |
| | Nasopharyngeal cancer | Serum | 96.0% | 88.0% | 90.7% | 75 (25+, 50-) | [132] |
| AgNP | Coronary heart disease | Urine | 80.9% | 92.1% | 84.1% | 220 (157+, 63-) | [133] |
| AgNP | Prostate Cancer | Serum, PSA | 95% | 93.8% | 94.2% | 120 (80+, 40-) | [134] |
| AgNP | Breast Cancer | Saliva, sialic acid | 94% | 98% | 96.1% | 206 (100+, 106-) | [135] |
| AgNP (SERS-CRISPR) | SARS-COV-2 | RNA extracts, viral N gene | 87.5% | 100% | 97.3% | 112 (24+, 88-) | [136] |
| AgNP | Prostate Cancer | Serum & PSA | 100% | 87.5% | 94% | 54 (30+,2 4-) | [137] |

Overall performance of the methods shown below are used in the review to compare the efficiency of Au, Ag substrates in clinical diagnosis in comparison to non-conventional SERS susbstrates as Si, Fe, Al and etc.

**Table S7.** Multi Elemental Aluminum-based SERS substrates. Abbreviations: AAO - anodic aluminium oxide.

| | Ref | Author et. al | Year | Analyte | Substrate | Analytical parameters |
|---|---|---|---|---|---|---|
| Al+Ag | [138] | Chang et al. | 2020 | R6G | ZnO: Al/Ag heterostructure | LOD $10^{-10}$ M |
| | [119] | Chang et al. | 2019 | R6G | Al-doped ZnO@SnO2@Ag heteronanowires | LOD $10^{-12}$ M Substrate can maintain similar SERS-enhancing effect even after five cycles. |
| | [120] | Shan et al. | 2014 | R6G | AAO/Al-based Ag nanostructure arrays | EF $9.77\times10^{7}$ |
| | [122] | Das et al. | 2021 | R6G | silver capped aluminium nanorods | LOD $10^{-15}$ M<br>EF $10^{7}$ |
| | [123] | Zhang et al. | 2018 | R6G | AgNPs/$MnO_2$@Al | LOD $10^{-6}$ M |
| | Geometric mean for LOD: $1.78\times10^{-11}$ M<br>Geometric mean for EF: $3.13\times10^{7}$ | | | | | |
| Al+Au | [115] | Hou et al. | 2014 | R6G | Au nanoparticles on hexagonally patterned bowl-shaped-dimples on Al foil | LOD $10^{-9}$ M |
| | [116] | Sui et al. | 2016 | R6G | Au-$CuCl_2$-AAO | LOD $10^{-10}$ M<br>EF $2.3\times10^{7}$ |
| | [117] | Nielsen et al. | 2009 | R6G | Gold Nanostructures with Sub-10 nm Gaps on a Porous Al2O3 Template | LOD $10^{-9}$ M |
| | [118] | Choi et al. | 2010 | R6G | Au-AAO | EF $0.81\times10^{7}$ |
| | Geometric mean for LOD: $3.16\times10^{-10}$ M<br>Geometric mean for EF: $8.10\times10^{6}$ | | | | | |

**Table S8.** Multi Elemental Copper-based SERS substrates. Abbreviations: CV- crystal violet, R6G – rhodamine 6G, MB – methylene blue.

| | Ref | Author et al. | Year | Substrate morphology | Analytes | Analytical parameters |
|---|---|---|---|---|---|---|
| Cu+Ag | [139] | Fodjo et al. | 2012 | Ag/b-AgVO3 nanobelts deposited on copper foil (Cu@Ag/b-AgVO3) | carbaryl carbofuran isoprocarb propoxur (carbamate pesticides) | LOD: 2.5×10-12 M 10×10-12 M 50×10-12 M 75×10-12 M EF $10^6$ RSD 4.3% |
| | [140] | Dai et al. | 2021 | Cu-coated fabric, Cu-Ag-coated fabric | CV | LOD $10^{-8}$ M EF $2\times10^6$ |
| | [141] | Liu et al. | 2020 | Complex of CuO@Ag nanowires on Cu mesh (Cu/CuO@Ag) | R6G | LOD $10^{-15}$ M EF $4.88\times10^{11}$ |
| | [142] | Rao et al. | 2017 | Cu−Ag−PVA(with different Ag contents) | R6G MB | LOD: $3.30\times10^{-8}$ R6G, $1.60\times10^{-8}$ MB EF $10^7$-$10^8$ |
| | [143] | Zhang et al. | 2018 | graphene/bilayer silver/Cu sandwich | CV, R6G | LOD: $10^{-9}$ CV, $10^{-8}$ R6G EF $1.19\times10^5$ RSD <5.9% $R^2$ 0.935 for CV $R^2$ 0.974 for R6G |
| | [144] | Sravani et al. | 2021 | Ag−Cu alloy microflowers | R6G | LOD $10^{-21}$ M EF $10^8$ |
| | [145] | Fu et al. | 2020 | Superhydrophobic nanostructured Cu with Ag NPs | R6G | LOD $10^{-13}$ M EF $1.2\times10^5$ |
| | Geometric LOD: $5.30\times10^{-12}$M Geometric EF: $1.76\times10^7$ | | | | | |
| Cu+Au | [146] | Bankowska et al. | 2016 | Au−Cu layer on photoetched GaN | pyridine, 4-mercaptobenzoic acid | EF $5\times10^5$ |
| | [147] | Sakir et al. | 2020 | Cu NSs from Au nanoparticles (NPs) that were attached to the substrate through a layer of end-grafted | R6G | LOD $10^{-8}$ M EF $2.95\times10^5$ RSD 9.4% $R^2$=0.973 |

| | | | | | | |
|---|---|---|---|---|---|---|
| | | | | poly(ethylene glycol). | | |
| | [148] | Chen et al. | 2017 | Au/Cu hybrid nanostructure arrays | Urea | LOD $10^{-3}$ M |
| | [149] | Manish et al. | 2020 | Au–Cu alloy nanostructures | MB | EF $1.2\times10^{3}$ |
| | [150] | Siva et al. | 2020 | Flower-Shaped Au–Cu Nanostructures | CV | LOD $10^{-10}$ M<br>EF $0.21\times10^{6}$ |
| | Geometric LOD: $1.00\times10^{-7}$ M<br>Geometric EF: $7.81\times10^{4}$ | | | | | |

**Table S9.** The list of SERS studies of different Al-based substrates from 2012 to 2020. Abbreviations: NAP – naphthalene, TEPS – triethoxyphenylsilane, CV – crystal violet, R6G- Rhodamine 6G, $(Ru(bpy)_3)^{2+}$ - tris(bipyridine)ruthenium(II), MH - 6-mercapto-1-hexanol, BPE -trans-1,2- bis(4-pyridyl)-ethylene, AlFON - aluminum film-over nanosphere.

| Substrate | Substrate preparation | Analytes | Analytical parameters | Ref |
|---|---|---|---|---|
| Al NP−film | Thickness of Al nanosheets from which Al NPs were produced – 2 nm. Al nanosheets deposited on Al film at a rate 8 Å /s under $10^{-6}$ Pa pressure | Adenine, CV (325 nm) | For adenine: EF $3.62\times10^5$, LOD $10^{-6}$ M<br>For CV: EF $4.1\times10^5$, LOD $10^{-7}$ | [151] |
| Al nanocrystals | SERS and normal Raman spectra were measured under 0.0038, 0.038, 3.8 mW laser power and 10, 40, 10 s integration time. The SERS spectra on Al substrate were averaged over 50 scans. | ssDNA (785 nm) | EF $10^5$-$10^6$<br>LOD $2\times10^{-6}$ M | [152] |
| Al nanodots | Polymeric nanostructures were fabricated using the Nanoscribe® Photonic Professional (Nanoscribe Inc., Germany). All structures were written on square glass substrates (width = 22 mm and thickness ≈ 0.13 to 0.16 mm). | NAP, TEPS (532 nm) | EF $7\times10^4$<br>EF $1.7\times10^4$ | [153] |
| Aluminum film-over nanosphere (AlFON) | nanosphere sizes: 170 nm silica spheres (Bangs Laboratories, SS02N, washed once and diluted prior to use) and 210 nm carboxylated latex/polystyrene spheres (Life Technologies, C37486, used as received). | Adenine (229 nm),$(Ru(bpy)_3)^{2+}$ MH (355 nm), BPE (405 nm) | EF $10^3$-$10^5$ | [154] |
| Al nanovoids | Large areas of Al nanovoids were fabricated via nanoimprint lithography. A layer of 50 nm Al was evaporated onto the nanovoids via E-beam evaporation (Lesker) to form the film of Al nanovoids. | Adenine (244 nm) | EF $5\times10^3$ | [155] |

| | | | | |
|---|---|---|---|---|
| Al nanovoids | optical power density on the sample was 1500 W/cm2 . The exposure time was 30 s, and 10 acquisitions were performed for each spectrum. | Adenine (488 nm, 785 nm) | EF $10^6$ | [156] |
| Al bow-tie nanoantenna | 50 nm thick aluminum bowtie antenna was formed by two equilateral triangles of 100 nm side length with 20 nm apex to apex gap distance. | Liquid benzene (258.8 nm) | EF ~$10^5$ | [157] |
| Geometric mean for LOD: $5.85\times10^{-7}$ M, geometric mean for EF: $1.27\times10^5$ | | | | |

**Table S10.** The clinical performance of the SERS studies with different substrates.

| Substrate | Diagnosis of | Analyte | Sensitivity | Specificity | Accuracy | N of samples | Ref |
|---|---|---|---|---|---|---|---|
| SiO2(core)@Ag(shell) (immunoassay) | SARS-COV-2 | Serum,IgM/IgG | 100% | 100% | 100% | 68 (19+, 49-) | [158] |
| SiC@Ag(film)@AgNPs (immunoassay) | Prostate cancer | Serum, (biomarkers: PSA, PSMA, hK2) | 70% | - | 70% | 10+ | [63] |
| | benign prostate hyperplasia | Serum, PSA, PSMA, hK2 | 60% | - | 60% | 10+ | [63] |
| | Healthy samples | Serum, PSA, PSMA, hK2 | - | 75% | 75% | 12- | [63] |
| AgNP@Si | N. gonorrhoeae | Urine, N. gonorrhoeae | 100% | 100% | 100% | 120 spectra (60+,60-) | [64] |
| Au@ZnO@Si | Neisseria meningitidis | Cerebrospinal fluid, neoprotein | 95% | 98% | - | N/A | [65] |
| AgNP@Si wafer | Breast cancer | Serum, 9 miRNAs | - | - | 85% | 25, (7+,6+, 5+,4+, 3-) | [66] |
| Si@SiO2 | HeLa cancer | cells | 86% | 94% | - | N/A | [76] |
| AgNP@pyramidical Si | Lung Cancer | serum | 100% | 90% | 95% | 100 (50+, 50-) | [159] |
| AgNP @ porous Si | Breast Cancer | Serum | 93.3% | 96.7% | 95% | 60 (30+,30-) | [160] |
| Si wafers @ Ag @ AuNP | Hepatocellular carcinoma | Serum | 100% | 100% | 100% | 80 (20+, 60-) | [161] |
| SiO2 @ AuNP | Lung Cancer | Serum | - | - | 90.7% | 116 (58+, 58-) | [162] |

| | | | | | | | |
|---|---|---|---|---|---|---|---|
| 2D Au–Si substrate and upper Ag@4-MBA@Au core-shell nanoparticles. | Breast Cancer | miRNA-21 and miRNA-155 in serum | 100% | 100% | 100% | 60 (30+, 30-) | [163] |
| Ag coated Si Nanopillar | Lung Cancer | - | 87% | 83% | 85% | N/A | [164] |
| Au cubes on Si | SARS-CoV-2 virus | Saliva | 95% | 95% | 95.2% | 42 (21+, 21-) | [165] |
| Ag @ Si | Neoplastic Tissue | Salivary gland homogenates | 97% | 89% | 93% | 6 (3+, 3-) 120 spectra | [166] |
| Al foil | Colorectal Cancer | Serum | 83% | 83% | 83.3% | 60 (30+,30-) | [167] |
| AgNP@Al foil | Prostate Cancer | Serum, PSA | 87% | 100% | 94% | 54 (30+,24-) | [137] |
| AgNP@Al plate | Prostate Cancer | Serum, PSA | 84.9% | 100% | 91.3% | 161 (93+,68-) | [168] |
| Ag Colloid@Al foil | bladder cancer, NMIBC, MIBC | Serum | - | - | 93.3% | 149 (27+,32+, 60+,30-) | [169] |
| AgNR wrapped with $Al_2O_3$ | lung adenocarcinoma (LAC) | Serum | 98.1% | 97.6% | - | 190 (108+, 82-) | [170] |
| AgNP@Al plate | bladder cancer | Serum | - | - | 96.4% | 88 (48+,40-) | [171] |
| | differentiating bladder cancer | Serum | - | - | 95.4% | 88 (25+,23+, 40-) | [171] |
| AgNP@Al plate | Bladder Cancer | Serum | 90.9% | 100% | 94.5% | 91 (55+,36-) | [172] |
| Ag colloid@Al foil | Prostate cancer, benign | Plasma, PSA | 95.8% | 100% | 97.9% | 28 (14+,14-) | [173] |

| | | | | | | | |
|---|---|---|---|---|---|---|---|
| | prostatic hyperplasia | | | | | | |

Note: The sensitivity/specificity/accuracy of the overall substrate group is presented in the following way: average (min; max). The number of samples: All samples (positive "+", negative "-') NP – nanoparticles, PSA - Prostate Specific Antigen. Ref 124 is included in the table, but the parameters reported in this paper are not used for the calculation of averages, since the number of patients tested in the study (10 to 12) is too low to consider the reported parameters as conclusive statistical values. Abbreviations: non-muscle-invasive bladder cancer (NMIBC) and muscle-invasive bladder cancer (MIBC).

## References


1. Cui, H.; Li, S.; Deng, S.; Chen, H.; Wang, C. Flexible, Transparent, and Free-Standing Silicon Nanowire SERS Platform for in Situ Food Inspection. *ACS Sens* **2017**, *2*, 386-393, doi:10.1021/acssensors.6b00712.
2. Kaminska, A.; Inya-Agha, O.; Forster, R.J.; Keyes, T.E. Chemically bound gold nanoparticle arrays on silicon: assembly, properties and SERS study of protein interactions. *Phys Chem Chem Phys* **2008**, *10*, 4172-4180, doi:10.1039/b803007c.
3. Kaminska, A.; Szymborski, T.; Jaroch, T.; Zmyslowski, A.; Szterk, A. Gold-capped silicon for ultrasensitive SERS-biosensing: Towards human biofluids analysis. *Mater Sci Eng C Mater Biol Appl* **2018**, *84*, 208-217, doi:10.1016/j.msec.2017.11.029.
4. Szymborski, T.; Stepanenko, Y.; Niciński, K.; Piecyk, P.; Berus, S.M.; Adamczyk-Popławska, M.; Kamińska, A. Ultrasensitive SERS platform made via femtosecond laser micromachining for biomedical applications. *Journal of Materials Research and Technology* **2021**, *12*, 1496-1507, doi:10.1016/j.jmrt.2021.03.083.
5. Wang, Z.; Zheng, C.; Zhang, P.; Huang, Z.; Zhu, C.; Wang, X.; Hu, X.; Yan, J. A split-type structure of Ag nanoparticles and Al2O3@Ag@Si nanocone arrays: an ingenious strategy for SERS-based detection. *Nanoscale* **2020**, *12*, 4359-4365, doi:10.1039/c9nr09238b.
6. Shi, Y.; Chen, N.; Su, Y.; Wang, H.; He, Y. Silicon nanohybrid-based SERS chips armed with an internal standard for broad-range, sensitive and reproducible simultaneous quantification of lead(ii) and mercury(ii) in real systems. *Nanoscale* **2018**, *10*, 4010-4018, doi:10.1039/c7nr07935d.
7. Fang, C.; Agarwal, A.; Widjaja, E.; Garland, M.V.; Wong, S.M.; Linn, L.; Khalid, N.M.; Salim, S.M.; Balasubramanian, N. Metallization of Silicon Nanowires and SERS Response from a Single Metallized Nanowire. *Chemistry of Materials* **2009**, *21*, 3542-3548, doi:10.1021/cm900132j.
8. Virga, A.; Rivolo, P.; Frascella, F.; Angelini, A.; Descrovi, E.; Geobaldo, F.; Giorgis, F. Silver Nanoparticles on Porous Silicon: Approaching Single Molecule Detection in Resonant SERS Regime. *The Journal of Physical Chemistry C* **2013**, *117*, 20139-20145, doi:10.1021/jp405117p.
9. Virga, A.; Rivolo, P.; Descrovi, E.; Chiolerio, A.; Digregorio, G.; Frascella, F.; Soster, M.; Bussolino, F.; Marchiò, S.; Geobaldo, F.; et al. SERS active Ag nanoparticles in mesoporous silicon: detection of organic molecules and peptide-antibody assays. *Journal of Raman Spectroscopy* **2012**, *43*, 730-736, doi:10.1002/jrs.3086.
10. Wali, L.A.; Hasan, K.K.; Alwan, A.M. Rapid and Highly Efficient Detection of Ultra-low Concentration of Penicillin G by Gold Nanoparticles/Porous Silicon SERS Active

Substrate. *Spectrochim Acta A Mol Biomol Spectrosc* **2019**, *206*, 31-36, doi:10.1016/j.saa.2018.07.103.
11. Meng, X.; Wang, H.; Chen, N.; Ding, P.; Shi, H.; Zhai, X.; Su, Y.; He, Y. A Graphene-Silver Nanoparticle-Silicon Sandwich SERS Chip for Quantitative Detection of Molecules and Capture, Discrimination, and Inactivation of Bacteria. *Anal Chem* **2018**, *90*, 5646-5653, doi:10.1021/acs.analchem.7b05139.
12. Li, Y.; Dykes, J.; Gilliam, T.; Chopra, N. A new heterostructured SERS substrate: free-standing silicon nanowires decorated with graphene-encapsulated gold nanoparticles. *Nanoscale* **2017**, *9*, 5263-5272, doi:10.1039/c6nr09896g.
13. Shao, Q.; Que, R.; Shao, M.; Cheng, L.; Lee, S.-T. Copper Nanoparticles Grafted on a Silicon Wafer and Their Excellent Surface-Enhanced Raman Scattering. *Advanced Functional Materials* **2012**, *22*, 2067-2070, doi:10.1002/adfm.201102943.
14. Ouhibi, A.; Saadaoui, M.; Lorrain, N.; Guendouz, M.; Raouafi, N.; Moadhen, A. Application of Doehlert Matrix for an Optimized Preparation of a Surface-Enhanced Raman Spectroscopy (SERS) Substrate Based on Silicon Nanowires for Ultrasensitive Detection of Rhodamine 6G. *Appl Spectrosc* **2020**, *74*, 168-177, doi:10.1177/0003702819881222.
15. Zhong, F.; Wu, Z.; Guo, J.; Jia, D. Porous Silicon Photonic Crystals Coated with Ag Nanoparticles as Efficient Substrates for Detecting Trace Explosives Using SERS. *Nanomaterials (Basel)* **2018**, *8*, doi:10.3390/nano8110872.
16. Wang, H.; Han, X.; Ou, X.; Lee, C.S.; Zhang, X.; Lee, S.T. Silicon nanowire based single-molecule SERS sensor. *Nanoscale* **2013**, *5*, 8172-8176, doi:10.1039/c3nr01879b.
17. Novara, C.; Petracca, F.; Virga, A.; Rivolo, P.; Ferrero, S.; Chiolerio, A.; Geobaldo, F.; Porro, S.; Giorgis, F. SERS active silver nanoparticles synthesized by inkjet printing on mesoporous silicon. *Nanoscale Res Lett* **2014**, *9*, 527, doi:10.1186/1556-276X-9-527.
18. Panarin, A.Y.; Chirvony, V.S.; Kholostov, K.I.; Turpin, P.Y.; Terekhov, S.N. Formation of SERS-active silver structures on the surface of mesoporous silicon. *Journal of Applied Spectroscopy* **2009**, *76*, 280-287, doi:10.1007/s10812-009-9175-1.
19. Alwan, A.M.; Wali, L.A.; Yousif, A.A. Optimization of AgNPs/mesoPS Active Substrates for Ultra–Low Molecule Detection Process. *Silicon* **2018**, *10*, 2241-2251, doi:10.1007/s12633-018-9758-7.
20. Nirala, N.R.; Shtenberg, G. N-acetyl-β-D-glucosaminidase biomarker quantification in milk using Ag-porous Si SERS platform for mastitis severity evaluation. *Applied Surface Science* **2021**, *566*, doi:10.1016/j.apsusc.2021.150700.
21. Al-Syadi, A.M.; Faisal, M.; Harraz, F.A.; Jalalah, M.; Alsaiari, M. Immersion-plated palladium nanoparticles onto meso-porous silicon layer as novel SERS substrate for sensitive detection of imidacloprid pesticide. *Sci Rep* **2021**, *11*, 9174, doi:10.1038/s41598-021-88326-0.
22. Ke, N.H.; Tuan, D.A.; Thong, T.T.; Long, N.H.; Thanh, N.H.; Tuan Hung, L.V. Preparation of SERS Substrate with Ag Nanoparticles Covered on Pyramidal Si Structure for Abamectin Detection. *Plasmonics* **2021**, *16*, 2125-2137, doi:10.1007/s11468-021-01386-w.
23. Kumar, A.; Sharma, R.; Sharma, A.K.; Agarwal, A. A cost-effective identification of tobacco alkaloids using porous Si SERS substrates for forensic and bioanalytical applications. *SN Applied Sciences* **2019**, *1*, doi:10.1007/s42452-019-1539-4.
24. Yang, X.; Zhong, H.; Zhu, Y.; Shen, J.; Li, C. Ultrasensitive and recyclable SERS substrate based on Au-decorated Si nanowire arrays. *Dalton Trans* **2013**, *42*, 14324-14330, doi:10.1039/c3dt51686e.
25. Galopin, E.; Barbillat, J.; Coffinier, Y.; Szunerits, S.; Patriarche, G.; Boukherroub, R. Silicon nanowires coated with silver nanostructures as ultrasensitive interfaces for

surface-enhanced Raman spectroscopy. *ACS Appl Mater Interfaces* **2009**, *1*, 1396-1403, doi:10.1021/am900087s.
26. Zhou, L.; Zhou, J.; Feng, Z.; Wang, F.; Xie, S.; Bu, S. Immunoassay for tumor markers in human serum based on Si nanoparticles and SiC@Ag SERS-active substrate. *Analyst* **2016**, *141*, 2534-2541, doi:10.1039/c6an00003g.
27. Zhao, X.-Y.; Wang, G.; Hong, M. Hybrid structures of Fe3O4 and Ag nanoparticles on Si nanopillar arrays substrate for SERS applications. *Materials Chemistry and Physics* **2018**, *214*, 377-382, doi:10.1016/j.matchemphys.2018.04.082.
28. Li, J.F.; Ding, S.Y.; Yang, Z.L.; Bai, M.L.; Anema, J.R.; Wang, X.; Wang, A.; Wu, D.Y.; Ren, B.; Hou, S.M.; et al. Extraordinary enhancement of Raman scattering from pyridine on single crystal Au and Pt electrodes by shell-isolated Au nanoparticles. *J Am Chem Soc* **2011**, *133*, 15922-15925, doi:10.1021/ja2074533.
29. Tran, M.; Whale, A.; Padalkar, S. Exploring the Efficacy of Platinum and Palladium Nanostructures for Organic Molecule Detection via Raman Spectroscopy. *Sensors (Basel)* **2018**, *18*, 147, doi:10.3390/s18010147.
30. Kim, N.H.; Kim, K. Surface-enhanced resonance Raman scattering of rhodamine 6G on Pt nanoaggregates. *Journal of Raman Spectroscopy* **2005**, *36*, 623-628, doi:10.1002/jrs.1352.
31. Jabbar, A.A.; Alwan, A.M. Efficient detecting of TNT molecules using palladium nanoparticles/ cross shape pores like structure porous silicon. *Vibrational Spectroscopy* **2019**, *103*, doi:10.1016/j.vibspec.2019.102933.
32. Jabbar, A.A.; Alwan, A.M.; Zayer, M.Q.; Bohan, A.J. Efficient single cell monitoring of pathogenic bacteria using bimetallic nanostructures embedded in gradient porous silicon. *Materials Chemistry and Physics* **2020**, *241*, doi:10.1016/j.matchemphys.2019.122359.
33. Roy, A.; Singha, S.S.; Majumder, S.; Singha, A.; Banerjee, S.; Satpati, B. Electroless Deposition of Pd Nanostructures for Multifunctional Applications as Surface-Enhanced Raman Scattering Substrates and Electrochemical Nonenzymatic Sensors. *ACS Applied Nano Materials* **2019**, *2*, 2503-2514, doi:10.1021/acsanm.9b00420.
34. Hardiansyah, A.; Chen, A.Y.; Liao, H.L.; Yang, M.C.; Liu, T.Y.; Chan, T.Y.; Tsou, H.M.; Kuo, C.Y.; Wang, J.K.; Wang, Y.L. Core-shell of FePt@SiO2-Au magnetic nanoparticles for rapid SERS detection. *Nanoscale Res Lett* **2015**, *10*, 412, doi:10.1186/s11671-015-1111-0.
35. Lin, D.; Wu, Z.; Li, S.; Zhao, W.; Ma, C.; Wang, J.; Jiang, Z.; Zhong, Z.; Zheng, Y.; Yang, X. Large-Area Au-Nanoparticle-Functionalized Si Nanorod Arrays for Spatially Uniform Surface-Enhanced Raman Spectroscopy. *ACS Nano* **2017**, *11*, 1478-1487, doi:10.1021/acsnano.6b06778.
36. Quyen, T.T.B.; Chang, C.C.; Su, W.N.; Uen, Y.H.; Pan, C.J.; Liu, J.Y.; Rick, J.; Lin, K.Y.; Hwang, B.J. Self-focusing Au@SiO2 nanorods with rhodamine 6G as highly sensitive SERS substrate for carcinoembryonic antigen detection. *J Mater Chem B* **2014**, *2*, 629-636, doi:10.1039/c3tb21278e.
37. Quyen, T.T.B.; Su, W.-N.; Chen, K.-J.; Pan, C.-J.; Rick, J.; Chang, C.-C.; Hwang, B.-J. Au@SiO2
core/shell nanoparticle assemblage used for highly sensitive SERS-based determination of glucose and uric acid. *Journal of Raman Spectroscopy* **2013**, *44*, 1671-1677, doi:10.1002/jrs.4400.
38. Wang, T.; Zhang, Z.; Liao, F.; Cai, Q.; Li, Y.; Lee, S.T.; Shao, M. The effect of dielectric constants on noble metal/semiconductor SERS enhancement: FDTD simulation and experiment validation of Ag/Ge and Ag/Si substrates. *Sci Rep* **2014**, *4*, 4052, doi:10.1038/srep04052.

39. Nguyen, M.K.; Su, W.N.; Chen, C.H.; Rick, J.; Hwang, B.J. Highly sensitive and stable Ag@SiO2 nanocubes for label-free SERS-photoluminescence detection of biomolecules. *Spectrochim Acta A Mol Biomol Spectrosc* **2017**, *175*, 239-245, doi:10.1016/j.saa.2016.12.024.
40. Yang, Z.; Liu, H.; Tian, Y.; Chen, Y.; Niu, Z.; Zhou, C.; Wang, F.; Gu, C.; Tang, S.; Jiang, T.; et al. Synergistic effect of a "stellate" mesoporous SiO2@Au nanoprobe and coffee-ring-free hydrophilic–hydrophobic substrate assembly in an ultrasensitive SERS-based immunoassay for a tumor marker. *Journal of Materials Chemistry C* **2020**, *8*, 2142-2154, doi:10.1039/c9tc05646g.
41. He, Y.; Yang, X.; Yuan, R.; Chai, Y. A novel ratiometric SERS biosensor with one Raman probe for ultrasensitive microRNA detection based on DNA hydrogel amplification. *J Mater Chem B* **2019**, *7*, 2643-2647, doi:10.1039/c8tb02894j.
42. Shi, L.; Xu, L.; Xiao, R.; Zhou, Z.; Wang, C.; Wang, S.; Gu, B. Rapid, Quantitative, High-Sensitive Detection of Escherichia coli O157:H7 by Gold-Shell Silica-Core Nanospheres-Based Surface-Enhanced Raman Scattering Lateral Flow Immunoassay. *Front Microbiol* **2020**, *11*, 596005, doi:10.3389/fmicb.2020.596005.
43. Panikar, S.S.; Banu, N.; Escobar, E.R.; Garcia, G.R.; Cervantes-Martinez, J.; Villegas, T.C.; Salas, P.; De la Rosa, E. Stealth modified bottom up SERS substrates for label-free therapeutic drug monitoring of doxorubicin in blood serum. *Talanta* **2020**, *218*, 121138, doi:10.1016/j.talanta.2020.121138.
44. Chen, L.Y.; Yang, K.H.; Chen, H.C.; Liu, Y.C.; Chen, C.H.; Chen, Q.Y. Innovative fabrication of a Au nanoparticle-decorated SiO2 mask and its activity on surface-enhanced Raman scattering. *Analyst* **2014**, *139*, 1929-1937, doi:10.1039/c3an02089d.
45. Liu, Y.-C.; Yu, C.-C.; Hsu, T.-C. Improved performances on surface-enhanced Raman scattering based on electrochemically roughened gold substrates modified with SiO2 nanoparticles. *Journal of Raman Spectroscopy* **2009**, *40*, 1682-1686, doi:10.1002/jrs.2319.
46. Liu, Y.-C.; Yang, K.-H.; Hsu, T.-C. Improved Surface-Enhanced Raman Scattering Performances on Silver−Silica Nanocomposites. *The Journal of Physical Chemistry C* **2009**, *113*, 8162-8168, doi:10.1021/jp810503z.
47. Li, D.; Li, D.-W.; Li, Y.; Fossey, J.S.; Long, Y.-T. Cyclic electroplating and stripping of silver on Au@SiO2 core/shell nanoparticles for sensitive and recyclable substrate of surface-enhanced Raman scattering. *Journal of Materials Chemistry* **2010**, *20*, 3688-3693, doi:10.1039/b924865j.
48. Mekonnen, M.L.; Su, W.-N.; Chen, C.-H.; Hwang, B.-J. Ag@SiO2 nanocube loaded miniaturized filter paper as a hybrid flexible plasmonic SERS substrate for trace melamine detection. *Analytical Methods* **2017**, *9*, 6823-6829, doi:10.1039/c7ay02192e.
49. Huang, J.A.; Zhao, Y.Q.; Zhang, X.J.; He, L.F.; Wong, T.L.; Chui, Y.S.; Zhang, W.J.; Lee, S.T. Ordered Ag/Si nanowires array: wide-range surface-enhanced Raman spectroscopy for reproducible biomolecule detection. *Nano Lett* **2013**, *13*, 5039-5045, doi:10.1021/nl401920u.
50. Lu, H.; Jin, M.; Ma, Q.; Yan, Z.; Liu, Z.; Wang, X.; Akinoglu, E.M.; van den Berg, A.; Zhou, G.; Shui, L. Ag nano-assemblies on Si surface via CTAB-assisted galvanic reaction for sensitive and reliable surface-enhanced Raman scattering detection. *Sensors and Actuators B: Chemical* **2020**, *304*, doi:10.1016/j.snb.2019.127224.
51. Qiu, L.; Liu, Q.; Zeng, X.; Liu, Q.; Hou, X.; Tian, Y.; Wu, L. Sensitive detection of bisphenol A by coupling solid phase microextraction based on monolayer graphene-coated Ag nanoparticles on Si fibers to surface enhanced Raman spectroscopy. *Talanta* **2018**, *187*, 13-18, doi:10.1016/j.talanta.2018.05.001.
52. Li, H.; Yang, B.; Yu, B.; Huang, N.; Liu, L.; Lu, J.; Jiang, X. Graphene-coated Si nanowires as substrates for surface-enhanced Raman scattering. *Applied Surface Science* **2021**, *541*, doi:10.1016/j.apsusc.2020.148486.

53. Mikac, L.; Ivanda, M.; Đerek, V.; Gotić, M. Influence of mesoporous silicon preparation condition on silver clustering and SERS enhancement. *Journal of Raman Spectroscopy* **2016**, *47*, 1036-1041, doi:10.1002/jrs.4911.
54. D'Andrea, C.; Faro, M.J.; Bertino, G.; Ossi, P.M.; Neri, F.; Trusso, S.; Musumeci, P.; Galli, M.; Cioffi, N.; Irrera, A.; et al. Decoration of silicon nanowires with silver nanoparticles for ultrasensitive surface enhanced Raman scattering. *Nanotechnology* **2016**, *27*, 375603, doi:10.1088/0957-4484/27/37/375603.
55. Fahes, A.; En Naciri, A.; Navvabpour, M.; Jradi, S.; Akil, S. Self-Assembled Ag Nanocomposites into Ultra-Sensitive and Reproducible Large-Area SERS-Active Opaque Substrates. *Nanomaterials (Basel)* **2021**, *11*, doi:10.3390/nano11082055.
56. Guo, J.; Liu, G.; Ma, Q.; Yang, S.; Li, Y.; Cai, W. Fabrication of Ag-nanosheets-built micro/nanostructured arrays via in situ conversion on Cu2O-coated Si nanocone platform and their highly structurally-enhanced SERS effect. *Nanotechnology* **2019**, *30*, 345302, doi:10.1088/1361-6528/ab1f98.
57. Huang, T.; Cao, L.; Zhang, X.; Xiong, X.; Xu, J.; Xiao, R. A facile method to fabricate a novel 3D porous silicon/gold architecture for surface enhanced Raman scattering. *Journal of Alloys and Compounds* **2019**, *790*, 127-133, doi:10.1016/j.jallcom.2019.03.161.
58. Moram, S.S.B.; Shaik, A.K.; Byram, C.; Hamad, S.; Soma, V.R. Instantaneous trace detection of nitro-explosives and mixtures with nanotextured silicon decorated with Ag-Au alloy nanoparticles using the SERS technique. *Anal Chim Acta* **2020**, *1101*, 157-168, doi:10.1016/j.aca.2019.12.026.
59. Yang, J.; Li, J.; Du, Z.; Gong, Q.; Teng, J.; Hong, M. Laser hybrid micro/nano-structuring of Si surfaces in air and its applications for SERS detection. *Sci Rep* **2014**, *4*, 6657, doi:10.1038/srep06657.
60. Karadan, P.; Aggarwal, S.; Anappara, A.A.; Narayana, C.; Barshilia, H.C. Tailored periodic Si nanopillar based architectures as highly sensitive universal SERS biosensing platform. *Sensors and Actuators B: Chemical* **2018**, *254*, 264-271, doi:10.1016/j.snb.2017.07.088.
61. Zhang, C.; Lin, K.; Huang, Y.; Zhang, J. Graphene-Ag Hybrids on Laser-Textured Si Surface for SERS Detection. *Sensors (Basel)* **2017**, *17*, doi:10.3390/s17071462.
62. Li, S.; Zhang, N.; Zhang, N.; Lin, D.; Hu, X.; Yang, X. Three-dimensional ordered Ag/ZnO/Si hierarchical nanoflower arrays for spatially uniform and ultrasensitive SERS detection. *Sensors and Actuators B: Chemical* **2020**, *321*, doi:10.1016/j.snb.2020.128519.
63. Zhou, L.; Liu, Y.; Wang, F.; Jia, Z.; Zhou, J.; Jiang, T.; Petti, L.; Chen, Y.; Xiong, Q.; Wang, X. Classification analyses for prostate cancer, benign prostate hyperplasia and healthy subjects by SERS-based immunoassay of multiple tumour markers. *Talanta* **2018**, *188*, 238-244, doi:10.1016/j.talanta.2018.05.070.
64. Berus, S.M.; Adamczyk-Poplawska, M.; Mlynarczyk-Bonikowska, B.; Witkowska, E.; Szymborski, T.; Waluk, J.; Kaminska, A. SERS-based sensor for the detection of sexually transmitted pathogens in the male swab specimens: A new approach for clinical diagnosis. *Biosens Bioelectron* **2021**, *189*, 113358, doi:10.1016/j.bios.2021.113358.
65. Kaminska, A.; Witkowska, E.; Kowalska, A.; Skoczynska, A.; Gawryszewska, I.; Guziewicz, E.; Snigurenko, D.; Waluk, J. Highly efficient SERS-based detection of cerebrospinal fluid neopterin as a diagnostic marker of bacterial infection. *Anal Bioanal Chem* **2016**, *408*, 4319-4327, doi:10.1007/s00216-016-9535-7.
66. Meng, S.; Chen, R.; Xie, J.; Li, J.; Cheng, J.; Xu, Y.; Cao, H.; Wu, X.; Zhang, Q.; Wang, H. Surface-enhanced Raman scattering holography chip for rapid, sensitive and multiplexed detection of human breast cancer-associated MicroRNAs in clinical samples. *Biosens Bioelectron* **2021**, *190*, 113470, doi:10.1016/j.bios.2021.113470.

67. Chen, N.; Ding, P.; Shi, Y.; Jin, T.; Su, Y.; Wang, H.; He, Y. Portable and Reliable Surface-Enhanced Raman Scattering Silicon Chip for Signal-On Detection of Trace Trinitrotoluene Explosive in Real Systems. *Anal Chem* **2017**, *89*, 5072-5078, doi:10.1021/acs.analchem.7b00521.
68. Kunushpayeva, Z.; Rapikov, A.; Akhmetova, A.; Sultangaziyev, A.; Dossym, D.; Bukasov, R. Sandwich SERS immunoassay of human immunoglobulin on silicon wafer compared to traditional SERS substrate, gold film. *Sensing and Bio-Sensing Research* **2020**, *29*, 100355, doi:10.1016/j.sbsr.2020.100355.
69. Zhu, C.; Zhao, Q.; Huo, D.; Hu, X.; Wang, X. Electrodeposition of rough gold nanoarrays for surface-enhanced Raman scattering detection. *Materials Chemistry and Physics* **2021**, *263*, 124388, doi:10.1016/j.matchemphys.2021.124388.
70. Wang, J.; Qiu, C.; Mu, X.; Pang, H.; Chen, X.; Liu, D. Ultrasensitive SERS detection of rhodamine 6G and p-nitrophenol based on electrochemically roughened nano-Au film. *Talanta* **2020**, *210*, 120631, doi:10.1016/j.talanta.2019.120631.
71. Tegegne, W.A.; Su, W.-N.; Beyene, A.B.; Huang, W.-H.; Tsai, M.-C.; Hwang, B.-J. Flexible hydrophobic filter paper-based SERS substrate using silver nanocubes for sensitive and rapid detection of adenine. *Microchemical Journal* **2021**, *168*, 106349, doi:10.1016/j.microc.2021.106349.
72. Mitsai, E.; Kuchmizhak, A.; Pustovalov, E.; Sergeev, A.; Mironenko, A.; Bratskaya, S.; Linklater, D.P.; Balcytis, A.; Ivanova, E.; Juodkazis, S. Chemically non-perturbing SERS detection of a catalytic reaction with black silicon. *Nanoscale* **2018**, *10*, 9780-9787, doi:10.1039/c8nr02123f.
73. Sekhar, P.K.; Ramgir, N.S.; Bhansali, S. Metal-Decorated Silica Nanowires: An Active Surface-Enhanced Raman Substrate for Cancer Biomarker Detection. *The Journal of Physical Chemistry C* **2008**, *112*, 1729-1734, doi:10.1021/jp077698o.
74. Pellacani, P.; Torres-Costa, V.; Agullo-Rueda, F.; Vanna, R.; Morasso, C.; Manso Silvan, M. Laser writing of nanostructured silicon arrays for the SERS detection of biomolecules with inhibited oxidation. *Colloids Surf B Biointerfaces* **2019**, *174*, 174-180, doi:10.1016/j.colsurfb.2018.11.010.
75. Powell, J.A.; Venkatakrishnan, K.; Tan, B. Programmable SERS active substrates for chemical and biosensing applications using amorphous/crystalline hybrid silicon nanomaterial. *Sci Rep* **2016**, *6*, 19663, doi:10.1038/srep19663.
76. Keshavarz, M.; Tan, B.; Venkatakrishnan, K. Label-Free SERS Quantum Semiconductor Probe for Molecular-Level and in Vitro Cellular Detection: A Noble-Metal-Free Methodology. *ACS Appl Mater Interfaces* **2018**, *10*, 34886-34904, doi:10.1021/acsami.8b10590.
77. Yu, C.Y.; Chung, C.K. Novel irregular pore peripheral plasmonic mechanism of nanocomposite metal-nanoporous AAO using new facile one-step anodization and pore widening for high SERS enhancement. *Applied Surface Science* **2022**, *580*, doi:10.1016/j.apsusc.2021.152252.
78. Wang, T.; Zhou, J.; Wang, Y. Simple, Low-Cost Fabrication of Highly Uniform and Reproducible SERS Substrates Composed of Ag(-)Pt Nanoparticles. *Nanomaterials (Basel)* **2018**, *8*, doi:10.3390/nano8050331.
79. Tian, Z.-Q.; Ren, B.; Wu, D.-Y. Surface-Enhanced Raman Scattering: From Noble to Transition Metals and from Rough Surfaces to Ordered Nanostructures. *The Journal of Physical Chemistry B* **2002**, *106*, 9463-9483, doi:10.1021/jp0257449.
80. Kim, K.; Lee, H.B.; Yoon, J.K.; Shin, D.; Shin, K.S. Ag Nanoparticle-Mediated Raman Scattering of 4-Aminobenzenethiol on a Pt Substrate. *The Journal of Physical Chemistry C* **2010**, *114*, 13589-13595, doi:10.1021/jp105005a.
81. Cai, J.; Huang, J.; Ge, M.; Iocozzia, J.; Lin, Z.; Zhang, K.Q.; Lai, Y. Immobilization of Pt Nanoparticles via Rapid and Reusable Electropolymerization of Dopamine on TiO2

Nanotube Arrays for Reversible SERS Substrates and Nonenzymatic Glucose Sensors. *Small* **2017**, *13*, 1604240, doi:10.1002/smll.201604240.
82. Zhu, S.; Fan, C.; Wang, J.; He, J.; Liang, E.; Chao, M. Surface enhanced Raman scattering of 4-aminothiophenol sandwiched between Ag nanocubes and smooth Pt substrate: The effect of the thickness of Pt film. *Journal of Applied Physics* **2014**, *116*, doi:10.1063/1.4891453.
83. Hu, J.; Chen, S.; Johnson, R.P.; Lin, X.; Yang, Z.; Russell, A.E. Surface-Enhanced Raman Scattering on Uniform Pd and Pt Films: From Ill-Defined to Structured Surfaces. *The Journal of Physical Chemistry C* **2013**, *117*, 24843-24850, doi:10.1021/jp4081433.
84. Fang, P.-P.; Li, J.-F.; Yang, Z.-L.; Li, L.-M.; Ren, B.; Tian, Z.-Q. Optimization of SERS activities of gold nanoparticles and gold-core-palladium-shell nanoparticles by controlling size and shell thickness. *Journal of Raman Spectroscopy* **2008**, *39*, 1679-1687, doi:10.1002/jrs.2066.
85. Shvalya, V.; Filipič, G.; Vengust, D.; Zavašnik, J.; Modic, M.; Abdulhalim, I.; Cvelbar, U. Reusable Au/Pd-coated chestnut-like copper oxide SERS substrates with ultra-fast self-recovery. *Applied Surface Science* **2020**, *517*, doi:10.1016/j.apsusc.2020.146205.
86. Kundu, S.; Yi, S.I.; Ma, L.; Chen, Y.; Dai, W.; Sinyukov, A.M.; Liang, H. Morphology dependent catalysis and surface enhanced Raman scattering (SERS) studies using Pd nanostructures in DNA, CTAB and PVA scaffolds. *Dalton Trans* **2017**, *46*, 9678-9691, doi:10.1039/c7dt01474k.
87. Feng, J.-J.; Lin, X.-X.; Chen, S.-S.; Huang, H.; Wang, A.-J. Thymine-directed synthesis of highly branched gold-palladium alloy nanobrambles as a highly active surface-enhanced Raman scattering substrate. *Sensors and Actuators B: Chemical* **2017**, *247*, 490-497, doi:10.1016/j.snb.2017.03.053.
88. Jiao, W.; Chen, C.; You, W.; Zhao, X.; Zhang, J.; Feng, Y.; Wang, P.; Che, R. Hollow Palladium-Gold Nanochains with Periodic Concave Structures as Superior ORR Electrocatalysts and Highly Efficient SERS Substrates. *Advanced Energy Materials* **2020**, *10*, doi:10.1002/aenm.201904072.
89. Choi, S.; Jeong, H.; Choi, K.H.; Song, J.Y.; Kim, J. Electrodeposition of triangular Pd rod nanostructures and their electrocatalytic and SERS activities. *ACS Appl Mater Interfaces* **2014**, *6*, 3002-3007, doi:10.1021/am405601g.
90. Choi, S.; Kweon, S.; Kim, J. Electrodeposition of Pt nanostructures with reproducible SERS activity and superhydrophobicity. *Phys Chem Chem Phys* **2015**, *17*, 23547-23553, doi:10.1039/c5cp04261e.
91. Bhuvana, T.; Kulkarni, G.U. A SERS-active nanocrystalline pd substrate and its nanopatterning leading to biochip fabrication. *Small* **2008**, *4*, 670-676, doi:10.1002/smll.200701075.
92. Ma, Z.-C.; Zhang, Y.-L.; Han, B.; Liu, X.-Q.; Zhang, H.-Z.; Chen, Q.-D.; Sun, H.-B. Femtosecond Laser Direct Writing of Plasmonic Ag/Pd Alloy Nanostructures Enables Flexible Integration of Robust SERS Substrates. *Advanced Materials Technologies* **2017**, *2*, doi:10.1002/admt.201600270.
93. Li, Y.; Lu, G.; Wu, X.; Shi, G. Electrochemical fabrication of two-dimensional palladium nanostructures as substrates for surface enhanced Raman scattering. *J Phys Chem B* **2006**, *110*, 24585-24592, doi:10.1021/jp0638787.
94. Jeong, H.; Kim, J. Electrodeposition of nanoflake Pd structures: structure-dependent wettability and SERS activity. *ACS Appl Mater Interfaces* **2015**, *7*, 7129-7135, doi:10.1021/acsami.5b02113.
95. Liu, Z.; Yang, Z.-L.; Cui, L.; Ren, B.; Tian, Z.-Q. Electrochemically Roughened Palladium Electrodes for Surface-Enhanced Raman Spectroscopy: Methodology, Mechanism, and Application. *The Journal of Physical Chemistry C* **2007**, *111*, 1770-1775, doi:10.1021/jp066122g.

96. Bich Quyen, T.T.; Su, W.N.; Chen, C.H.; Rick, J.; Liu, J.Y.; Hwang, B.J. Novel Ag/Au/Pt trimetallic nanocages used with surface-enhanced Raman scattering for trace fluorescent dye detection. *J Mater Chem B* **2014**, *2*, 5550-5557, doi:10.1039/c4tb00569d.
97. Zhu, S.; Fan, C.; Mao, Y.; Wang, J.; He, J.; Liang, E.; Chao, M. A monolayer of hierarchical silver hemi-mesoparticles with tunable surface topographies for highly sensitive surface-enhanced Raman spectroscopy. *J Chem Phys* **2016**, *144*, 074703, doi:10.1063/1.4941699.
98. He, L.; Huang, J.; Xu, T.; Chen, L.; Zhang, K.; Han, S.; He, Y.; Lee, S.T. Silver nanosheet-coated inverse opal film as a highly active and uniform SERS substrate. *J. Mater. Chem.* **2012**, *22*, 1370-1374, doi:10.1039/c1jm14144a.
99. Fang, C.; Brodoceanu, D.; Kraus, T.; Voelcker, N.H. Templated silver nanocube arrays for single-molecule SERS detection. *RSC Advances* **2013**, *3*, 4288-4293, doi:10.1039/c3ra22457k.
100. Li, J.; Zhou, J.; Jiang, T.; Wang, B.; Gu, M.; Petti, L.; Mormile, P. Controllable synthesis and SERS characteristics of hollow sea-urchin gold nanoparticles. *Phys Chem Chem Phys* **2014**, *16*, 25601-25608, doi:10.1039/c4cp04017a.
101. Liu, H.; Zhang, L.; Lang, X.; Yamaguchi, Y.; Iwasaki, H.; Inouye, Y.; Xue, Q.; Chen, M. Single molecule detection from a large-scale SERS-active Au(7)(9)Ag(2)(1) substrate. *Sci Rep* **2011**, *1*, 112, doi:10.1038/srep00112.
102. Beyene, A.B.; Hwang, B.J.; Tegegne, W.A.; Wang, J.-S.; Tsai, H.-C.; Su, W.-N. Reliable and sensitive detection of pancreatic cancer marker by gold nanoflower-based SERS mapping immunoassay. *Microchemical Journal* **2020**, *158*, doi:10.1016/j.microc.2020.105099.
103. Quyen, T.T.B.a.H.B.J. Novel Ag/Au Nanocubes Modified the Negative/Positive Charge on the Surface and Their Application in Surface-Enhanced Raman Scattering. In Proceedings of the Procedia CIRP, 2016; pp. 551-556.
104. Dasary, S.S.; Singh, A.K.; Senapati, D.; Yu, H.; Ray, P.C. Gold nanoparticle based label-free SERS probe for ultrasensitive and selective detection of trinitrotoluene. *J Am Chem Soc* **2009**, *131*, 13806-13812, doi:10.1021/ja905134d.
105. Marrapu, H.; Avasarala, R.; Soma, V.R.; Balivada, S.K.; Podagatlapalli, G.K. Silver nanoribbons achieved by picosecond ablation using cylindrical focusing and SERS-based trace detection of TNT. *RSC Advances* **2020**, *10*, 41217-41228, doi:10.1039/d0ra05942k.
106. Shi, G.; Wang, M.; Zhu, Y.; Wang, Y.; Ma, W. Synthesis of flexible and stable SERS substrate based on Au nanofilms/cicada wing array for rapid detection of pesticide residues. *Optics Communications* **2018**, *425*, 49-57, doi:10.1016/j.optcom.2018.04.065.
107. Chen, S.; Liu, B.; Zhang, X.; Mo, Y.; Chen, F.; Shi, H.; Zhang, W.; Hu, C.; Chen, J. Electrochemical fabrication of pyramid-shape silver microstructure as effective and reusable SERS substrate. *Electrochimica Acta* **2018**, *274*, 242-249, doi:10.1016/j.electacta.2018.04.120.
108. Chang, C.C.; Yang, K.H.; Liu, Y.C.; Yu, C.C.; Wu, Y.H. Surface-enhanced Raman scattering-active gold nanoparticles modified with a monolayer of silver film. *Analyst* **2012**, *137*, 4943-4950, doi:10.1039/c2an35912j.
109. Mai, F.D.; Hsu, T.C.; Liu, Y.C.; Yang, K.H.; Chen, B.C. A new strategy to prepare surface-enhanced Raman scattering-active substrates by electrochemical pulse deposition of gold nanoparticles. *Chem Commun (Camb)* **2011**, *47*, 2958-2960, doi:10.1039/c0cc05262k.
110. Huang, D.; Zhao, J.; Wang, M.; Zhu, S. Snowflake-like gold nanoparticles as SERS substrates for the sensitive detection of organophosphorus pesticide residues. *Food Control* **2020**, *108*, 106835, doi:10.1016/j.foodcont.2019.106835.

111. Purwidyantri, A.; El-Mekki, I.; Lai, C. Tunable plasmonic Au-film over nanosphere SERS substrate by rapid thermal annealing. In Proceedings of the 2016 IEEE 16th International Conference on Nanotechnology (IEEE-NANO), 22-25 Aug. 2016, 2016; pp. 323-324.
112. Guo, L.; Zhang, C.X.; Deng, L.; Zhang, G.X.; Xu, H.J.; Sun, X.M. Cicada wing decorated by silver nanoparticles as low-cost and active/sensitive substrates for surface-enhanced Raman scattering. *Journal of Applied Physics* **2014**, *115*, 213101, doi:10.1063/1.4880956.
113. Rajapandiyan, P.; Yang, J. Photochemical method for decoration of silver nanoparticles on filter paper substrate for SERS application. *Journal of Raman Spectroscopy* **2014**, *45*, 574-580, doi:10.1002/jrs.4502.
114. Potara, M.; Baia, M.; Farcau, C.; Astilean, S. Chitosan-coated anisotropic silver nanoparticles as a SERS substrate for single-molecule detection. *Nanotechnology* **2012**, *23*, 055501, doi:10.1088/0957-4484/23/5/055501.
115. Hou, C.; Meng, G.; Huang, Q.; Zhu, C.; Huang, Z.; Chen, B.; Sun, K. Ag-nanoparticle-decorated Au-fractal patterns on bowl-like-dimple arrays on Al foil as an effective SERS substrate for the rapid detection of PCBs. *Chem Commun (Camb)* **2014**, *50*, 569-571, doi:10.1039/c3cc46878j.
116. Sui, C.; Wang, K.; Wang, S.; Ren, J.; Bai, X.; Bai, J. SERS activity with tenfold detection limit optimization on a type of nanoporous AAO-based complex multilayer substrate. *Nanoscale* **2016**, *8*, 5920-5927, doi:10.1039/c5nr06771e.
117. Nielsen, P.; Hassing, S.; Albrektsen, O.; Foghmoes, S.; Morgen, P. Fabrication of Large-Area Self-Organizing Gold Nanostructures with Sub-10 nm Gaps on a Porous Al2O3 Template for Application as a SERS-Substrate. *The Journal of Physical Chemistry C* **2009**, *113*, 14165-14171, doi:10.1021/jp9039012.
118. Choi, D.; Choi, Y.; Hong, S.; Kang, T.; Lee, L.P. Self-organized hexagonal-nanopore SERS array. *Small* **2010**, *6*, 1741-1744, doi:10.1002/smll.200901937.
119. Chang, Y.-C.; Wu, S.-H. Bi-functional Al-doped ZnO@SnO2 heteronanowires as efficient substrates for improving photocatalytic and SERS performance. *Journal of Industrial and Engineering Chemistry* **2019**, *76*, 333-343, doi:10.1016/j.jiec.2019.03.058.
120. Shan, D.; Huang, L.; Li, X.; Zhang, W.; Wang, J.; Cheng, L.; Feng, X.; Liu, Y.; Zhu, J.; Zhang, Y. Surface Plasmon Resonance and Interference Coenhanced SERS Substrate of AAO/Al-Based Ag Nanostructure Arrays. *The Journal of Physical Chemistry C* **2014**, *118*, 23930-23936, doi:10.1021/jp5026152.
121. Rajkumar, P.; Sarma, B.K. Ag/ZnO heterostructure fabricated on AZO platform for SERS based sensitive detection of biomimetic hydroxyapatite. *Applied Surface Science* **2020**, *509*, 144798, doi:10.1016/j.apsusc.2019.144798.
122. Das, S.; Goswami, L.P.; Gayathri, J.; Tiwari, S.; Saxena, K.; Mehta, D.S. Fabrication of low cost highly structured silver capped aluminium nanorods as SERS substrate for the detection of biological pathogens. *Nanotechnology* **2021**, *32*, 495301, doi:10.1088/1361-6528/ac2097.
123. Zhang, Y.; Liu, R.J.; Ma, X.; Liu, X.Y.; Zhang, Y.X.; Zhang, J. Ag nanoparticle decorated MnO2 flakes as flexible SERS substrates for rhodamine 6G detection. *RSC Advances* **2018**, *8*, 37750-37756, doi:10.1039/c8ra07778a.
124. Fang, Y.; Lin, T.; Zheng, D.; Zhu, Y.; Wang, L.; Fu, Y.; Wang, H.; Wu, X.; Zhang, P. Rapid and label-free identification of different cancer types based on surface-enhanced Raman scattering profiles and multivariate statistical analysis. *J Cell Biochem* **2021**, *122*, 277-289, doi:10.1002/jcb.29857.
125. Li, H.; Wang, Q.; Tang, J.; Gao, N.; Yue, X.; Zhong, F.; Lv, X.; Fu, J.; Wang, T.; Ma, C. Establishment of a reliable scheme for obtaining highly stable SERS signal of biological serum. *Biosens Bioelectron* **2021**, *189*, 113315, doi:10.1016/j.bios.2021.113315.

126. Tan, Y.; Yan, B.; Xue, L.; Li, Y.; Luo, X.; Ji, P. Surface-enhanced Raman spectroscopy of blood serum based on gold nanoparticles for the diagnosis of the oral squamous cell carcinoma. *Lipids Health Dis* **2017**, *16*, 73, doi:10.1186/s12944-017-0465-y.
127. Lin, D.; Feng, S.; Pan, J.; Chen, Y.; Lin, J.; Chen, G.; Xie, S.; Zeng, H.; Chen, R. Colorectal cancer detection by gold nanoparticle based surface-enhanced Raman spectroscopy of blood serum and statistical analysis. *Opt. Express* **2011**, *19*, 13565-13577, doi:10.1364/OE.19.013565.
128. Qian, K.; Wang, Y.; Hua, L.; Chen, A.; Zhang, Y. New method of lung cancer detection by saliva test using surface-enhanced Raman spectroscopy. *Thorac. Cancer* **2018**, *9*, 1556-1561, doi:10.1111/1759-7714.12837.
129. Del Mistro, G.; Cervo, S.; Mansutti, E.; Spizzo, R.; Colombatti, A.; Belmonte, P.; Zucconelli, R.; Steffan, A.; Sergo, V.; Bonifacio, A. Surface-enhanced Raman spectroscopy of urine for prostate cancer detection: a preliminary study. *Anal Bioanal Chem* **2015**, *407*, 3271-3275, doi:10.1007/s00216-015-8610-9.
130. Hong, Y.; Li, Y.; Huang, L.; He, W.; Wang, S.; Wang, C.; Zhou, G.; Chen, Y.; Zhou, X.; Huang, Y.; et al. Label-free diagnosis for colorectal cancer through coffee ring-assisted surface-enhanced Raman spectroscopy on blood serum. *J. Biophotonics* **2020**, *13*, e201960176, doi:10.1002/jbio.201960176.
131. Wang, J.; Lin, D.; Lin, J.; Yu, Y.; Huang, Z.; Chen, Y.; Lin, J.; Feng, S.; Li, B.; Liu, N.; et al. Label-free detection of serum proteins using surface-enhanced Raman spectroscopy for colorectal cancer screening. *J Biomed Opt* **2014**, *19*, 087003, doi:10.1117/1.JBO.19.8.087003.
132. Yu, Y.; Lin, Y.; Xu, C.; Lin, K.; Ye, Q.; Wang, X.; Xie, S.; Chen, R.; Lin, J. Label-free detection of nasopharyngeal and liver cancer using surface-enhanced Raman spectroscopy and partial lease squares combined with support vector machine. *Biomed Opt Express* **2018**, *9*, 6053-6066, doi:10.1364/BOE.9.006053.
133. Li, B.; Ding, H.; Wang, Z.; Liu, Z.; Cai, X.; Yang, H. Research on the difference between patients with coronary heart disease and healthy controls by surface enhanced Raman spectroscopy. *Spectrochim Acta A Mol Biomol Spectrosc* **2022**, *272*, 120997, doi:10.1016/j.saa.2022.120997.
134. Chen, N.; Rong, M.; Shao, X.; Zhang, H.; Liu, S.; Dong, B.; Xue, W.; Wang, T.; Li, T.; Pan, J. Surface-enhanced Raman spectroscopy of serum accurately detects prostate cancer in patients with prostate-specific antigen levels of 4-10 ng/mL. *Int J Nanomedicine* **2017**, *12*, 5399-5407, doi:10.2147/IJN.S137756.
135. Hernández-Arteaga, A.; de Jesús Zermeño Nava, J.; Kolosovas-Machuca, E.S.; Velázquez-Salazar, J.J.; Vinogradova, E.; José-Yacamán, M.; Navarro-Contreras, H.R. Diagnosis of breast cancer by analysis of sialic acid concentrations in human saliva by surface-enhanced Raman spectroscopy of silver nanoparticles. *Nano Research* **2017**, *10*, 3662-3670, doi:10.1007/s12274-017-1576-5.
136. Liang, J.; Teng, P.; Xiao, W.; He, G.; Song, Q.; Zhang, Y.; Peng, B.; Li, G.; Hu, L.; Cao, D.; et al. Application of the amplification-free SERS-based CRISPR/Cas12a platform in the identification of SARS-CoV-2 from clinical samples. *J. Nanobiotechnology* **2021**, *19*, 273, doi:10.1186/s12951-021-01021-0.
137. Stefancu, A.; Moisoiu, V.; Couti, R.; Andras, I.; Rahota, R.; Crisan, D.; Pavel, I.E.; Socaciu, C.; Leopold, N.; Crisan, N. Combining SERS analysis of serum with PSA levels for improving the detection of prostate cancer. *Nanomedicine* **2018**, *13*, 2455-2467, doi:10.2217/nnm-2018-0127.
138. Chang, T.-H.; Chang, Y.-C.; Wu, S.-H. Ag nanoparticles decorated ZnO: Al nanoneedles as a high-performance surface-enhanced Raman scattering substrate. *Journal of Alloys and Compounds* **2020**, *843*, doi:10.1016/j.jallcom.2020.156044.

139. Fodjo, E.K.; Riaz, S.; Li, D.-W.; Qu, L.-L.; Marius, N.P.; Albert, T.; Long, Y.-T. Cu@Ag/β-AgVO3 as a SERS substrate for the trace level detection of carbamate pesticides. *Analytical Methods* **2012**, *4*, 3785-3791, doi:10.1039/c2ay25635e.
140. Dai, P.; Li, H.; Huang, X.; Wang, N.; Zhu, L. Highly Sensitive and Stable Copper-Based SERS Chips Prepared by a Chemical Reduction Method. *Nanomaterials (Basel)* **2021**, *11*, 2770, doi:10.3390/nano11102770.
141. Liu, C.; Yang, M.; Yu, J.; Lei, F.; Wei, Y.; Peng, Q.; Li, C.; Li, Z.; Zhang, C.; Man, B. Fast multiphase analysis: Self-separation of mixed solution by a wettability-controlled CuO@Ag SERS substrate and its applications in pollutant detection. *Sensors and Actuators B: Chemical* **2020**, *307*, doi:10.1016/j.snb.2020.127663.
142. Rao, V.K.; Ghildiyal, P.; Radhakrishnan, T.P. In Situ Fabricated Cu–Ag Nanoparticle-Embedded Polymer Thin Film as an Efficient Broad Spectrum SERS Substrate. *The Journal of Physical Chemistry C* **2017**, *121*, 1339-1348, doi:10.1021/acs.jpcc.6b10238.
143. Zhang, M.; Zheng, Z.; Liu, H.; Wang, D.; Chen, T.; Liu, J.; Wu, Y. Rationally Designed Graphene/Bilayer Silver/Cu Hybrid Structure with Improved Sensitivity and Stability for Highly Efficient SERS Sensing. *ACS Omega* **2018**, *3*, 5761-5770, doi:10.1021/acsomega.8b00565.
144. Kaja, S.; Nag, A. Bimetallic Ag-Cu Alloy Microflowers as SERS Substrates with Single-Molecule Detection Limit. *Langmuir* **2021**, *37*, 13027-13037, doi:10.1021/acs.langmuir.1c02119.
145. Fu, P.; Shi, X.; Jiang, F.; Xu, X. Superhydrophobic nanostructured copper substrate as sensitive SERS platform prepared by femtosecond laser pulses. *Applied Surface Science* **2020**, *501*, doi:10.1016/j.apsusc.2019.144269.
146. Bańkowska, M.; Krajczewski, J.; Dzięcielewski, I.; Kudelski, A.; Weyher, J.L. Au–Cu Alloyed Plasmonic Layer on Nanostructured GaN for SERS Application. *The Journal of Physical Chemistry C* **2016**, *120*, 1841-1846, doi:10.1021/acs.jpcc.5b11371.
147. Sakir, M.; Yilmaz, E.; Onses, M.S. SERS-active hydrophobic substrates fabricated by surface growth of Cu nanostructures. *Microchemical Journal* **2020**, *154*, doi:10.1016/j.microc.2020.104628.
148. Chen, K.; Zhang, X.; MacFarlane, D.R. Ultrasensitive surface-enhanced Raman scattering detection of urea by highly ordered Au/Cu hybrid nanostructure arrays. *Chem Commun (Camb)* **2017**, *53*, 7949-7952, doi:10.1039/c7cc03523c.
149. Singh, M.K.; Chettri, P.; Basu, J.; Tripathi, A.; Mukherjee, B.; Tiwari, A.; Mandal, R.K. Synthesis of anisotropic Au–Cu alloy nanostructures and its application in SERS for detection of methylene blue. *Materials Research Express* **2020**, *7*, 015052, doi:10.1088/2053-1591/ab63c7.
150. Kumar-Krishnan, S.; Esparza, R.; Pal, U. Controlled Fabrication of Flower-Shaped Au-Cu Nanostructures Using a Deep Eutectic Solvent and Their Performance in Surface-Enhanced Raman Scattering-Based Molecular Sensing. *ACS Omega* **2020**, *5*, 3699-3708, doi:10.1021/acsomega.9b04355.
151. Li, Z.; Li, C.; Yu, J.; Li, Z.; Zhao, X.; Liu, A.; Jiang, S.; Yang, C.; Zhang, C.; Man, B. Aluminum nanoparticle films with an enhanced hot-spot intensity for high-efficiency SERS. *Opt. Express* **2020**, *28*, 9174-9185, doi:10.1364/OE.389886.
152. Tian, S.; Neumann, O.; McClain, M.J.; Yang, X.; Zhou, L.; Zhang, C.; Nordlander, P.; Halas, N.J. Aluminum Nanocrystals: A Sustainable Substrate for Quantitative SERS-Based DNA Detection. *Nano Lett* **2017**, *17*, 5071-5077, doi:10.1021/acs.nanolett.7b02338.
153. Lay, C.L.; Koh, C.S.L.; Wang, J.; Lee, Y.H.; Jiang, R.; Yang, Y.; Yang, Z.; Phang, I.Y.; Ling, X.Y. Aluminum nanostructures with strong visible-range SERS activity for versatile micropatterning of molecular security labels. *Nanoscale* **2018**, *10*, 575-581, doi:10.1039/c7nr07793a.

154. Sharma, B.; Cardinal, M.F.; Ross, M.B.; Zrimsek, A.B.; Bykov, S.V.; Punihaole, D.; Asher, S.A.; Schatz, G.C.; Van Duyne, R.P. Aluminum Film-Over-Nanosphere Substrates for Deep-UV Surface-Enhanced Resonance Raman Spectroscopy. *Nano Lett* **2016**, *16*, 7968-7973, doi:10.1021/acs.nanolett.6b04296.
155. Ding, T.; Sigle, D.O.; Herrmann, L.O.; Wolverson, D.; Baumberg, J.J. Nanoimprint lithography of Al nanovoids for deep-UV SERS. *ACS Appl Mater Interfaces* **2014**, *6*, 17358-17363, doi:10.1021/am505511v.
156. Sigle, D.O.; Perkins, E.; Baumberg, J.J.; Mahajan, S. Reproducible Deep-UV SERRS on Aluminum Nanovoids. *J Phys Chem Lett* **2013**, *4*, 1449-1452, doi:10.1021/jz4004813.
157. Li, L.; Fang Lim, S.; Puretzky, A.A.; Riehn, R.; Hallen, H.D. Near-field enhanced ultraviolet resonance Raman spectroscopy using aluminum bow-tie nano-antenna. *Appl Phys Lett* **2012**, *101*, 113116, doi:10.1063/1.4746747.
158. Liu, H.; Dai, E.; Xiao, R.; Zhou, Z.; Zhang, M.; Bai, Z.; Shao, Y.; Qi, K.; Tu, J.; Wang, C.; et al. Development of a SERS-based lateral flow immunoassay for rapid and ultra-sensitive detection of anti-SARS-CoV-2 IgM/IgG in clinical samples. *Sens Actuators B Chem* **2021**, *329*, 129196, doi:10.1016/j.snb.2020.129196.
159. Zhang, K.; Liu, X.; Man, B.; Yang, C.; Zhang, C.; Liu, M.; Zhang, Y.; Liu, L.; Chen, C. Label-free and stable serum analysis based on Ag-NPs/PSi surface-enhanced Raman scattering for noninvasive lung cancer detection. *Biomed Opt Express* **2018**, *9*, 4345-4358, doi:10.1364/BOE.9.004345.
160. Ma, X.; Cheng, H.; Hou, J.; Jia, Z.; Wu, G.; Lü, X.; Li, H.; Zheng, X.; Chen, C. Detection of breast cancer based on novel porous silicon Bragg reflector surface-enhanced Raman spectroscopy-active structure. *Chinese Optics Letters* **2020**, *18*, 051701, doi:10.3788/col202018.051701.
161. Cheng, N.; Lou, B.; Wang, H. An intelligent serological SERS test toward early-stage hepatocellular carcinoma diagnosis through ultrasensitive nanobiosensing. *Nano Research* **2022**, *15*, 5331-5339, doi:10.1007/s12274-022-4114-z.
162. Wang, Z.; Hong, Y.; Yan, H.; Luo, H.; Zhang, Y.; Li, L.; Lu, S.; Chen, Y.; Wang, D.; Su, Y.; et al. Fabrication of optoplasmonic particles through electroless deposition and the application in SERS-based screening of nodule-involved lung cancer. *Spectrochimica Acta Part A: Molecular and Biomolecular Spectroscopy* **2022**, *279*, 121483, doi:https://doi.org/10.1016/j.saa.2022.121483.
163. Weng, S.; Lin, D.; Lai, S.; Tao, H.; Chen, T.; Peng, M.; Qiu, S.; Feng, S. Highly sensitive and reliable detection of microRNA for clinically disease surveillance using SERS biosensor integrated with catalytic hairpin assembly amplification technology. *Biosensors and Bioelectronics* **2022**, *208*, 114236, doi:https://doi.org/10.1016/j.bios.2022.114236.
164. Jayakumar, P.; Kapil, D.; Pyng, L.; Hann Qian, L.; Dinish, U.S.; Malini, O. Proof of concept clinical study for the rapid diagnosis of Lung cancer from pleural fluid using label free SERS based chemometric approach. In Proceedings of the Proc.SPIE, 2021.
165. Buse, B.; Hülya, T.; Müslüm, İ.; Cenk, Y.; Sükrü Numan, B.; Süleyman, Ç.; Meriç, Ö.; Özlem, D.; Önder, E.; Ihsan, S.; et al. Clinical validation of SERS metasurface SARS-CoV-2 biosensor. In Proceedings of the Proc.SPIE, 2022.
166. Czaplicka, M.; Kowalska, A.A.; Nowicka, A.B.; Kurzydłowski, D.; Gronkiewicz, Z.; Machulak, A.; Kukwa, W.; Kamińska, A. Raman spectroscopy and surface-enhanced Raman spectroscopy (SERS) spectra of salivary glands carcinoma, tumor and healthy tissues and their homogenates analyzed by chemometry: Towards development of the novel tool for clinical diagnosis. *Analytica Chimica Acta* **2021**, *1177*, 338784, doi:https://doi.org/10.1016/j.aca.2021.338784.
167. Jenkins, C.A.; Jenkins, R.A.; Pryse, M.M.; Welsby, K.A.; Jitsumura, M.; Thornton, C.A.; Dunstan, P.R.; Harris, D.A. A high-throughput serum Raman spectroscopy platform and

methodology for colorectal cancer diagnostics. *Analyst* **2018**, *143*, 6014-6024, doi:10.1039/c8an01323c.

168. Li, S.; Zhang, Y.; Xu, J.; Li, L.; Zeng, Q.; Lin, L.; Guo, Z.; Liu, Z.; Xiong, H.; Liu, S. Noninvasive prostate cancer screening based on serum surface-enhanced Raman spectroscopy and support vector machine. *Applied Physics Letters* **2014**, *105*, 091104, doi:10.1063/1.4892667.
169. Chen, S.; Zhu, S.; Cui, X.; Xu, W.; Kong, C.; Zhang, Z.; Qian, W. Identifying non-muscle-invasive and muscle-invasive bladder cancer based on blood serum surface-enhanced Raman spectroscopy. *Biomed Opt Express* **2019**, *10*, 3533-3544, doi:10.1364/BOE.10.003533.
170. Liu, K.; Jin, S.; Song, Z.; Jiang, L.; Ma, L.; Zhang, Z. Label-free surface-enhanced Raman spectroscopy of serum based on multivariate statistical analysis for the diagnosis and staging of lung adenocarcinoma. *Vibrational Spectroscopy* **2019**, *100*, 177-184, doi:shttps://doi.org/10.1016/j.vibspec.2018.12.007.
171. Zhang, Y.; Lai, X.; Zeng, Q.; Li, L.; Lin, L.; Li, S.; Liu, Z.; Su, C.; Qi, M.; Guo, Z. Classifying low-grade and high-grade bladder cancer using label-free serum surface-enhanced Raman spectroscopy and support vector machine. *Laser Physics* **2018**, *28*, 035603, doi:10.1088/1555-6611/aa9d6d.
172. Li, S.; Li, L.; Zeng, Q.; Zhang, Y.; Guo, Z.; Liu, Z.; Jin, M.; Su, C.; Lin, L.; Xu, J.; et al. Characterization and noninvasive diagnosis of bladder cancer with serum surface enhanced Raman spectroscopy and genetic algorithms. *Sci Rep* **2015**, *5*, 9582, doi:10.1038/srep09582.
173. Xin, Z.; Tingting, L.; Yamin, L.; Jiamin, G.; Wei, G.; Xiang, W.; Yun, Y.; Juqiang, L. Label-free detection of prostate cancer and benign prostatic hyperplasia based on SERS spectroscopy of Plasma. In Proceedings of the Proc.SPIE, 2021.
